# Total Chemical Synthesis of Interleukin‐15 and Interleukin‐2: Taming Protein Hydrophobicity and Aggregation by a Versatile Solubilizing Strategy

**DOI:** 10.1002/anie.2586132

**Published:** 2026-05-25

**Authors:** Jingwen Zeng, Haiyan Zhou, Wang Xia, Hongxiang Wu, Xuechen Li

**Affiliations:** ^1^ Department of Chemistry State Key Laboratory of Synthetic Chemistry The University of Hong Kong Hong Kong SAR P. R. China; ^2^ Laboratory For Marine Drugs and Bioproducts Qingdao Marine Science and Technology Center School of Medicine and Pharmacy Ocean University of China Qingdao 266237 P. R. China; ^3^ Shanghai‐Hong Kong Joint Laboratory in Chemical Synthesis Shanghai Institute of Organic Chemistry University of Chinese Academy of Sciences Chinese Academy of Sciences Shanghai 200032 P. R. China; ^4^ Current address: School of Environmental and Chemical Engineering Wuyi University Jiangmen 529020 P.R. China; ^5^ Current address: Zhongshan Institute For Drug Discovery Shanghai Institute of Materia Medica Chinese Academy of Sciences Zhongshan 528400 P. R. China

**Keywords:** aggregation, chemical synthesis, peptides, protein

## Abstract

Hydrophobic and aggregation‐prone proteins still present obstacles for protein chemical synthesis and engineering. The chemical synthesis of Interleukin‐15 (IL‐15) is a formidable challenge due to inherent sequence hydrophobicity and severe peptide aggregation, which impedes downstream protein engineering and chemical biology studies. Here, we report the first total synthesis of IL‐15 using a versatile solubilizing strategy (RST‐2.0), which enables the preparation and ligation of aggregation‐prone segments while being readily removable during folding. Remarkably, this strategy is fully compatible with glycopeptide synthesis, allowing for the synthesis of homogeneously N79‐glycosylated IL‐15. The effectiveness of RST‐2.0 was further demonstrated through the efficient synthesis of wild‐type and azide‐labeled Interleukin‐2 (IL‐2) analogs. Moreover, the bioactivity of IL‐15 and IL‐2 analogs was validated by CTLL‐2 proliferation assays and microscale thermophoresis (MST). This work provides a *de novo* synthesis approach to elucidate the role of N‐glycosylation on IL‐15‐mediated immune regulation and lays the foundation for developing next‐generation cancer immunotherapies based on synthetic IL‐15 variants.

## Graphical abstract

A reducible solubilizing tag strategy (RST‐2.0) enables the synthesis, purification and ligation of difficult peptides while being readily removable just during folding, which overcomes the common challenges from protein hydrophobicity and aggregation. With this method, we achieved the first total synthesis of native and N‐glycosylated IL‐15 as well as the facile synthesis of native and azide‐labeled IL‐2.

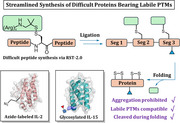

## 1 Introduction

Immunotherapy has emerged as a transformative and potent strategy for cancer treatment; however, novel therapeutic approaches are still urgently needed to address existing challenges such as unpredictable patient responses and tumor resistance. Interleukin‐15 (IL‐15) is a cytokine that plays an important role in modulating innate and adaptive immune responses. It was discovered in 1994 as a T cell growth factor interacting with interleukin‐2 (IL‐2) receptor β and γ_c_ subunit (IL‐2R_β,γc_), along with a specific receptor α subunit (IL‐15R_α_) [1, 2]. Interestingly, IL‐15 and IL‐2 have many structural similarities, including four‐helix bundle and similar geometry in their heterotetrameric signaling complex, despite low sequence identity (19%) [3]. Although they share some biological functions (*e.g*., both promoting the activation and proliferation of cytotoxic T cells and natural killer (NK) cells), they sometimes manifest opposite effects on immune responses under specific circumstances due to the engagement of specific receptor α subunits, distinct modes of action, locations, and so on [4, 5]. Over the past three decades, the potential of IL‐2‐ or IL‐15‐based immunotherapy has kindled the interest of researchers and pharmaceutical companies [6, 7].

High‐dose IL‐2 (Proleukin) was the first cancer immunotherapy for metastatic renal cell carcinoma approved by the Food and Drug Administration (FDA, U.S.A.) in 1992. However, this IL‐2 therapy requires high‐dose injection that can cause adverse effects, because IL‐2 not only activates natural killer cells and effector T cells but also activates CD4^+^ regulatory T cells (T_reg_), thereby downregulating immune responses [8, 9]. Consequently, many efforts have been made to abolish IL‐2R_α_ engagement which drives T_reg_ activation [10]. In contrast, IL‐15 does not promote T_reg_ activation or induce activation‐induced cell death to suppress immune responses [11]. Beyond promoting the activation of NK cells and cytotoxic T cells, IL‐15 is also critical for the long‐term survival and expansion of CD8+ memory T cells, which may provide persistent anti‐tumor immunity. Therefore, IL‐15‐based therapy has emerged as a promising approach for cancer immunotherapy [12, 13]. However, current strategies for IL‐15 engineering focus on biochemical methods [13], and it has been challenging to produce structurally defined IL‐15 analogs with atomic precision. Furthermore, the role of IL‐15 post‐translational modifications (PTMs) remains largely unexplored from a chemical biology perspective, due in part to the lack of synthetic access to homogeneously modified proteins.

IL‐15 is a glycoprotein containing three putative N‐glycosylation sites and two disulfide bonds [14, 15]. N‐glycosylation is a well‐known PTM in eukaryotic proteins, a way of nature to access higher structural diversity and complexity [16], and is often associated with human diseases [17, 18]. It has been proved that glycosylation patterns (such as core‐fucosylation, sialylation) and glycosylation degree have a significant influence on biological activity of proteins [19, 20, 21, 22, 23]. Therefore, a detailed investigation on how natural glycosylation regulates the biological function of IL‐15 is essential for the design of engineered IL‐15 variants. A major obstacle to such studies is that N‐glycoproteins are typically produced as heterogeneous mixtures. This heterogeneity creates combined effects on biological activity, making it difficult to delineate the function of glycans at specific sites. Furthermore, for therapeutic proteins like cytokines and antibodies, heterogeneous glycoforms present a significant challenge to ensuring consistent biological activity and clinical efficacy, underscoring the critical need to investigate N‐glycan functions [24].

We thereby decided to develop a chemical synthesis approach for investigating N‐glycan functions of IL‐15 and developing a novel IL‐15‐based immunotherapy. However, our initial study indicated that IL‐15 is a formidable synthetic target, with all peptide segments suffering from severe truncation, displaying high aggregation tendency, poor solubility and/or low reactivity. Consequently, total synthesis of IL‐15 has not been reported yet. In this work, we achieved the first total synthesis of both native and N^79^‐glycosylated IL‐15 using a versatile solubilizing strategy. We initially employed tunable backbone modification (TBM) [25] to inhibit peptide aggregation. However, after TBM removal, the linear IL‐15 exhibited extremely poor solubility, preventing effective purification and folding. To overcome the issues of aggregation and poor solubility, we established the second‐generation reducible solubilizing tag [26] (RST‐2.0) strategy based on two novel cysteine building blocks (**Cys‐N** and **Cys‐Sc**). This method features operational simplicity, versatility and synthetic efficiency. A key feature is that the RSTs were retained throughout the synthesis of IL‐15 and simultaneously cleaved in the folding step. This design significantly improves synthetic efficiency by preventing tedious post‐ligation manipulation and purification. We further demonstrated the broad applicability of RST‐2.0 for difficult protein synthesis by successfully preparing both wild‐type and azide‐labeled IL‐2. Overall, this work provides a robust synthetic platform for revealing the structure–function relationships of IL‐15 N‐glycoproteins, which will facilitate subsequent chemical biology studies and advance the development of cytokine‐based immunotherapies.

## 2 Results and Discussion

### 2.1 First Synthetic Attempt of Non‐glycosylated Interleukin‐15

**FIGURE 1:**
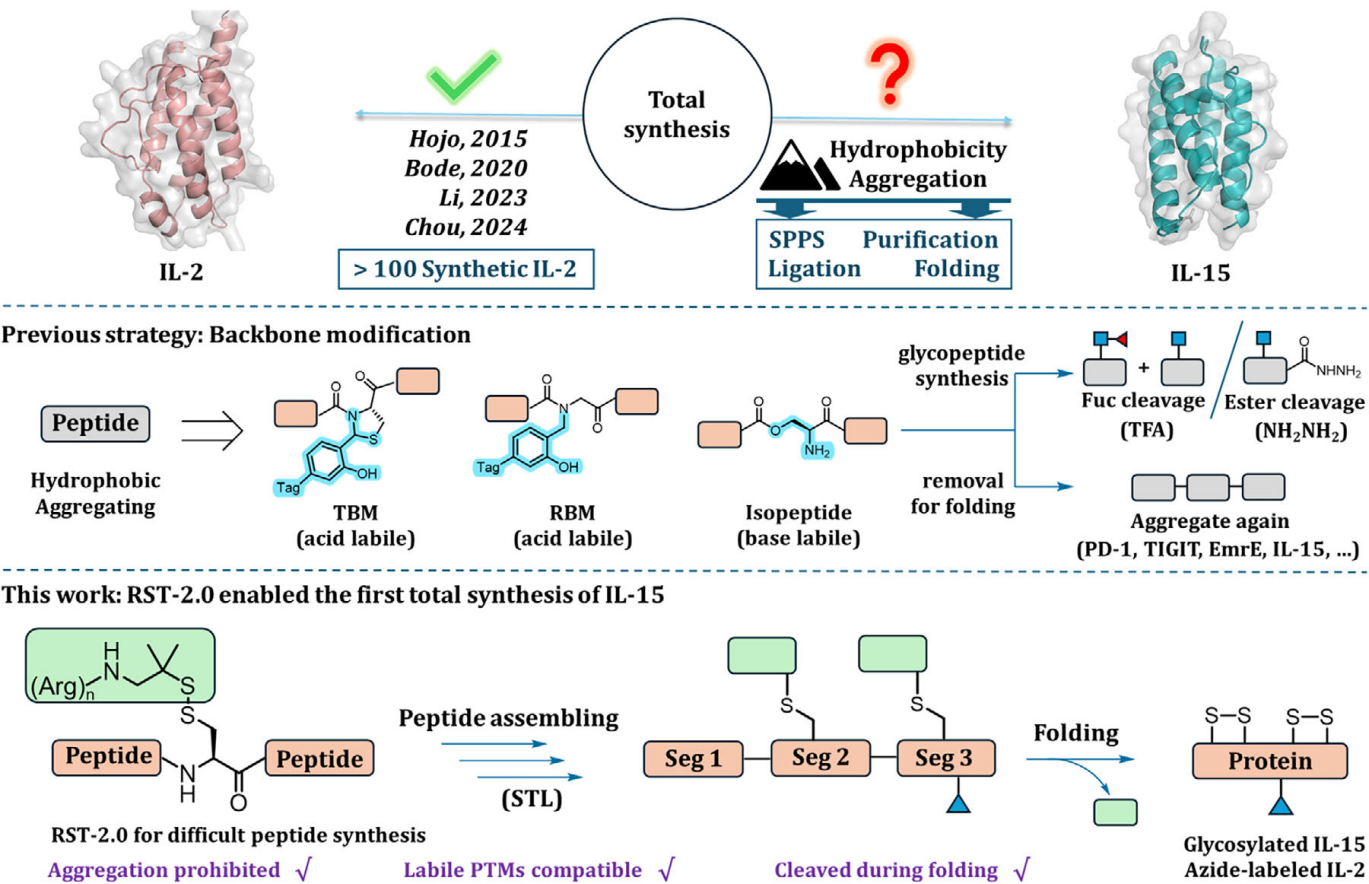
Limitations of backbone modification strategy for difficult protein synthesis and streamlined synthesis of glycosylated IL‐15 and azide‐labeled IL‐2 via the second‐generation reducible solubilizing tag strategy (RST‐2.0). TBM: tunable backbone modification; RBM: removable backbone modification; Fuc: fucose; STL: Ser/Thr ligation.

Over the past few years, chemical protein synthesis has been greatly advanced by the development of synthetic methodologies for peptides and proteins, covering resins [27], protecting groups [28, 29, 30, 31, 32, 33, 34], backbone modifications [25, 35], solubilizing tags [26, 36, 37, 38, 39, 40], peptide ligation [41, 42, 43, 44, 45, 46], post‐ligation manipulation (*e.g*., deprotection [37, 47], desulfurization [48, 49, 50]) and folding [51, 52, 53, 54]. Despite these achievements, robust strategies are still very rare for difficult peptide and protein targets which cannot be prepared straightforwardly by typical solid phase peptide synthesis (SPPS) and peptide ligation, especially those bearing delicate PTMs. During SPPS, backbone modifications, including pseudo‐prolines at Ser/Thr [55], N‐(2‐hydroxy‐4‐methoxybenzyl) (HMB) [56], and isopeptides [57], have been discovered as effective peptide aggregation disruptors for difficult peptide synthesis. However, these tools are still not adequate for overcoming peptide and protein aggregation problems. Importantly, removal of backbone modification results in decreasing or losing solubility of aggregation‐prone linear proteins (*e.g*., EmrE [35], TIGIT [58], PD‐1 [59] and IL‐15). In addition, many backbone modifications require highly acidic conditions to be removed (*e.g*., TBM, RBM) or cannot tolerate strong base (isopeptides), thus they are not compatible with the synthesis of some glycopeptides. (Figure 1)

**SCHEME 1:**
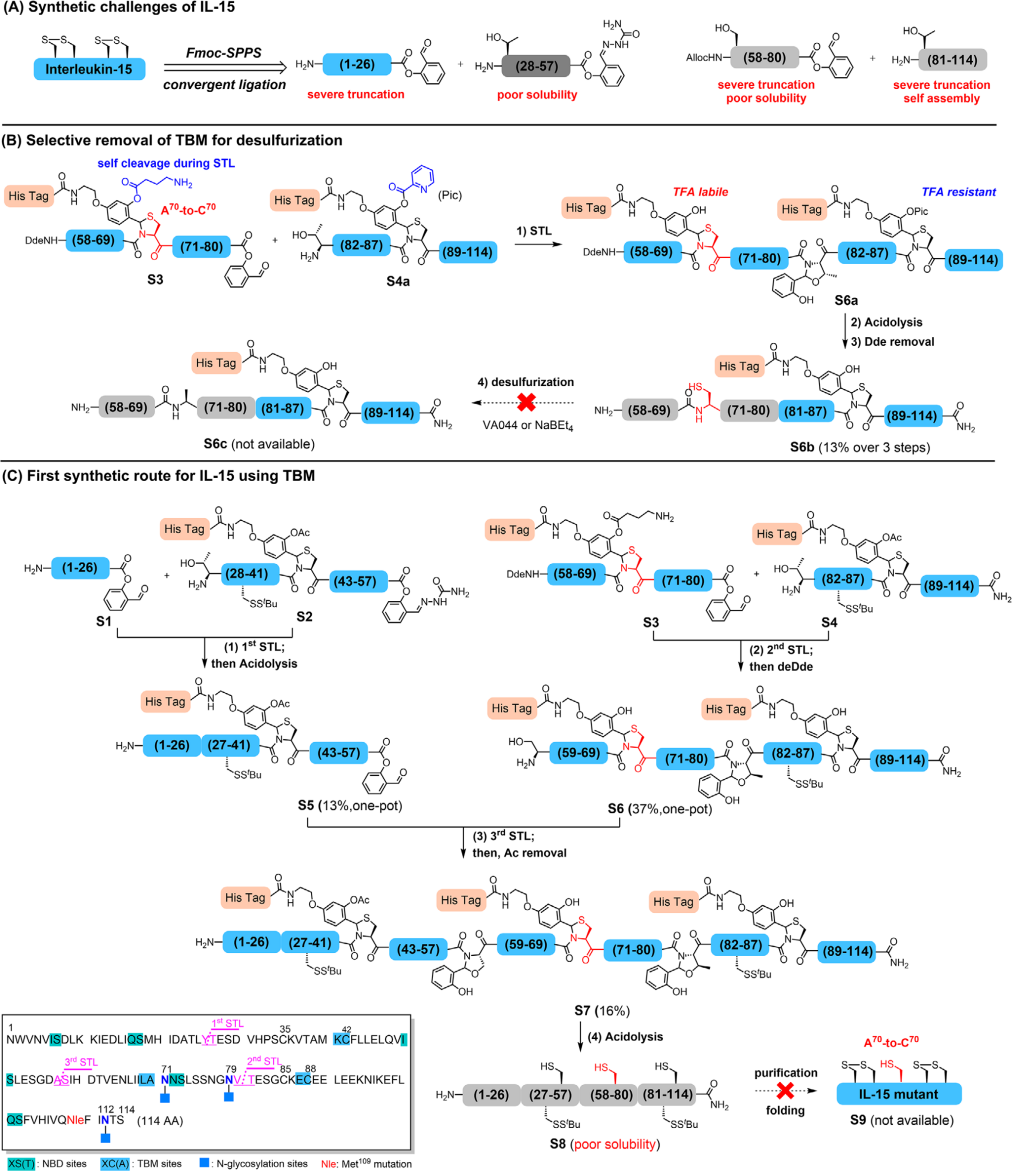
(A) Synthetic challenges of IL‐15 peptide segments; (B) Selective removal of TBM for desulfurization. Conditions: (1) STL: Py/HOAc 1:1, 2 h; (2) Acidolysis, TFA/H_2_O/AcAc = 95:2.5:2.5, 2 h; (3) Dde removal: 5% hydrazine in 6 M GnHCl, 30 min; (4) Desulfurization: 6 M GnHCl, 0.2 M TCEP, VA044, *^t^* BuSH, pH 6.5 or 6 M GnHCl, 0.2 M TCEP, NaBEt_4_, pH 4.5, 16 h; (C) Initial synthetic route for non‐glycosylated IL‐15 via tunable backbone modification. Conditions: (1) First STL: Py/HOAc 1:1, 2 h; One‐pot acidolysis, TFA/H_2_O/AcAc = 95:2.5:2.5, 2 h; (2) Second STL, Py/HOAc 1:1, 16 h; One‐pot Dde removal: 5% hydrazine in 6 M GnHCl, 30 min; (3) Third STL, Collidine/HOAc 1:1 (20% DMSO), 16 h; then, Ac removal: 6 M GnHCl, pH 8, 3 h; (4) Acidolysis: TFA/H_2_O/EDT = 95:2.5:2.5, 2 h.

In our preliminary synthetic studies, peptide segments of IL‐15 disconnected at Ser/Thr ligation (STL) sites were all difficult to access via conventional SPPS, purification and ligation workflows, either suffering from severe truncation, aspartimide formation, or displaying high aggregation tendency, poor solubility and low reactivity. Specifically, IL‐15 sequence was divided into four segments for convergent synthesis using STL (Scheme 1A), but none of them were obtained via typical Fmoc‐SPPS or microwave‐assisted automatic synthesis. To our delight, peptide IL‐15‐(1–26) (S1) was successfully prepared after using *N,O*‐benzylidene acetal dipeptides [60] (NBDs) to inhibit on‐resin peptide aggregation and consequent truncation. In contrast, peptide IL‐15‐(27–56) and IL‐15‐(58‐79) were still not synthetically accessible with the help of NBDs because of extremely poor solubility in 6 M GnHCl buffer or ACN/H_2_O, especially the former one which was hardly detected in UPLC‐MS during peptide elongation. Although IL‐15‐(81‐114) could be synthesized by NBD‐assisted SPPS, it would assemble and precipitate very fast in 50% ACN due to ionic and hydrophobic interactions. These challenges necessitated the introduction of aggregation disruptors and solubilizing tags to achieve IL‐15 total synthesis.

Tunable backbone modification (TBM) proved to be an effective strategy to disrupt peptide aggregation and introduce solubilizing tags for the synthesis of difficult peptides and proteins [25, 61, 62]. The stability of TBM in TFA cocktails is switchable by acetyl capping or removal. Inspired by our previous success of IL‐2 total synthesis [25], we envisioned that TBM would also be helpful to address aggregation problems in the synthesis of IL‐15 segments. Indeed, with the combination of NBD and TBM strategy, peptide S2 and S4 were successfully prepared in 22% and 31% yield, respectively. As segment IL‐15‐(58‐80) had no native cysteine residue, Ala^70^ was replaced with Cys^70^ to introduce TBM, and a self‐cleavable unit was chosen to “turn off” the TBM, giving peptide S3 in 23% isolated yield. After STL between S3 and S4, the TBM at Ala^70^‐to‐Cys^70^ site was “turned on” for selective acidolysis. After Dde removal in one pot, peptide S6b was obtained in 13% overall yield over three steps for desulfurization. Unfortunately, peptide S6b tended to decompose or aggregate under desulfurization conditions using VA044 or NaBEt_4_ as radical initiator (Scheme 1B), and no product or starting material was detected in UPLC trace after overnight reaction. More importantly, we tried to perform the final STL between S5 and S6b and surprisingly found that the removal of TBM at Ala^70^‐to‐Cys^70^ site abolished the ligation reactivity of peptide S6b despite its good solubility (SI, Figure S30). Therefore, we decided to temporarily set aside the optimization of this desulfurization and focus on the synthesis of IL‐15 Ala^70^Cys mutant.

With the four peptide segments (S1, S2, S3 and S4) in hand, we performed the first STL between S1 and S2 followed by one‐pot acidolysis to regenerate the C‐terminal salicylaldehyde (SAL) ester. Notably, to protect N‐terminal tryptophan from nucleophilic addition to pyruvic acid, acetylacetone (AcAc) was used as additive instead of pyruvic acid in TFA/H_2_O cocktail for regenerating the C‐terminal SAL ester [62]. Meanwhile, peptide S6 was also synthesized by STL between S3 and S4 and one‐pot Dde and acetyl deprotection. Subsequently, we continued to perform the final STL between S5 and S6, which afforded peptide S7 in 16% isolated yield. After removing the Ac on *N,S‐*benzylidene thioacetal, linear protein S8 was generated by acidolysis. Unfortunately, when diluting the protein solution in TFA with 50% ACN/H_2_O, all S8 precipitated from the acidic solution. Once forming protein pellet, S8 was nearly insoluble in 8 M GnHCl or 50% ACN/H_2_O after heating or shaking overnight (SI, Figure S18), which made the final purification and folding impossible. It is conceivable that IL‐15 exhibits overall negative charge (18 Asp and Glu, but only 7 Arg and Lys), so the deprotonation and solvation of Asp and Glu residues could be inhibited in acidic solution without denaturant, thus the self‐assembly and aggregation of IL‐15 could be promoted via intermolecular hydrophobic interaction and hydrogen bonding. One‐pot final STL/acidolysis protocol produced a mixture of peptide aggregates, allowing S8 to be slightly soluble in 6 M GnHCl buffer and detectable in UPLC‐MS (SI, Figure S19). However, S8 (linear IL‐15) was hardly eluted during HPLC purification because it tended to aggregate in C4 column.

In this context, we realized that protein aggregation, either in solution or during column purification, has been a common obstacle for the synthesis of hydrophobic proteins, including membrane‐bound proteins (*e.g*., EmrE [35]), immunoglobulin‐like proteins (*e.g*., TIGIT [58], PD‐1 [59]) and amyloid‐like proteins. Currently, most of the solubilizing strategies require post‐ligation deprotection to regenerate native amide bonds or peptide side chains before protein refolding, resulting in the peptide becoming highly hydrophobic again. On the other hand, the removal of backbone modifications usually require harsh conditions (strong acids), which may not be compatible with delicate glycan moieties on glycoproteins such as fucose and sialic acid [63]. Therefore, current synthetic methodologies are rarely suitable for hydrophobic glycoproteins, and a robust solubilizing strategy for difficult (glyco)peptide synthesis is still urgently needed. These challenges prompted us to develop the second generation of reducible solubilizing tag strategy (RST‐2.0) for the synthesis and purification of difficult peptides and proteins. (Figure 1)

### 2.2 Development of RST‐2.0 for the Synthesis of Hydrophobic and Aggregating Peptides

**SCHEME 2:**
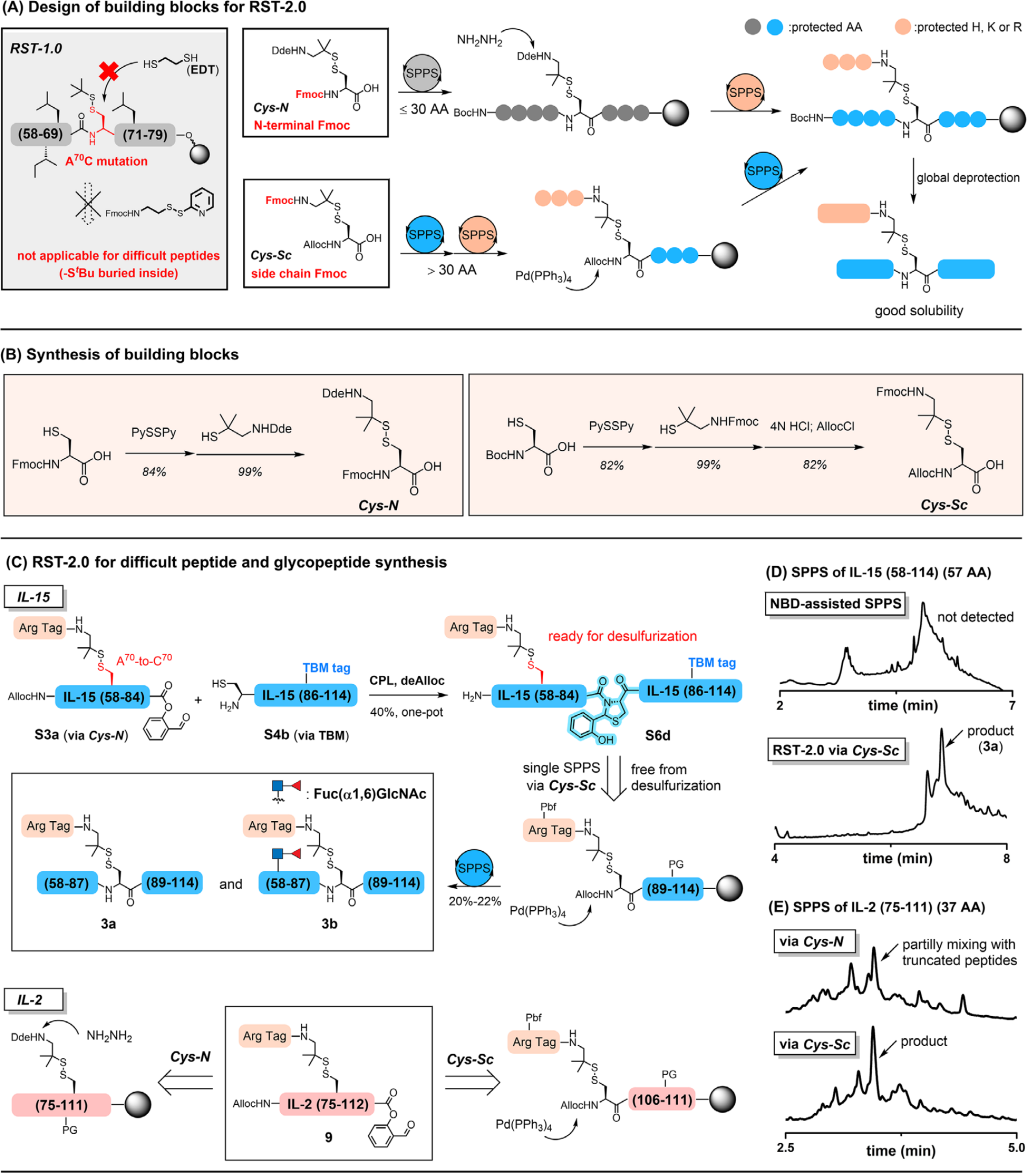
(A) Design of Cys‐N and Cys‐Sc building blocks to develop RST‐2.0 for difficult peptide synthesis; (B) Facile synthesis of Cys‐N and Cys‐Sc; (C) Application of RST‐2.0 to difficult peptide segments from IL‐15 and IL‐2; (D) Spectra of crude IL‐15 peptide from NBD‐assisted SPPS or RST‐2.0 by Cys‐Sc (E) Spectra of crude IL‐2 peptide via RST‐2.0 by Cys‐N or Cys‐Sc.

It is well‐known that rapid disulfide exchange is often involved in protein refolding. We proposed that RSTs could be removable in the folding buffer and thus facilitate the synthesis of aggregating proteins without needing extra deprotection and purification steps. Previously, a preliminary protocol for reducible solubilizing tags (RST‐1.0) strategy had been developed by our group [26], but it hardly worked on difficult peptides, such as IL‐2‐(75‐111) and (112‐133) [25]. This is because the –S*^t^* Bu protecting group on Cys cannot be removed on resin, while –SMe is not stable enough during SPPS (Scheme 2A). Meanwhile, simple disulfide bonds cannot survive under very basic conditions (*e.g*., hydrazine, NaOMe/MeOH) during acetyl removal of glycopeptides (SI, Figure S6).

To exploit RST‐2.0 for difficult (glyco)peptide synthesis, we designed two novel cysteine building blocks which introduce solubilizing tags in different peptide elongation orders (Scheme 2A). For **Cys‐N** featuring an N‐terminal Fmoc, the whole target peptide sequence was elongated first. After removal of Dde protecting group on disulfide linker via hydrazine treatment, a solubilizing tag could be elongated via simple SPPS (Scheme S1, method A). Compared with RST‐1.0 on‐resin manipulation protocol, this synthetic building block is more convenient and reliable for installation of solubilizing tags. This method is suitable for the preparation of hydrophobic peptides shorter than 30 amino acid residues (AA) (peptide **2**, **10** and S3a). Remarkably, the combination of TBM with this strategy would be beneficial to selectively recover alanine by desulfurization.

For **Cys‐Sc** featuring Fmoc‐protected amine on the disulfide side chain, the elongation of solubilizing tag is performed before main chain elongation during Fmoc‐SPPS (Scheme S1, method B), followed by Alloc removal and elongation of the rest of target sequence. In this manner, solubilizing tag can serve as an aggregation disruptor to inhibit, to some extent, on‐resin aggregation and truncation of difficult peptides [39]. In addition, as prior installation of polyarginine tag ensures good solubility, the peptide elongation could be monitored by LC‐MS, thus side reactions (*e.g*., aspartimide formation, truncation) could be noticed and carefully prevented. This strategy enables the facile synthesis of long difficult peptides (>50 AA) which are otherwise inaccessible. For example, segment IL‐15‐(58‐114) was hard to obtain via conventional SPPS due to severe side reaction and aggregation. In contrast, **Cys‐Sc** allows prior introduction of polyarginine tag for inhibiting peptide aggregation on resin, generating peptide **3a (**or **3b**) as a sharp peak in UPLC trace (Scheme 2D). In addition, this disulfide linker has higher steric hindrance than RST‐1.0 version to tolerate hydrazine treatment during Ac removal of glycopeptide **3b** (Scheme 2C). Moreover, the crude SPPS spectra of difficult segment IL‐2‐(75‐112) further showcased the advantage of **Cys‐Sc**. When using **Cys‐N** for RST installation, the desired product partially overlapped with truncated products, while **Cys‐Sc** provided cleaner crude product with good resolution from the impurities (Scheme 2E). These results highlighted the capacity of RST‐2.0 (especially via **Cys‐Sc**) for efficient synthesis of aggregating peptides. Notably, **Cys‐N** and **Cys‐Sc** could be easily prepared on a multigram scale with two or three simple steps in high overall yields (84%, 66%, respectively) (Scheme 2B).

### 2.3 Synthesis of Wild‐type and N79‐glycosylated Interleukin‐15 Using RST‐2.0 Strategy

With two cysteine building blocks in hand, we were able to overcome the solubility challenges of difficult peptides without modifying peptide backbone. In the beginning, **Cys‐N** was employed for the synthesis of peptide **2** by replacing TBM with RST strategy on the same site (Cys^42^). Not surprisingly, when using His_8_ tag as RST, the yield of peptide **2** decreased to 4.7% due to gel‐formation, lower than TBM counterpart **S2** (22%). Later, we used Arg_8_ tag for the synthesis of **2** and the yield was improved to 22%, highlighting the capacity of polyarginine tags for inhibiting peptide aggregation and gel‐formation. For the synthesis of segment IL‐15‐(58‐80), however, it was no longer feasible to mutate Ala^70^Cys followed by desulfurization, as all native cysteines were protected by disulfide. To address this problem, strategic combination of RST and TBM was employed to enable site‐specific desulfurization after ligation. Peptide S3a and S4a were prepared by RST‐2.0 and TBM, respectively. After that, Cys/Pen ligation (CPL) between S3a and S4a provided peptide S6d which was ready for desulfurization, as Cys^85^ was protected by *N,S*‐benzylidene thioacetal after ligation (Scheme 2D). However, desulfurization at Ala^70^‐to‐Cys^70^ site was still unsuccessful. In this context, we designed and synthesized **Cys‐Sc** for long aggregating peptide synthesis as it was discussed above (Scheme 2B). To our delight, when using **Cys‐Sc** for RST‐2.0, segment IL‐15‐(58‐80) and (81‐114) were successfully combined in one single SPPS without Ala‐to‐Cys substitution and gave peptide **3a** in 20% isolated yield. This result highlights the advantage of **Cys‐Sc** to enable the synthesis of long difficult peptides and simplify the workflow of chemical protein synthesis. Remarkably, glycopeptide **3b** was also obtained using the same strategy with 22% isolated yield after global deprotection and acetyl deprotection (hydrazine treatment) in one pot.

**SCHEME 3:**
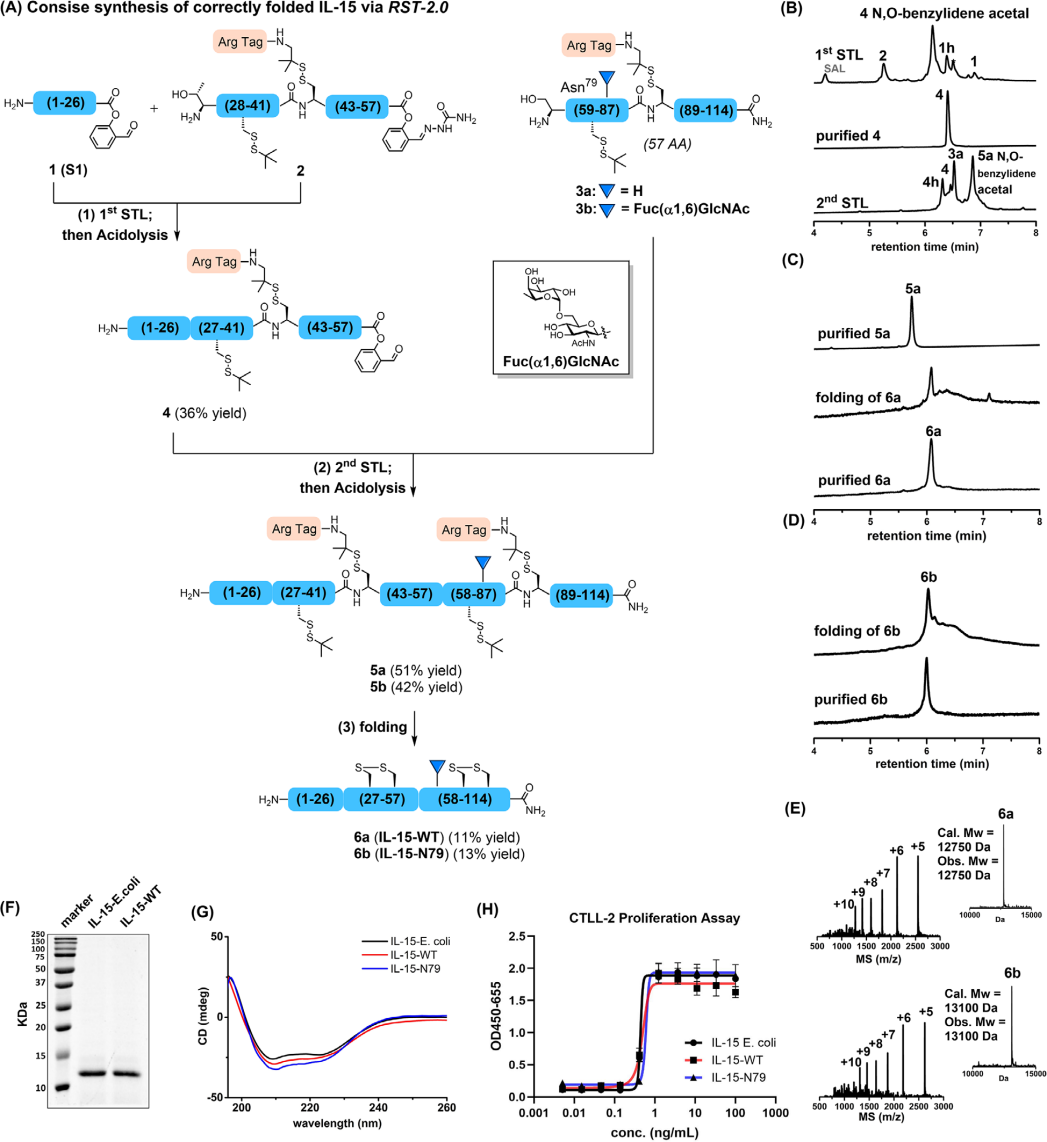
(A) Synthetic route for wild‐type and N79‐glycosylated IL‐15 via RST‐2.0. Conditions: First STL, Py/HOAc 1:1, 2 h; Acidolysis, TFA/H_2_O/AcAc = 95:2.5:2.5, 2 h; Second STL, Py/HOAc 1:1, 16 h; Acidolysis, TFA/H_2_O/TIPS = 95:2.5:2.5, 30 min (if bearing glycan, TFA/ACN/H_2_O = 20:40:40, 3 h); Folding: a) Denaturation: 6 M GnHCl, 0.1 M Tris, 2 mM TCEP, pH 8.0, 2 h; b) Dialysis against folding buffer: 1 M GnHCl, 0.1 M Tris, 10 mM Cysteamine, 2.5 mM Cystamine, pH 8.5, 4°C 24 h, then, 1 M GnHCl, 0.05 M Tris, 1 mM GSH, 0.5 mM GSSG, pH 8.0, 4°C 12 h; (B) UPLC spectra for ligations, **1** and **4** **h** are SAL ester hydrolysis side products; (C) UPLC trace for **IL‐15‐WT** folding; (D) UPLC trace for **IL‐15‐N79** folding; (E) ESI‐MS and deconvoluted MS; (F) SDS‐PAGE of *E. coli*‐expressed IL‐15 and folded **IL‐15‐WT**; (G) Circular dichroism of IL‐15 proteins; (H) CTLL‐2 cell proliferation assay of IL‐15 proteins.

With three peptide segments (**1**, **2** and **3a**/**3b**) in hand, we continued to assemble the full‐length IL‐15 with only two STL. To our delight, the first STL/acidolysis provided peptide **4** in 36% isolated yield (for TBM route, 13%), suggesting that disulfide‐linked polyarginine tag could be a replacement for TBM to disrupt peptide secondary structure. Moreover, the second STL/acidolysis afforded peptide **5a** in 51% isolated yield (for TBM with His_8_ tag, 16%), further underscoring the capacity of RST‐2.0 for improving ligation efficiency. The same synthetic route was also adopted for the synthesis of the glycosylated IL‐15 bearing a disaccharide at Asn^79^ via replacing **3a** with **3b** (Scheme 3A). It is worth mentioning that RSTs could be simultaneously removed during protein folding, which overcomes the problem of linear IL‐15 aggregation in C4 column and avoids tedious deprotection‐purification steps in the meantime.

We then focused on the folding condition screening. We envision that the key to IL‐15 folding is to ensure rapid disulfide exchange by controlling pH and choosing suitable redox pair, so that folding intermediates can be stabilized by native disulfide and formation of protein aggregates can be reduced. Meanwhile, the folding of **5a** required prior removal of RSTs in denaturation buffer; otherwise, RSTs could not be fully cleaved during folding. Initially, we attempted to perform RSTs cleavage and protein folding concurrently, yet RST removal was not efficient under these conditions, generating an (IL‐15 + Arg_8_ tag + S*^t^* Bu) unfolded side product (SI, Figure S44). Then, we tried the reported folding conditions for IL‐2 [34] considering the structural similarity between IL‐2 and IL‐15. Firstly, **5a** was dissolved in the denaturation buffer containing 6 M GnHCl and 30 mM GSH and then incubated the solution at 50°C for 2 h to break all secondary structure and remove all disulfides. The reaction generated two peaks in UPLC spectrum likely because of the formation of aspartimide side products resulting from Asn^77^ deamidation [64]. Therefore, the temperature was controlled at 37°C with TCEP as reductant for the reduction/denaturation step, which generated linear IL‐15 as a single peak in UPLC spectrum. Subsequently, linear IL‐15 solution in denaturation buffer was diluted by adding GnHCl‐free buffer to allow renaturation, with GSH/GSSG as redox pair. Unfortunately, this dilution protocol for IL‐2 refolding was not successful in the case of IL‐15, generating many misfolded products and protein aggregate. Meanwhile, reported conditions [65, 66] for recombinant IL‐15 refolding using GSH/GSSG as redox pair also gave negative results (data not shown). We thereby tried dialysis methods using GSH/GSSG or 2‐mercaptoethanol/2,2'‐dithiodiethanol as redox pairs, but the folding still produced many inseparable protein aggregates probably due to slow disulfide exchange. Fortunately, when using cysteamine/cystamine as redox pair, the folding generated a sharp peak in UPLC spectrum, and ESI‐MS distribution pattern suggested the formation of disulfide cross‐linked globular protein. Subsequently, the folded **IL‐15‐WT** (**6a**) was purified by C4 column in HPLC (11% yield) or by size exclusion column to reduce the risk of denaturation from organic solvent (6% yield). For further characterization, SDS‐PAGE (Scheme 3F) indicated correct molecular weight, high‐resolution mass spectrometry (HRMS) (SI, Figure S50) showed formation of two disulfide bonds, circular dichroism (CD) indicated correct helical structure, and the bioactivity of synthetic IL‐15 proteins in CTLL‐2 proliferation assay was comparable with *E. coli*‐expressed IL‐15 (Scheme 3F–H). These results strongly supported the successful chemical synthesis of bioactive IL‐15. With the same workflow, glycosylated IL‐15 (**6b**) was also synthesized in 13% folding yield. Overall, RST‐2.0 strategy not only facilitated the synthesis of difficult (glyco)peptides but also improved the ligation efficiency without the requirement of post‐ligation deprotection and purification.

It is worth noting that the disulfide bond pairing of human IL‐15 suggested by UniProt (P40933) and PDB (2Z3Q) was wrong. Current opinions on IL‐15 disulfide bond pairing were based on homology modeling with IL‐2 [1, 15, 67, 68, 69, 70], although their sequence similarity is only 19%. Actually, experimental evidence had been provided for IL‐15 disulfide bond pairing (Cys^35^‐Cys^42^, Cys^85^‐Cys^88^) using LC‐MS/MS analysis [71], but attracted little attention during last decade. This circumstance suggests that disulfide bond analysis on disulfide‐rich proteins is often overlooked in structural biology. Consequently, the wrong disulfide‐bonded IL‐15 had been used as structural model for IL‐15 engineering via disulfide bond remodeling on IL‐15/IL‐15R_α_ binding interface [70], which might produce puzzling results. On the other hand, prior formation of disulfide bond before folding (*e.g*., insulin [40]) or converting Cys to Ser for reducing wrong cysteine pairing (*e.g*., PD‐1 [59], IL‐2 [72],) are both widely used strategy to overcome the refolding challenge of cysteine‐rich proteins. Thus, wrong information about disulfide pairing for chemical protein synthesis would likely lead to inactive synthetic proteins. Those unwanted situations implied the necessity of confirming cysteine pairing before total synthesis.

### 2.4 Synthesis of IL‐2 Analogues Using RST‐2.0 Strategy

**SCHEME 4:**
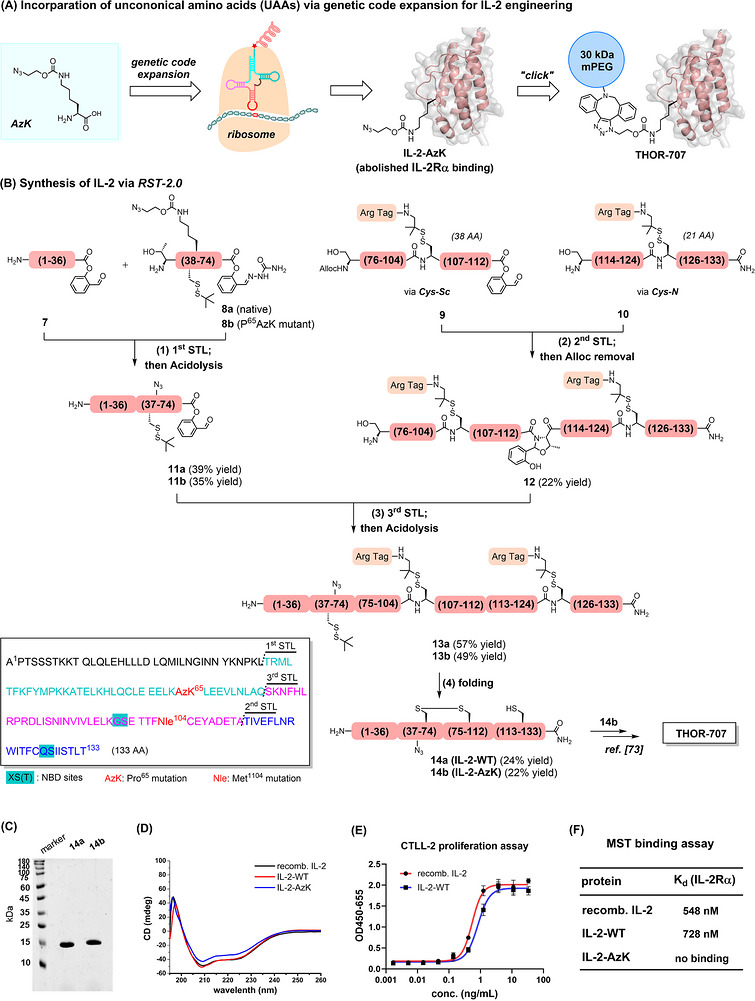
(A) Preparation of PEGylated IL‐2 variant THOR‐707 via genetic code expansion; (B) Synthetic route for wild‐type and azide‐labeled IL‐2 via RST‐2.0 strategy. Conditions: (1) First STL, Py/HOAc 1:1, 2 h; Acidolysis, TFA/H_2_O/pyruvic acid = 95:2.5:2.5, 2 h; (2) Second STL, Py/HOAc 1:1 (20% DMSO), 16 h; One‐pot Alloc removal: Pd(PPh_3_)_4_, 1,3‐dimethyl barbituric acid, HOAc, 1 h; (3) Third STL, Py/HOAc 1:1 (20% DMSO), 16 h; (4) Folding: a) Denaturation: 6 M GnHCl, 0.1 M Tris, 30 mM GSH, pH 8.0, 2 h; b) Dialysis against folding buffer: 2 M GnHCl, 0.1 M Tris, 10 mM GSH, 1 mM GSSG, pH 8.0, 4°C 24 h, then PBS, 4°C, four times for 12 h; (C) SDS‐PAGE of **IL‐2‐WT** and **IL‐2‐AzK** (15); (D) Circular dichroism of IL‐2 proteins; (E) CTLL‐2 cell proliferation assay of IL‐2 proteins; (F) Microscale thermophoresis for measuring binding affinity to IL‐2R_α_.

With the success of RST‐2.0 for the total synthesis of IL‐15, we further demonstrated its applicability for the synthesis of another difficult protein, IL‐2. Over the past three decades, many efforts have been made to improve the therapeutic outcomes of IL‐2‐based immunotherapy, focusing on abolishing binding ability to IL‐2Rα to reduce toxicity and bioconjugation to prolong serum half‐life. For instance, THOR‐707 is a promising candidate for cancer treatment, which bears a 30 kDa PEG with binding bias to IL‐2R_β,γ_ [73]. The preparation of THOR‐707 relies on a semi‐synthetic organism using genetic code expansion techniques and DBCO‐based copper‐free click chemistry (Scheme 4A). Another approach to producing this kind of defined IL‐2 variants is chemical protein synthesis. It was claimed that *Bright Peak Therapeutics* has achieved the preparation of more than one hundred IL‐2 analogues by chemical synthesis using KAHA ligation [74], which showcases the potential of chemical methods in modification and industrial production of therapeutic proteins.

Our previous synthetic route for IL‐2 using TBM strategy required several deprotection (or desulfurization) and purification steps. After final ligation, TBMs were all removed to regenerate native amide bonds, generating hydrophobic linear IL‐2 with low isolated yields (19%) from C4‐HPLC purification despite good ligation efficiency [25]. To further showcase the potential of RST‐2.0 for difficult protein synthesis, we envisioned that RST‐2.0 could be an optimal synthetic strategy for this challenging target. Thus, we continued to synthesize native IL‐2 (**IL‐2‐WT**) and Pro^65^AzK mutant (**IL‐2‐AzK**) purely using STL for peptide assembly. Specifically, peptide **7**, **8a**, and **11a** were prepared using reported protocol. Fmoc‐AzK‐OH was incorporated into peptide **8b** via typical Fmoc‐SPPS, while **11b** was synthesized by STL/acidolysis between **7** and **8b** with 35% isolated yield. RST‐2.0 was employed for the synthesis of highly hydrophobic and aggregating peptides, using **Cys‐Sc** for the preparation of **9** (15% yield) and **Cys‐N** for the preparation of **10** (24% yield). Afterwards, peptide **12** was successfully obtained after second STL and Alloc removal in a one‐pot manner. Subsequently, linear IL‐2 (**13a** or **13b**) could be efficiently synthesized by final STL/acidolysis between peptide **11a**/**11b** and **12** in good yield (57% and 49%, respectively), with all cysteines masked by RSTs or –S*^t^* Bu groups. The purified RST‐containing proteins were directly folded using the reported IL‐2 folding protocol [34]. We also tried to perform RST cleavage and protein folding concurrently, which provided similar results to the stepwise reduction/folding protocol. Yet the above two protocols still required HPLC purification to remove misfolded proteins (SI, Figure S79).

Finally, IL‐2 proteins were directly folded by a dialysis protocol without needing extra HPLC purification. To be specific, **13a**/**13b** were dissolved in 6 M GnHCl/30 mM GSH denaturation buffer, in which all RSTs were detached, and all secondary structure was broken to generate linear IL‐2. Then, the resulting protein solution was transferred to a dialysis tube and dialyzed against folding buffer and later PBS. Remarkably, unlike the widely used dilution strategy, this dialysis protocol provided pure folded IL‐2 without any column purification, thus avoiding the risk of protein deactivation in ACN/H_2_O during HPLC purification. After ultrafiltration, folded **IL‐2‐WT** (**14a**) and **IL‐2‐AzK** (**14b**) were obtained in PBS solution and ready for characterization and biological application. The synthetic IL‐2 analogues were subjected to SDS‐PAGE and CD test, indicating correct molecular weight and α‐helical structure (Scheme 4C, D). CTLL‐2 cell proliferation assay and microscale thermophoresis (MST) assay demonstrated that the biological activity of synthetic **IL‐2‐WT** closely resembled the recombinant one, with slight differences probably coming from PTMs on recombinant IL‐2 (Scheme 4E, F). Notably, **IL‐2‐AzK**, designed to abolish receptor α binding but retain binding to other receptor subunits, showed no binding affinity to IL‐2R_α_ based on MST results, which was consistent with the literature [73]. Therefore, our chemical synthesis approach could be applied to the formal synthesis of THOR‐707 and the development of an enhanced version. These results fully supported the successful application of RST‐2.0 to the chemical synthesis of bioactive IL‐2 variants, with high synthetic efficiency and operational convenience.

## 3 Conclusion

In summary, we have accomplished the chemical synthesis of native and N79‐glycosylated IL‐15 for downstream chemical biology study and protein engineering. A versatile reducible solubilizing strategy (RST‐2.0) has been exploited to overcome the synthetic challenge from peptide and protein aggregation. The preparation of hydrophobic and aggregating peptide segments from IL‐15 and IL‐2 validated the applicability of RST‐2.0 in difficult peptide synthesis as well as glycopeptide synthesis. Moreover, the potential of RST‐2.0 as a general strategy for overcoming the aggregation problem of difficult proteins had been demonstrated by the synthesis of bioactive IL‐15 and IL‐2 analogues. This strategy leverages the compatibility of RSTs removal in folding step, so that solubilizing tags could be retained throughout the synthesis to ensure good solubility and easy handling, then be removed just during folding. It not only overcomes the aggregating problem of linear proteins but also prevents complicated post‐ligation manipulation and purification. This work provides robust synthetic approaches for defined IL‐15 and IL‐2 variants and highlights the power of chemical protein synthesis in flexible modification of challenging therapeutic proteins. We envisage that the following structure‐function relationship study on N‐glycosylated IL‐15 would offer useful guidance for the design of next‐generation cancer immunotherapy based on synthetic IL‐15 variants. Such kinds of studies are ongoing in our group and will be reported in due course.

## Author Contributions

**Jingwen Zeng**: methodology, data curation, investigation, validation, writing – original draft. **Haiyan Zhou**: validation, data curation. **Wang Xia**: validation, data curation. **Hongxiang Wu**: validation, data curation. **Xuechen Li**: conceptualization, supervision, resources, funding acquisition, writing – review and editing.

## Conflicts of Interest

The authors declare no conflicts of interest

## Supporting information




**Supporting File 1**: The authors have cited additional references within the Supporting Information [75].

## Data Availability

The data that supports the findings of this study are available in the supplementary material of this article
