# Supplementary material for: Total Chemical Synthesis of Interleukin‐15 and Interleukin‐2: Taming Protein Hydrophobicity and Aggregation by a Versatile Solubilizing Strategy
Source: Angew Chem Int Ed Engl. 2026 May 25;65(30):e2586132. doi: 10.1002/anie.2586132 (PMC13383028; doi:10.1002/anie.2586132)
Supplement: Supplementary file 1 — Supporting File 1: The authors have cited additional references within the Supporting Information [75]. [file ANIE-65-e2586132-s001.pdf]

Supporting information

for

Total Chemical Synthesis of Interleukin-15 and Interleukin-2: Taming Protein Hydrophobicity and Aggregation by a Versatile Solubilizing Strategy

Jingwen Zeng,[a] Haiyan Zhou,[a] Wang Xia,[a,d] Hongxiang Wu,[a,e] and Xuechen Li*[a,b,c]

[a] Department of Chemistry, State Key Laboratory of Synthetic Chemistry, The University of Hong Kong, Pokfulam Road, Hong Kong SAR, 999077, P. R. China, E-mail: xuechenl@hku.hk

[b] Laboratory for Marine Drugs and Bioproducts, Qingdao Marine Science and Technology Center, School of Medicine and Pharmacy, Ocean University of China, Qingdao, 266237, P. R. China

[c] Shanghai-Hong Kong Joint Laboratory in Chemical Synthesis, Shanghai Institute of Organic Chemistry, University of Chinese Academy of Sciences, Chinese Academy of Sciences, 345 Lingling Road, Shanghai, 200032, P. R. China.

[d] Current address: School of Environmental and Chemical Engineering, Wuyi University, Jiangmen, 529020, P.R. China.

[e] Current address: Zhongshan Institute for Drug Discovery, Shanghai Institute of Materia Medica, Chinese Academy of Sciences, Zhongshan, 528400, P. R. China

Contents

1. General Information 3

2. General Experimental Procedures 4

3. Synthesis of Building Blocks 10

4. Attempts to the Synthesis of IL-15 using TBM 17

5. Synthesis of Wild-type and N79-Glycosylated IL-15 41

6. Synthesis of IL-2-WT and IL-2-AzK 58

7. CTLL-2 Proliferation Assay for Characterization of IL-15 and IL-2 78

8. NMR Data 80

9. Reference 88

1. General Information

1.1 Materials, methods and abbreviations

All commercial materials (purchased from Aldrich, ChemImpex, Fluka and GL Biochem) were used without further purification. All solvents were reagent grade or HPLC grade (RCI or DUKSAN). Dry dichloromethane (CH_2Cl_2) was distilled from calcium hydride (CaH_2). All reversed-phase (RP) high-performance liquid chromatography (HPLC) separations involved a mobile phase of 0.1% trifluoroacetic acid (TFA) (v/v) in acetonitrile (CH_3CN)/0.1% TFA (v/v) in water (H_2O) were performed with a Waters HPLC system equipped with a photodiode array detector (Waters 2996) using a Vydac 214TPTM C4 column (5 μm, 300 Å, 4.6 x 250 mm) at a flow rate of 0.6 mL/min for analytical HPLC and Vydac 214TPTM C4 column (10 μm, 300 Å, 22 x 250 mm) or Vydac 218TPTM C18 column (10 μm, 300 Å, 22 x 250 mm) at a flow rate of 10 mL/min for preparative HPLC. Low-resolution mass spectral (MS) analyses were performed with a Waters 3100 mass spectrometer using electrospray ionization (ESI, in positive mode unless otherwise specified). The results were analyzed with Waters Empower software. Calculated masses were based on the most abundant isotope of a given ion. Analytical TLC was performed on E. Merck silica gel 60 F254 plates and visualized under UV light (254 nm) or by staining with ninhydrin or 5 % sulfuric acid in methanol. Silica flash column chromatography was performed on E. Merck 230-400 mesh silica gel 60. ^{1}H and ^{13}C nuclear magnetic resonance (NMR) spectra were recorded at 298 K on Bruker Avance DRX 300 FT-NMR Spectrometer at 75 MHz for ^{13}C NMR or Bruker Avance DRX 400 FT-NMR spectrometer at 400 MHz for ^{1}H NMR and 100 MHz for ^{13}C NMR or Bruker Avance DRX 600 FT-NMR spectrometer at 150 MHz for ^{13}C NMR. Chemical shifts are reported in parts per million (ppm) and are referenced to solvent residual signals: $CDCl_3$ (δ 7.26 [^{1}H]). ^{1}H NMR data is reported as chemical shift (δ), relative integral, multiplicity (s = singlet, d = doublet, t = triplet, dd = doublet of doublet, td = triplet of doublet), coupling constant (J Hz). LCMS = Liquid chromatography mass-spectrometry; PG = protecting groups; SAL = salicylaldehyde; DMF = dimethylformamide; TIPS = triisopropylsilane.

2. General Experimental Procedures

2.1 Standard Protocol of Fmoc Solid-phase Peptide Synthesis (Fmoc-SPPS)

The commercially available 2-chlorotrityl resin (CS Biochem, loading: ~0.5 mmol/g) was employed in the solid phase peptide synthesis of peptides. FmocHN-Xaa-COOH (4.0 equiv.) and diisopropylethylamine (8.0 equiv.) were dissolved in dichloromethane, then this solution was poured into a reaction vial containing 2-chlorotrityl resin, and the mixture was shaken at room temperature (rt) for 2 h. After that, this amino acid bound resin was washed with dimethylformamide and dichloromethane. The resin was subjected to iterative coupling to give the corresponding peptide product. For peptides with C-terminal amide, the commercially available Rink amide AM resin (Chemimpex, loading: ~0.29 mmol/g) was employed in the solid phase peptide synthesis of peptides. The resin was swelled in DMF for 15 min and subjected to Fmoc deprotection by 20% piperidine DMF solution. After that, this resin was washed with dimethylformamide and dichloromethane. The resin was subjected to iterative coupling to give the corresponding peptide product. The following Fmoc amino acids and Boc amino acids from CS Biochem were employed: Fmoc-Ala-OH, Fmoc-Cys(Trt)-OH, Fmoc-Cys(S^{t}Bu)-OH, Fmoc-Asp(O^{t}Bu)-OH, Fmoc-Glu(O^{t}Bu)-OH, Fmoc-Phe-OH, Fmoc-Gly-OH, Fmoc-His(Trt)-OH, Fmoc-His(Boc)-OH, Fmoc-Ile-OH, Fmoc-Lys(Boc)-OH, Fmoc-Leu-OH, Fmoc-Met-OH, Fmoc-Asn(Trt)-OH, Fmoc-Pro-OH, Fmoc-Gln(Trt)-OH, Fmoc-Arg(Pbf)-OH, Fmoc-Ser(^{t}Bu)-COOH, Fmoc-Thr(^{t}Bu)-COOH, Fmoc-Val-OH, Fmoc-Trp(Boc)-OH, Fmoc-Tyr(^{t}Bu)-OH, Boc-Ser(^{t}Bu)-COOH, Boc-Thr(^{t}Bu)-COOH. After each coupling, the resin was washed with DMF and DCM.

The obtained resin bound peptide was treated with TFA cocktail for 2 h. After that, the peptide TFA solution was poured into 50 mL diethyl ether, and the resulting suspension was centrifuged to give a white pellet. After decanting diethyl ether, the remaining peptide was dissolved in CH_3CN/H_2O or 6M GnHCl for HPLC purification.

2.2 RST-2.0 for the synthesis of hydrophobic and aggregating peptides

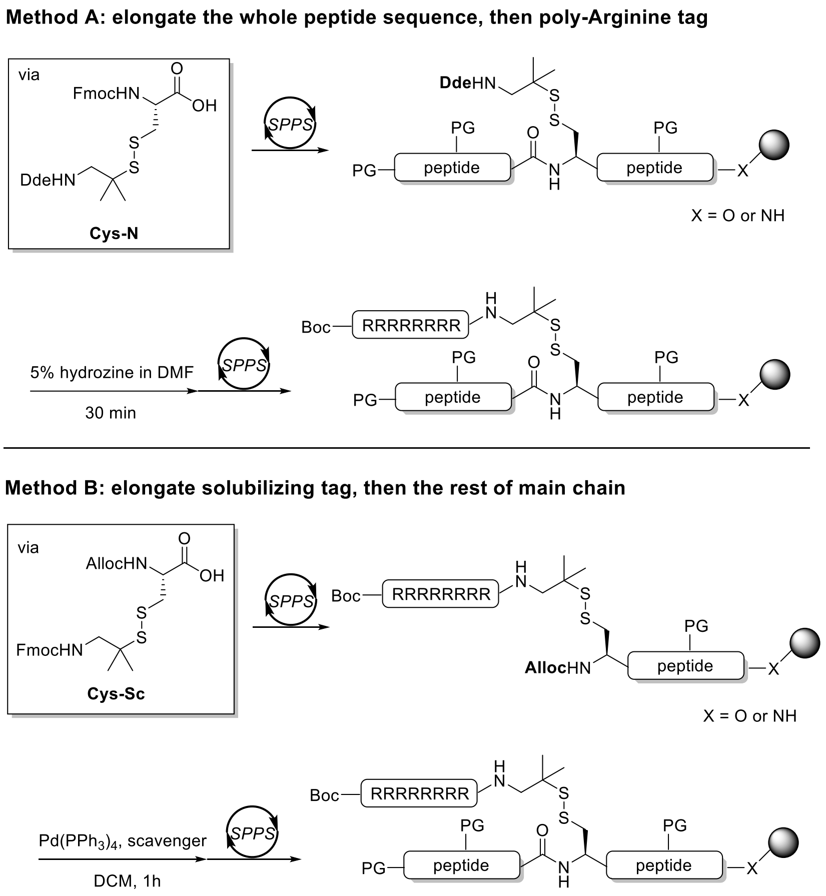

Scheme S1. Workflow of RST-2.0 via **Cys-N** or **Cys-Sc**.

Method A: **Cys-N** was used as Cysteine building blocks during Fmoc-SPPS. After elongating the full length of the peptides, the peptide bound resin was treated with 5% hydrazine in DMF for 30 min. The resin was washed with DMF/DCM/DMF three times, followed by SPPS of solubilizing tag.

Method B: **Cys-Sc** was used as Cysteine building blocks during Fmoc-SPPS. After elongating the half length of the peptides and the solubilizing tag, the N-terminal Alloc was removed (0.5 equiv. $Pd(PPh_3)_4$, 2 equiv. 1,3-Dimethylbarbituric acid, DCM, 1h). After that, the resin was washed with DMF/DCM/DMF three times, followed by SPPS of the rest of peptide sequence.

2.3 Soft Cleavage of fully protected crude peptide from resin

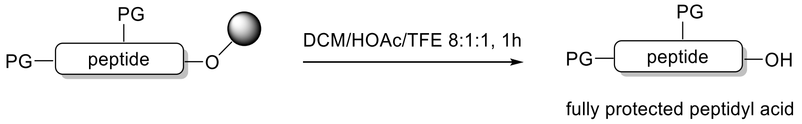

After the standard Fmoc-SPPS, the peptide bound 2-chlorotrityl resin resin was washed with dichloromethane three times. Subsequently, the resin was treated with cocktail A (DCM/HOAc/trifluoroethanol = 8/1/1) for 1 h. After that, the mixture was filtrated to give the solution of the desired peptidyl acid. The protected peptide solution was concentrated and co-evaporated with DCM/hexane mixture six times to give a white powder without HOAc.

2.4 Synthesis of C-terminus '1' (L)-Amino acid salicylaldehyde semicarbazone ester hydrochloride (HCl·H_2N-Xaa-CO-SAL^{off})

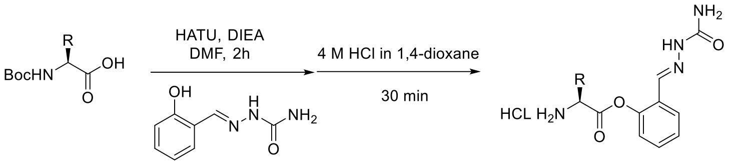

BocHN-Xaa(PG)-COOH, (1.0 equiv.) was dissolved in DMF, followed by the addition of HATU (1.0 equiv.) and DIPEA (2.0 equiv.). The solution was stirred for 2 min, then salicylaldehyde semicarbazone (1.0 equiv.) was added. The reaction mixture was stirred for 2h and then extracted with EA and washed with brine. The organic layer was removed by reduce pressure evaporation, and the residue was purified by silica gel chromatography (DCM/EA, 2:1) to give the desired BocHN-Xaa(PG)-CO-SAL^{off} product. This product was treated with a solution of HCl/dioxane (4 M) for 30 min, and the solvent was removed by co-evaporation with toluene under reduced pressure. Without purification, this salt was directly used in the "N+1" Sakakibara elongation.

2.5 Synthesis of C-terminal Peptide SAL esters using N+1 strategy

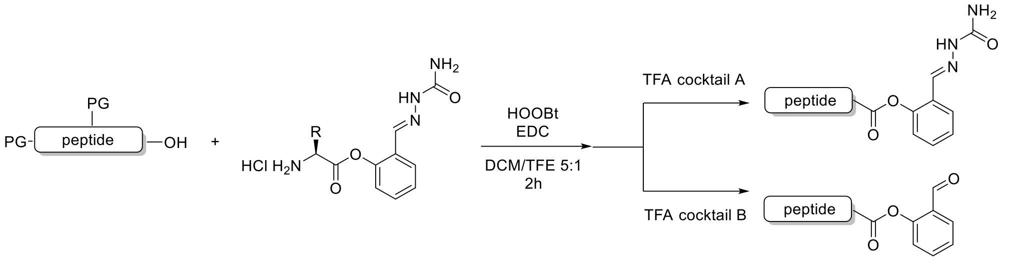

The fully protected peptidyl acid (0.1 mmol, 1.0 equiv.), obtained as described in the previous section 2.2, was dissolved in DCM (2 mM), then N-(3 dimethylaminopropyl)-N'-ethylcarbodiimide (EDC) (3.0 equiv.) and hydroxy-3,4 dihydro-4-oxo-1,2,3-benzotriazine (HOOBt) (3.0 equiv.) were added. After 5 min, the corresponding amino L-Amino acid salicylaldehyde semicarbazone ester hydrochloride (HCl·H_2N-Xaa-CO-SAL^{off}) (5.0 equiv.), obtained as described in the previous section, was added, and the reaction mixture was stirred for 2 h to form the crude protected C-terminal peptide SAL^{off} ester. This reaction mixture was subjected to TFA cocktail A (TFA/H_2O = 95:5) or TFA cocktail B (TFA/H_2O/acetylacetone = 95:2.5:2.5) for global deprotection to generate peptide SAL^{off} ester and peptide SAL^{on} ester, respectively. The TFA solution was poured into a cold diethyl ether followed by centrifugation to afford a white solid. After that, the diethyl ether layer was decanted, and the crude product was subjected to HPLC purification to afford the desired peptide SAL ester.[1]

2.6 Synthesis of N,S-Benzylidene Thioacetals (NBTs) for introducing TBM

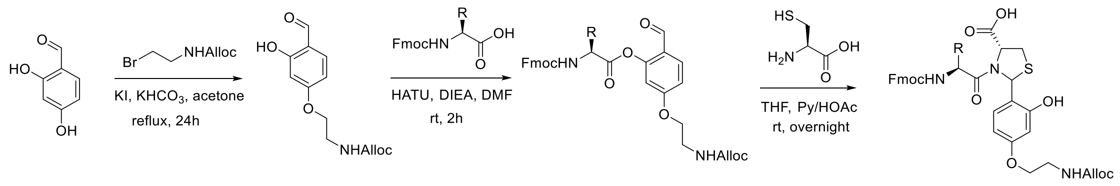

2,4-Dihydroxybenzaldehyde (100 mmol), potassium bicarbonate ($KHCO_3$, 100 mmol), and potassium iodide (KI, 10 mmol) were combined in 300 mL of acetone. To this mixture, allyl (2-bromoethyl)carbamate (100 mmol) was added, and the reaction was refluxed overnight. After completion, the mixture was diluted with 1.5 L of ethyl acetate

and sequentially washed with 2 L of water and 2 L of brine. The organic layer was dried over anhydrous sodium sulfate (Na_2SO_4) and concentrated under reduced pressure. The crude product was purified using silica gel chromatography with a n-hexane/ethyl acetate (5:1) eluent, yielding allyl (2-(4-formyl-3-hydroxyphenoxy)ethyl)carbamate as a white solid.

Fmoc-Xaa-OH, (2-(4-formyl-3-hydroxyphenoxy)ethyl)carbamate (1.0 equiv), DMAP (1 mol%), and EDCI (1.05 equiv) were dissolved in dichloromethane (DCM), and the mixture was stirred at room temperature for 2 hours. Then the mixture was washed sequentially with 1 M HCl and brine, then dried over anhydrous sodium sulfate. The solvent was removed under reduced pressure, leaving an oily residue. This residue was dissolved in THF containing 30% pyridine-acetic acid. In a separate centrifuge tube, L-H-Cys-OH was combined with TFA (2.0 equiv) and 20% volume water to aid dissolution, and the mixture was sonicated until a clear solution formed. This solution was added dropwise to the SAL ester solution, and the reaction was stirred at room temperature overnight, with progress monitored by TLC. After completion, the mixture was washed with water and brine, dried over anhydrous sodium sulfate, and concentrated under vacuum. The crude product was purified by column chromatography, yielding the desired Fmoc-Xaa-Cys-OH dipeptide as a white solid.[2]

2.7 Removal of acetyl protecting groups of carbohydrate on glycopeptide

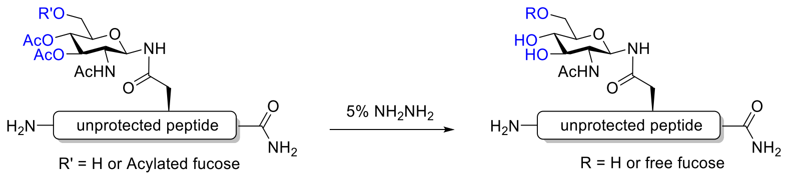

After global deprotection and ether precipitation, glycopeptide was dissolved in 8M GnHCl containing 5% hydrazine. The acetyl deprotection was monitored by UPLC-MS. After completion of the reaction, the pH of reaction mixture was adjusted to around 5.0 by adding 6N HCl, and the product was purified by HPLC to give glycopeptide as a white fluffy powder.

2.8 Microscale Thermophoresis (MST)

IL-2Rα (Sino Biological, 10165-H02H, Fc tag) was labelled with Alexa Fluor 647 NHS ester dye (Thermo Fisher) according to the manufacturer's instructions. The remaining dye in the reaction mixture was removed by buffer exchange through ultrafiltration (MWCO 3000, at least six rounds of dilution-centrifugation) using PBS. Degree of labelling (DOI, controlled between 0.5 to 1) was determined by measuring UV absorption at 280 nm and 650 nm. Calculation (ε: Extinction coefficients):

$$C_{IL2R\alpha} = A_{280} - 0.03A_{650}/\varepsilon$$

$$C_{dye} = A_{650}/\varepsilon$$

$$DOI = C_{dye}/C_{protein}$$

The labelled IL-2Rα was aliquoted and stored at -80 °C and used within a week. Before MST assay, the protein was thawed and subjected to centrifugation (20000×g, 10 min) and then the supernatant was diluted in PBST (PBS supplemented with 0.1% Tween 20) to 40 nM for binding measurement. IL-2 proteins were prepared at serial concentrations and mixed with labelled IL-2 Rα in a 1:1 ratio (5 μL+5 μL). MST assay was performed using a Monolith X instrument (NanoTemper Technologies) at room temperature. Instrument parameters were adjusted to 20% LED power and medium MST power. Each experiment was performed in triplicates. K_d of IL-2 analogs was given by Monolith. Note: all proteins should be centrifugated at 20000×g for 10 min before used, and the protein concentration was determined by Thermo Scientific NanoDrop UV-Vis spectrophotometer.

3. Synthesis of Building Blocks

3.1 Synthesis of disulfide linkers

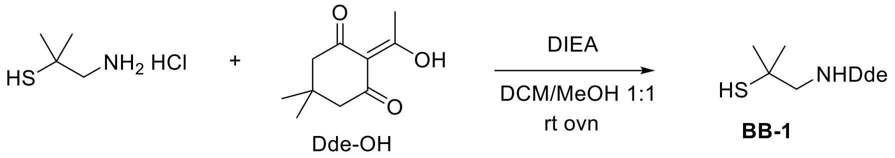

1-amino-2-methylpropane-2-thiol hydrochloride (2.84 g, 20 mmol, 1 equiv.) was suspended in a solution of Dde-OH (3.64 g, 20 mmol, 1 equiv.) in 50 mL DCM/MeOH 1:1. Then, DIEA (3.5 mL, 20 mmol, 1 equiv.) was added dropwise. The above solution was stirred vigorously overnight at room temperature. The solution was evaporated in vacuum to remove the solvent, then the residue was dissolved into 100 mL EA and washed with 100 mL 1 N HCl twice and 100 mL saturated NaCl. The upper organic phase was dried in Na_2SO_4 and concentrated in vacuum. The resulting crude product could be directly used for the synthesis of **Cys-N** without further purification. For purification, when necessary, the crude solid was purified by flash chromatography on silica gel with a mixture of Hexane/EA 3:1 as eluent to give compound **BB-1** as a yellow solid (3.87 g, 72% yield).

^{1}H NMR (500 MHz, $CDCl_3$) δ 13.86 (s, 1H), 3.49 (d, J = 5.6 Hz, 2H), 2.58 (s, 4H), 2.39 (s, 5H), 1.92 (s, 1H), 1.47 (s, 7H), 1.04 (s, 7H).

^{13}C NMR (126 MHz, $CDCl_3$) δ 199.34, 196.90, 173.53, 108.16, 57.12, 53.60, 52.24, 43.61, 30.10, 28.30, 18.08, 0.00.

HRMS (ESI-TOF) m/z of **BB-1**: $[M+H]^+$ Calcd for $[C_{14}H_{24}NO_2S]^+$ = 270.1522; Found 270.1522.

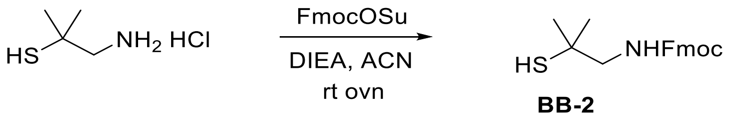

1-amino-2-methylpropane-2-thiol hydrochloride (8.5 g, 60 mmol, 1.2 equiv.) was suspended in a solution of Fmoc-Osu (16.8 g, 50 mmol, 1 equiv.) in 500 mL CH_3CN. Then, DIEA (17.5 mL, 100 mmol, 2 equiv.) was added dropwise. The above solution was stirred vigorously overnight at room temperature. The solution was evaporated in

vacuum to remove the solvent, then the residue was dissolved into 100 mL EA and washed with 100 mL 1 N HCl twice and 100 mL saturated NaCl. The upper organic phase was dried with Na_2SO_4 and concentrated in vacuum. The resulting crude product could be directly used for the synthesis of **Cys-Sc** without further purification or, when necessary, be purified by flash chromatography on silica gel with a mixture of Hexane/EA 4:1 as eluent to give compound **BB-2** as a yellow solid (12.2 g, 75% yield).

^{1}H NMR (500 MHz, $CDCl_3$) δ 7.77 (d, *J* = 7.5 Hz, 1H), 7.62 (d, *J* = 7.3 Hz, 1H), 7.41 (t, *J* = 7.5 Hz, 1H), 7.32 (t, *J* = 7.4 Hz, 1H), 5.25 (s, 1H), 4.45 (d, *J* = 6.9 Hz, 1H), 4.24 (t, *J* = 6.8 Hz, 1H), 3.28 (d, *J* = 6.5 Hz, 1H), 1.64 (s, 1H), 1.36 (s, 3H).

^{13}C NMR (126 MHz, $CDCl_3$) δ 156.65, 143.92, 141.35, 127.71, 127.07, 125.04, 120.00, 66.75, 54.22, 47.32, 45.51, 29.75.

HRMS (ESI-TOF) m/z of **BB-2**: $[M+H]^+$ Calcd for $[C_{19}H_{22}NO_2S]^+$ = 328.1366; Found 328.1366.

3.2 Synthesis of Cys-N

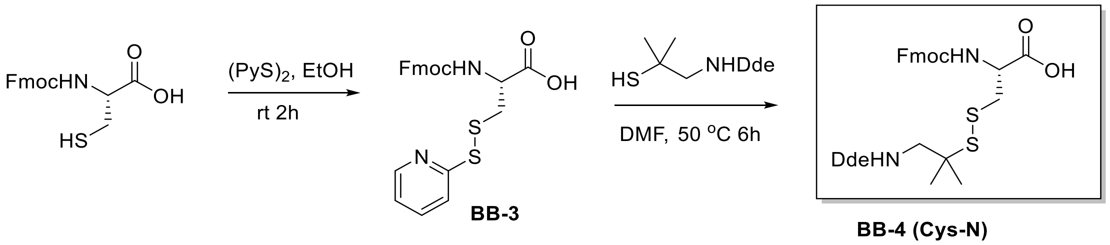

Step 1:

To the solution of Fmoc-Cys-OH (3.43 g, 10 mmol) in 50 mL EtOH was added DTDP (3.0 g, 13.6 mmol). The solution was stirred for 2 h until TLC indicated the completion of reaction. EtOH was removed by rotary evaporation under vacuum. The residue was dissolved in 20 mL DCM and subjected to silica gel chromatography. The desired product was eluted with Hexane/EA in 2:1 ratio (containing 1% HOAc). After concentration in vacuum, the product crystallized as a white solid (3.8 g, 84% yield). Compound **BB-3** showed poor solubility in most organic solvents other than highly polar solvents such as DMF.

^{1}H NMR (500 MHz, *d6*-DMSO) δ 8.47 (d, *J* = 4.7 Hz, 1H), 7.98 (d, *J* = 8.2 Hz, 1H),

7.94 (d, J = 7.5 Hz, 2H), 7.77 (t, J = 4.9 Hz, 4H), 7.46 (t, J = 7.4 Hz, 2H), 7.37 (t, J = 7.3 Hz, 2H), 7.27 (dd, J = 8.8, 4.4 Hz, 1H), 4.37 (d, J = 7.0 Hz, 2H), 4.30 (td, J = 7.9, 4.1 Hz, 2H), 3.27 (dd, J = 13.7, 4.2 Hz, 2H), 3.16 (dd, J = 13.7, 10.0 Hz, 1H).

^{13}C NMR (126 MHz, *d6*-DMSO) δ 172.41, 159.02, 156.44, 150.11, 144.25, 144.21, 141.20, 138.24, 128.14, 127.57, 125.68, 121.81, 120.61, 119.89, 66.20, 53.46, 47.09.

HRMS (ESI-TOF) m/z of **BB-3**: $[M+H]^+$ Calcd for $[C_{23}H_{21}N_2O_4S_2]^+$ = 453.0937; Found 453.0936.

Step 2:

Compound **BB-3** (2.76 g, 6.1 mmol) and compound **BB-1** (2.46 g, 9.1 mmol) were dissolved into 30 mL DMF and stirred vigorously at 50 °C for 6 h. The mixture was diluted with 100 mL DCM and washed with 150 mL 1 N HCl, water, saturated NaCl and dried in Na_2SO_4. After concentrating in vacuum, the product was purified by silica gel chromatography using Hexane/EA (1% HOAc) as eluent with polarity increasing from 2:1 to 1:1.5 by gradient. The product was collected and concentrated in vacuum to give a white power (3.8 g, 99% yield).

^{1}H NMR (500 MHz, *d6*-DMSO) δ 13.61 (s, 1H), 7.93 (d, J = 7.5 Hz, 2H), 7.83 (d, J = 8.4 Hz, 1H), 7.75 (d, J = 7.4 Hz, 2H), 7.45 (t, J = 7.5 Hz, 2H), 7.36 (t, J = 7.4 Hz, 2H), 5.78 (s, 1H), 4.32 (ddt, J = 21.5, 13.8, 7.2 Hz, 5H), 3.62 (d, J = 5.0 Hz, 3H), 3.13 (dd, J = 13.3, 4.1 Hz, 2H), 3.02 (dd, J = 13.2, 10.3 Hz, 1H), 2.54 (s, 5H), 2.31 (s, 4H), 1.37 (d, J = 5.1 Hz, 6H), 0.97 (s, 6H).

^{13}C NMR (126 MHz, *d6*-DMSO) δ 173.32, 172.42, 156.44, 144.22, 141.19, 128.13, 127.58, 125.71, 120.59, 107.73, 66.22, 55.36, 53.66, 51.41, 50.35, 47.08, 41.31, 30.17, 28.34, 25.81, 25.68, 18.05.

HRMS (ESI-TOF) m/z of **BB-4** (**Cys-N**): $[M+H]^+$ Calcd for $[C_{32}H_{39}N_2O_6S_2]^+$ = 611.2244; Found 611.2235.

3.3 Synthesis of Cys-Sc

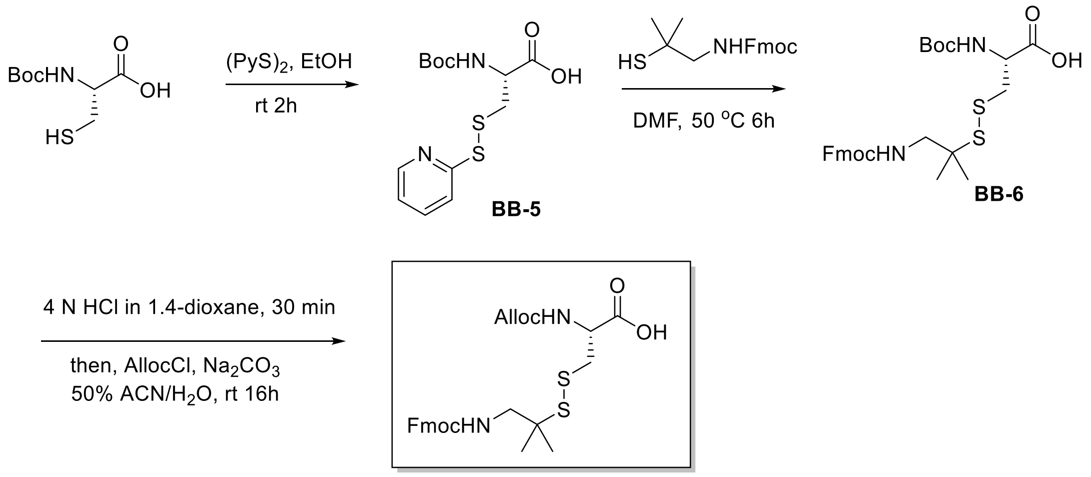

BB-7 (Cys-Sc)

Step 1:

To the solution of Boc-Cys-OH (7.0 g, 31.6 mmol) in 600 mL EtOH was added DTDP (10.5g, 47.5 mmol). The solution was stirred for 2 h until TLC indicated the completion of reaction. EtOH was removed by rotary evaporation under vacuum. The residue was dissolved in DCM and subjected to silica gel chromatography. The column was washed with Hexane/EA in 2:1 ratio, then desired product was eluted with Hexane/EA in 2:1 ratio containing 1% HOAc. After concentration in vacuum, product **6** was obtained as a white powder (8.5 g, 82% yield).

^{1}H NMR (500 MHz, *d6*-DMSO) δ 8.50 (d, *J* = 4.5 Hz, 1H), 7.86 – 7.77 (m, 2H), 7.41 (d, *J* = 8.2 Hz, 1H), 7.31 – 7.26 (m, 1H), 4.21 (td, *J* = 9.3, 4.2 Hz, 1H), 3.21 (dd, *J* = 13.6, 4.2 Hz, 1H), 3.13 (dd, *J* = 13.6, 9.9 Hz, 1H), 1.43 (s, 10H).

^{13}C NMR (126 MHz, *d6*-DMSO) δ 172.65, 159.24, 155.83, 150.08, 138.25, 121.74, 119.79, 78.85, 53.23, 28.63.

HRMS (ESI-TOF) m/z of **BB-5**: $[M+H]^+$ Calcd for $[C_{13}H_{19}N_2O_4S_2]^+$ = 331.0781; Found 331.0780.

Step 2:

Compound **6** (5.28 g, 16 mmol) and compound **7** (6.86 g, 21 mmol) were dissolved into 50 mL DMF and stirred vigorously at 50 °C for 6 h. The mixture was diluted with 200 mL DCM and washed with 300 mL 1 N HCl, water, saturated NaCl and dried in Na_2SO_4.

After concentrating in vacuum, the product was purified by silica gel chromatography. The column was washed with Hexane/EA in 3:1 ratio, then desired product was eluted with Hexane/EA in 3:1 ratio containing 1% HOAc. The product **8** was collected and concentrated in vacuum to give a white power (8.76 g, 99% yield).

^{1}H NMR (500 MHz, $CDCl_3$) δ 8.26 (s, 1H), 7.75 (d, *J* = 7.5 Hz, 2H), 7.61 (t, *J* = 6.9 Hz, 2H), 7.55 (d, *J* = 7.1 Hz, 1H), 7.39 (t, *J* = 7.4 Hz, 2H), 7.31 (t, *J* = 7.4 Hz, 2H), 6.44 – 6.07 (m, 1H), 5.62 – 5.20 (m, 2H), 4.63 (s, 1H), 4.52 (d, *J* = 5.3 Hz, 1H), 4.41 (d, *J* = 6.9 Hz, 1H), 4.21 (t, *J* = 6.7 Hz, 1H), 3.33 (d, *J* = 6.2 Hz, 1H), 3.28 – 3.03 (m, 3H), 1.46 (s, 10H), 1.27 (s, 5H), 1.08 (d, *J* = 15.1 Hz, 2H).

^{13}C NMR (126 MHz, $CDCl_3$) δ 174.09, 156.95, 155.43, 143.89, 141.32, 127.73, 127.12, 125.13, 120.00, 80.57, 66.98, 53.23, 51.41, 49.01, 47.26, 41.67, 28.37, 25.18.

HRMS (ESI-TOF) m/z of **BB-6**: $[M+H]^+$ Calcd for $[C_{27}H_{35}N_2O_6S_2]^+$ = 547.1931; Found 547.1925.

Step 3:

To a solution of compound **8** (2.3 g, 4.2 mmol) in 20 mL 1,4-dioxane was added 20 mL 37% HCl. After 30 min, the solvent was evaporated in vacuum. The residue was dissolved in 30 mL ACN/H_2O (v/v, 50/50), followed by adding Na_2CO_3 (0.89 g, 8.4 mmol) and Alloc-Cl (0.46 mL, 4.2 mmol). After 16 h reaction at room temperature, the ACN was removed by rotary evaporation, and the residue was acidified with 1 N HCl and extracted with 150 mL EA, dried with Na_2SO_4 and concentrated in vacuum. The crude product was purified by silica gel chromatography with Hexane/EA 2:1 (containing 1% HOAc) as eluent. After concentration in vacuum, product **9** was obtained as a white solid (1.84 g, 82% yield).

^{1}H NMR (500 MHz, $CDCl_3$) δ 8.72 (s, 1H), 7.75 (d, *J* = 7.5 Hz, 2H), 7.61 (d, *J* = 6.8 Hz, 1H), 7.54 (d, *J* = 7.3 Hz, 1H), 7.39 (t, *J* = 7.4 Hz, 2H), 7.31 (t, *J* = 7.4 Hz, 2H), 6.33 (dd, *J* = 64.8, 32.2 Hz, 1H), 5.90 (ddd, *J* = 22.7, 10.8, 5.6 Hz, 1H), 5.80 (d, *J* = 7.5 Hz, 1H), 5.39 (d, *J* = 5.6 Hz, 1H), 5.31 (d, *J* = 17.2 Hz, 1H), 5.20 (t, *J* = 8.8 Hz, 1H), 4.70 (dd, *J* = 11.8, 7.1 Hz, 1H), 4.60 (s, 2H), 4.53 (d, *J* = 5.4 Hz, 1H), 4.41 (d, *J* = 7.0 Hz, 1H), 4.21 (t, *J* = 6.8 Hz, 1H), 3.29 (dd, *J* = 23.7, 9.9 Hz, 2H), 3.14 (dd, *J* = 13.9, 5.6 Hz,

1H), 3.02 (d, J = 5.6 Hz, 1H), 2.90 (dd, J = 13.8, 8.3 Hz, 1H), 1.26 (s, 4H), 1.10 (s, 1H), 1.00 (s, 1H).

^{13}C NMR (126 MHz, $CDCl_3$) δ 174.26, 173.70, 158.09, 156.99, 156.19, 155.91, 143.87, 143.62, 141.45, 141.33, 132.67, 132.45, 127.87, 127.74, 127.18, 127.12, 125.13, 124.71, 120.01, 118.08, 117.73, 67.48, 66.99, 66.16, 54.58, 53.56, 51.47, 50.50, 49.24, 49.07, 47.25, 47.08, 42.29, 41.51, 25.37, 25.17, 24.28.

HRMS (ESI-TOF) m/z of **BB-7** (**Cys-Sc**): $[M+H]^+$ Calcd for $[C_{26}H_{31}N_2O_6S_2]^+$ = 531.1618; Found 531.1616.

3.4 Synthesis of Dde-Ser(^{t}Bu)-OH

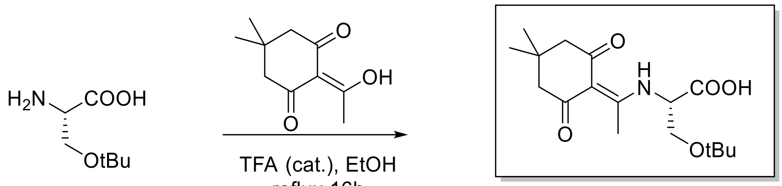

BB-8 (Dde-Ser(^{t}Bu)-OH)

To a solution of H-Ser(^{t}Bu)-OH (0.97 g, 6 mmol, 1.2 equiv.), Dde-OH (0.91 g, 5 mmol, 1 equiv.) in ethanol was added catalytic amount of TFA (38 μL, 0.1 equiv.). The solution was stirred reflux for 16 h. The solution was evaporated in vacuum and extracted with EA, washed with 1 N HCl and saturated NaCl, dried with Na_2SO_4 and concentrated in vacuum. The crude solid was purified by flash chromatography on silica gel with a gradient from Hexane/EA/HOAc 1:1/0.01 to pure EA containing 1% HOAc to give Dde-Ser(^{t}Bu)-OH as a white solid (1.25 g, 77% yield).

^{1}H NMR (500 MHz, *d6*-DMSO) δ 13.47 (d, J = 7.8 Hz, 1H), 4.83 – 4.55 (m, 1H), 3.86 (dd, J = 9.5, 3.5 Hz, 1H), 3.67 (dd, J = 9.6, 3.2 Hz, 2H), 2.46 (s, 3H), 2.33 (s, 4H), 1.17 (s, 9H), 0.99 (s, 6H).

^{13}C NMR (126 MHz, DMSO) δ 198.27, 195.97, 172.95, 170.68, 107.77, 73.72, 62.36, 56.55, 30.14, 28.33, 27.49, 18.28.

HRMS (ESI-TOF) m/z of **BB-8** (Dde-Ser(^{t}Bu)-OH): $[M+H]^+$ Calcd for $[C_{17}H_{28}NO_5]^+$ = 326.1962; Found 326.1963.

3.5 Synthesis of sugar building blocks

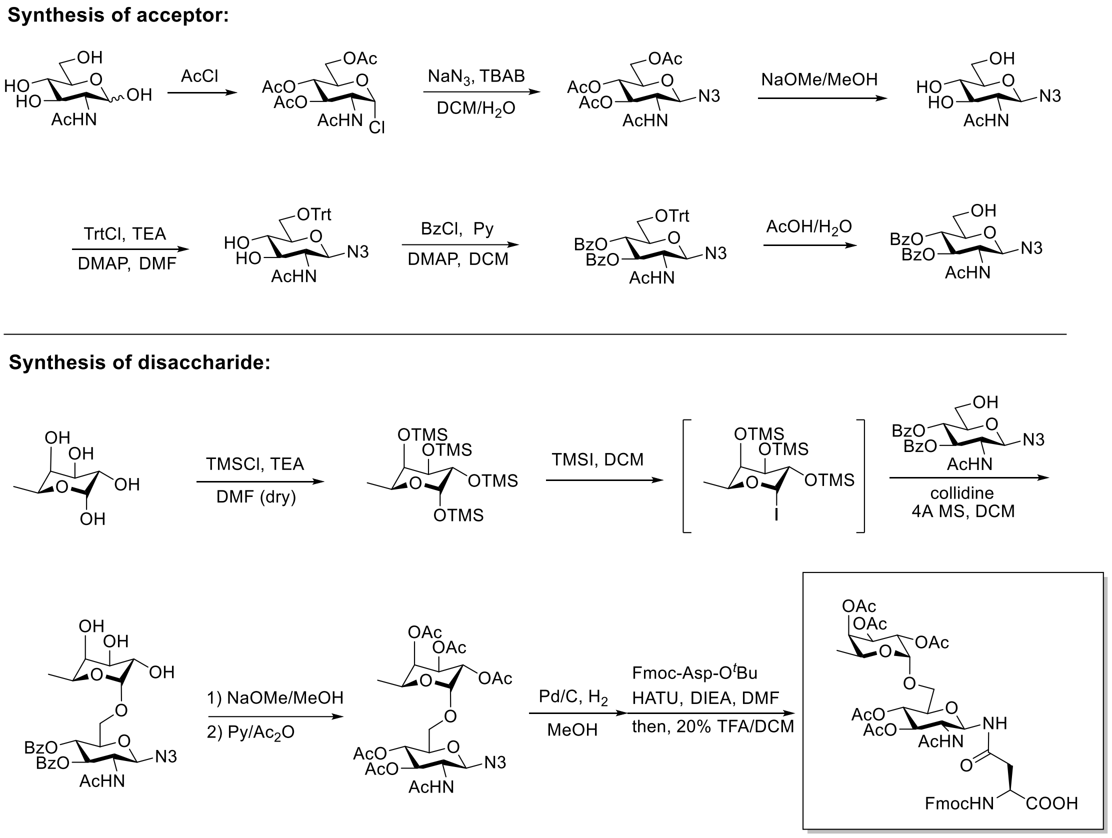

Scheme S2. Synthetic route for Fmoc-Asn(Fuc(α1,6)GlcNAc)-OH according to literature.[3]

4. Attempts to the Synthesis of IL-15 using TBM

4.1 Synthetic challenges

4.1.1 Truncation problem of IL-15-(1-25)

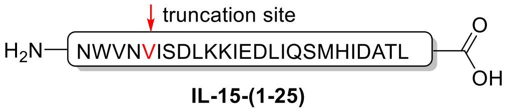

IL-15-(1-25)

Molecular Weight: 2896.3580

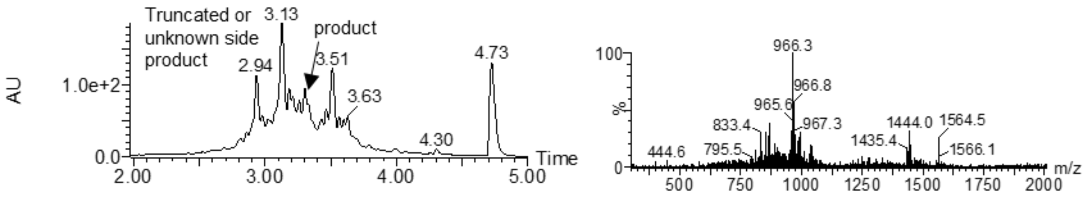

Figure S1. UV trace and MS spectrum of peptide **IL-15-(1-25)** synthesized by conventional Fmoc-SPPS. Peptide truncation (Val) and many unknown side products were observed.

4.4.2 Solubility problem of IL-15-(28-56)

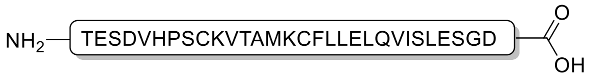

IL-15 seg2-(27-57)

Molecular Weight: 3280.7760

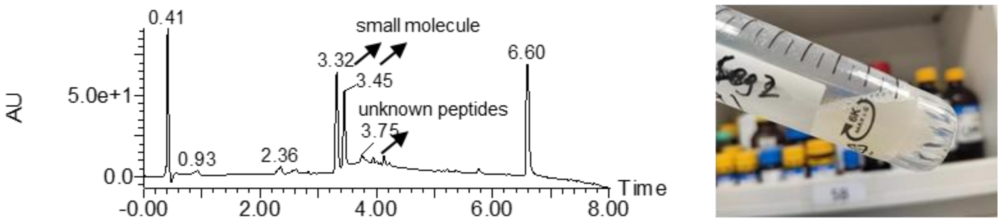

Figure S2. UV trace of peptide **IL-15-(28-56)** synthesized by conventional Fmoc-SPPS (left). Turbid solution of product in 8M GnHCl (right). Desired peptide was not observed in UPLC-MS. It was difficult to monitor the peptide elongation due to extremely poor solubility, so truncation beginning site (if any) was not determined.

4.1.3 Truncation and solubility problems of IL-15-(58-79)

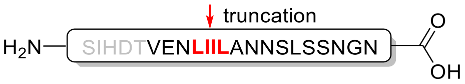

IL-15-(63-79)

Molecular Weight: 1771.9480

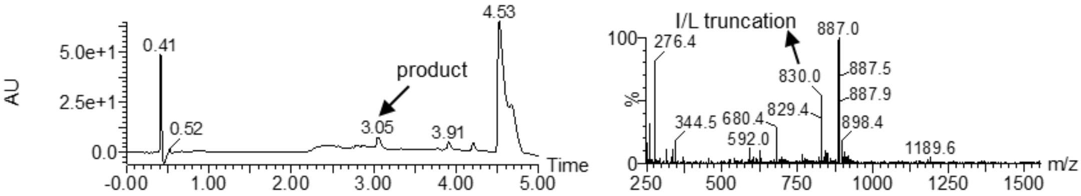

Figure S3. UV trace of peptide **IL-15-(63-79)** synthesized by conventional Fmoc-SPPS (left) and ESI-MS (right). Leu or Ile truncation was observed.

4.1.4 Truncation and aggregating problems of IL-15-(81-114)

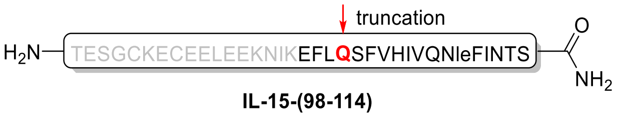

IL-15-(98-114)

Molecular Weight: 2040.3690

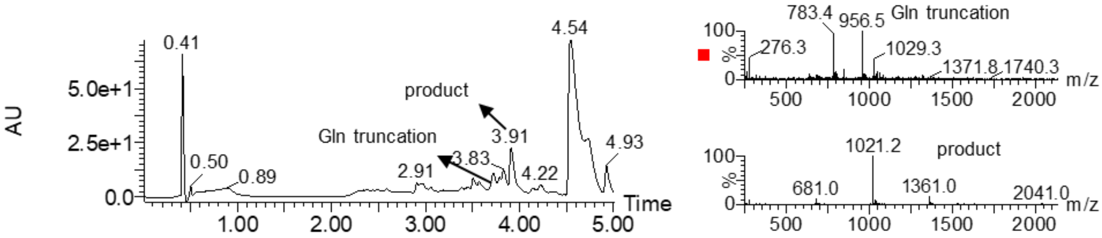

Figure S4. UV trace of peptide **IL-15-(98-114)** synthesized by conventional Fmoc-SPPS (left) and ESI-MS (right).

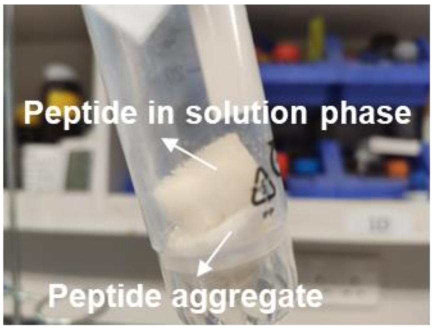

Figure S5. **IL-15-(81-114)** synthesized by NBDs-assisted SPPS. After global deprotection, the crude peptide was dissolved in 50% ACN. Around 5 minutes later, peptide assembled in the solution. Picture here was the crude peptide after centrifugation and lyophilization for roughly comparing the amount of soluble part and peptide aggregate part.

4.2 Limitations of RST-1.0

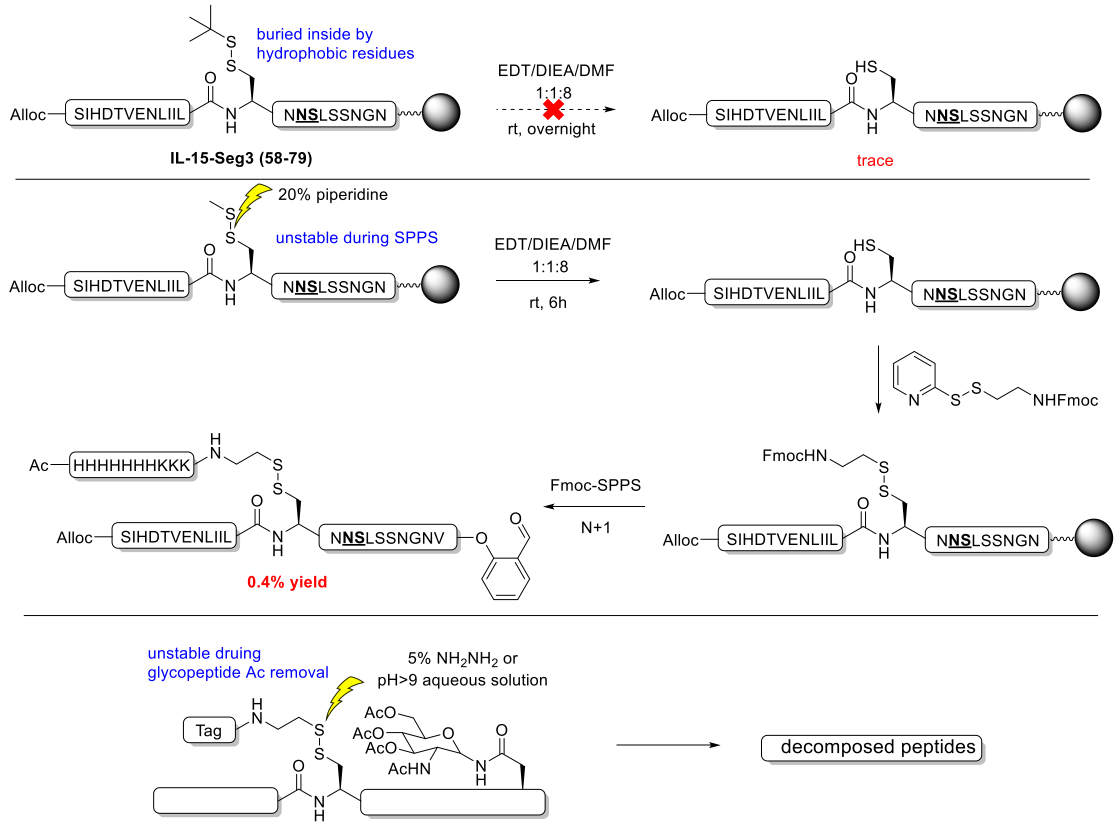

Figure S6. Initial protocol for RST installation. Removal of -S^{t}Bu protecting group is extremely difficult for hydrophobic peptides because Cys was buried by neighboring hydrophobic residues. Using -SMe as protecting group is not feasible because of low stability during SPPS (unknown mechanism, probably attacked by water in 20% piperidine solution) which results in low yield. Normal disulfides (except those protected by -S^{t}Bu) are also unstable during glycopeptide Ac removal.

4.3 Successful synthesis of IL-15 peptide segments

4.3.1 Synthesis of S1

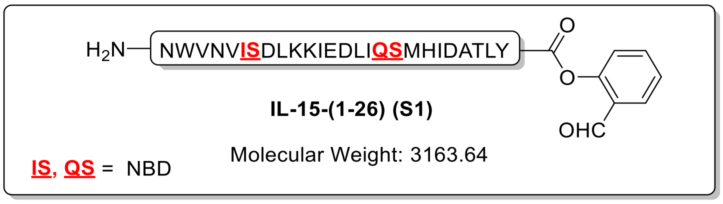

S1 was synthesized by general procedure **2.1** using 500 mg 2-chlorotrityl chloride resin (loading 0.5 mmol/g). Subsequently, following procedure **2.3**, **2.5**, TFA global

deprotection (30 mL, TFA/H_2O/acetylacetone = 95:2.5:2.5, 2h, using pyruvic acid can lead to lower yield), cold diethyl ether precipitation, HPLC purification (20-70% CH_3CN/H_2O over 30 min) and lyophilization, the desired peptide SAL ester **S1** was obtained as a white powder (260 mg, 33%).

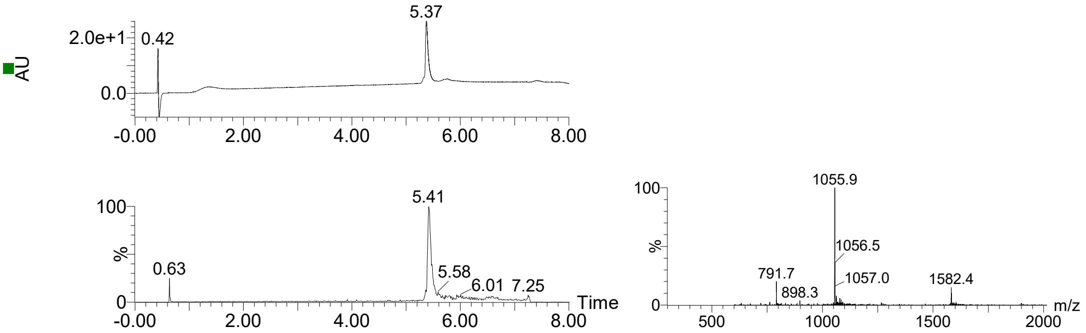

Figure S7. UPLC-MS analysis of purified peptide **S1**. Left: UV (190-400 nm) and MS (300-3000 m/z) trace from UPLC-MS analysis of purified **S1**, gradient 20-70% CH_3CN/H_2O containing 0.1% TFA over 8 min at a flow rate of 0.4 mL/min; Right: ESI-MS calcd. for $C_{145}H_{224}N_{34}O_{43}S$: $[M+2H]^{2+}$ m/z = 1582.8, found 1582.4; $[M+3H]^{3+}$ m/z = 1055.5, found 1055.9; $[M+4H]^{4+}$ m/z = 791.9, found 791.7.

4.3.2 Synthesis of S2

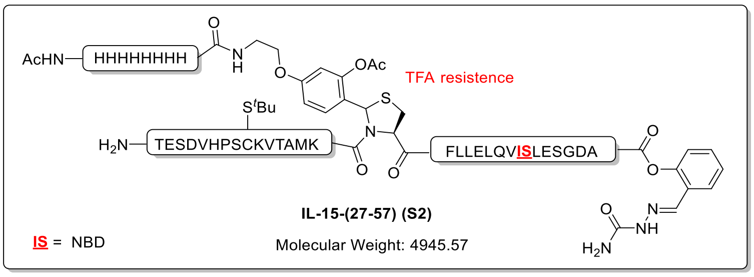

S2 was synthesized by general procedure **2.1** using 500 mg 2-chlorotrityl chloride resin (loading 0.5 mmol/g). KC-Alloc dipeptide was synthesized following procedure **2.6** for installation of TBM. Subsequently, Ac-capping was conducted by treating with Ac_2O/DIEA/DCM (2:4:100 v/v/v) cocktail. Following procedure **2.3**, **2.5**, TFA global deprotection (30 mL, TFA/H_2O = 95:5), cold diethyl ether precipitation, HPLC purification (15-60% CH_3CN/H_2O over 30 min) and lyophilization, the desired peptide SAL ester **S2** was obtained as a white powder (271 mg, 22%).

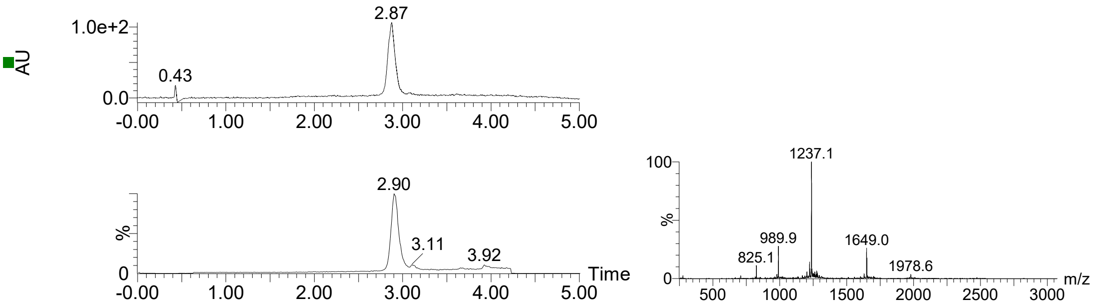

Figure S8. UPLC-MS analysis of purified peptide **S2**. Left: UV (190-400 nm) and MS (300-3000 m/z) trace from UPLC-MS analysis of purified **S2**, gradient 5-95% CH_3CN/H_2O containing 0.1% TFA over 5 min at a flow rate of 0.4 mL/min; Right: ESI-MS calcd. for $C_{217}H_{320}N_{64}O_{62}S_4$: $[M+3H]^{3+}$ m/z = 1649.5, found 1649.0; $[M+4H]^{4+}$ m/z = 1237.4, found 1237.1; $[M+5H]^{5+}$ m/z = 990.1, found 989.9; $[M+6H]^{6+}$ m/z = 825.2, found 825.1.

4.3.3 Synthesis of S3

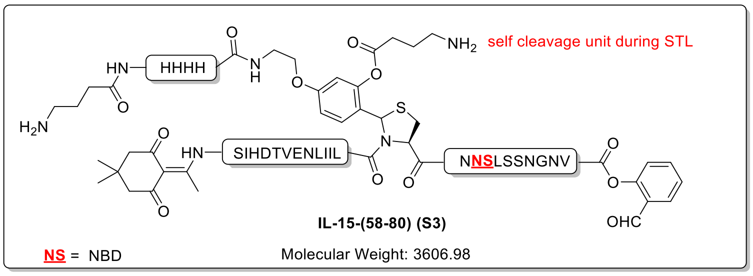

S3 was synthesized by general procedure **2.1** using 500 mg 2-chlorotrityl chloride resin (loading 0.5 mmol/g). LC-Alloc dipeptide was synthesized following procedure **2.6** for installation of TBM. Subsequently, Ac-capping was conducted by treating with Ac_2O/DIEA/DCM (2:4:100 v/v/v) cocktail. Following procedure **2.3**, **2.5**, TFA global deprotection (30 mL, TFA/H_2O/Pyruvic acid = 95:5:10), cold diethyl ether precipitation, HPLC purification (15-50% CH_3CN/H_2O over 30 min) and lyophilization, the desired peptide SAL ester **S3** was obtained as a white powder (210 mg, 23%).

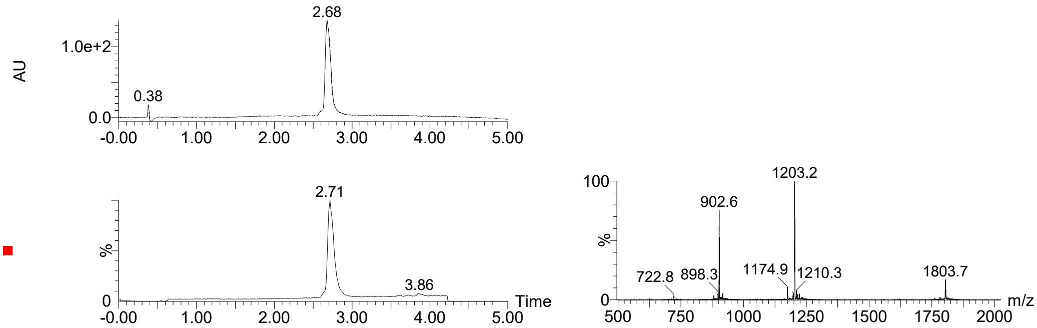

Figure S9. UPLC-MS analysis of purified peptide **S3**. Left: UV (190-400 nm) and MS (300-3000 m/z) trace from UPLC-MS analysis of purified **S3**, gradient 5-95% CH_3CN/H_2O containing 0.1% TFA over 5 min at a flow rate of 0.4 mL/min; Right: ESI-MS calcd. for $C_{160}H_{237}N_{45}O_{49}S$: $[M+2H]^{2+}$ m/z = 1804.5, found 1803.7; $[M+3H]^{3+}$ m/z = 1203.3, found 1203.2; $[M+4H]^{4+}$ m/z = 902.7, found 902.6.

4.3.4 Synthesis of S4

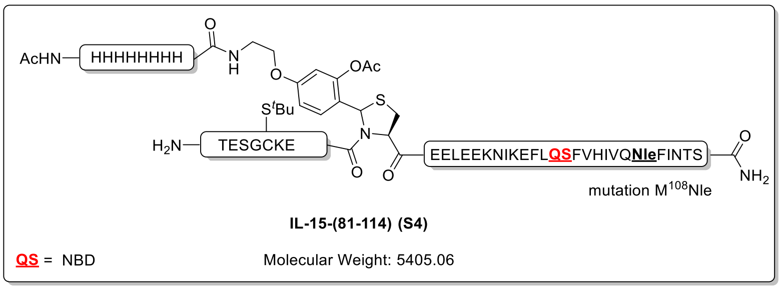

S4 was synthesized by general procedure **2.1** using 500 mg Rink Amide AM resin (loading 0.29 mmol/g). EC-Alloc dipeptide was synthesized following procedure **2.6** for installation of TBM. Subsequently, Ac-capping was conducted by treating with Ac_2O/DIEA/DCM (2:4:100 v/v/v) cocktail. Met^{108} was mutated to Nle^{108} for operational convenience. Subsequently, after TFA global deprotection (30 mL, TFA/H_2O/TIPS = 95:2.5:2.5), cold diethyl ether precipitation, HPLC purification (20-70% CH_3CN/H_2O over 30 min) and lyophilization, the desired peptide **S4** was obtained as a white powder (242 mg, 31%).

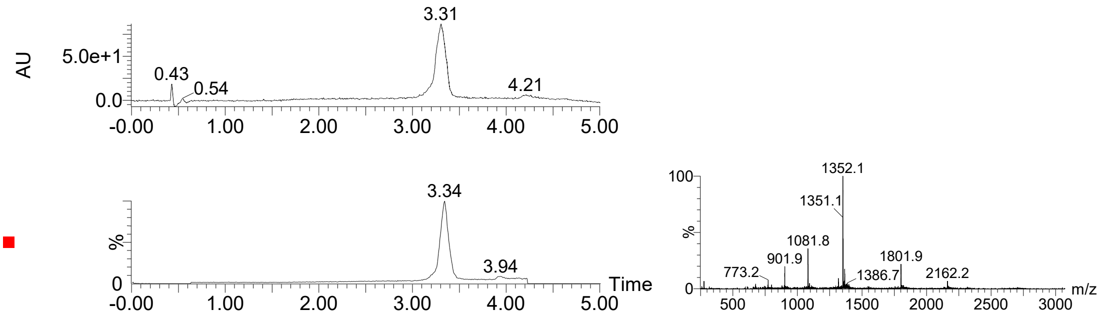

Figure S10. UPLC-MS analysis of purified peptide **S4**. Left: UV (190-400 nm) and MS (300-3000 m/z) trace from UPLC-MS analysis of purified **S4**, gradient 5-95% CH_3CN/H_2O containing 0.1% TFA over 5 min at a flow rate of 0.4 mL/min; Right: ESI-MS calcd. for $C_{240}H_{353}N_{69}O_{69}S_3$: $[M+3H]^{3+}$ *m/z* = 1802.6, found 1801.9; $[M+4H]^{4+}$ *m/z* = 1352.2, found 1352.1; $[M+5H]^{5+}$ *m/z* = 1082.0, found 1081.8; $[M+6H]^{6+}$ *m/z* = 901.8, found 901.9.

4.4 Peptide assembly via STL

4.4.1 Synthesis of S5

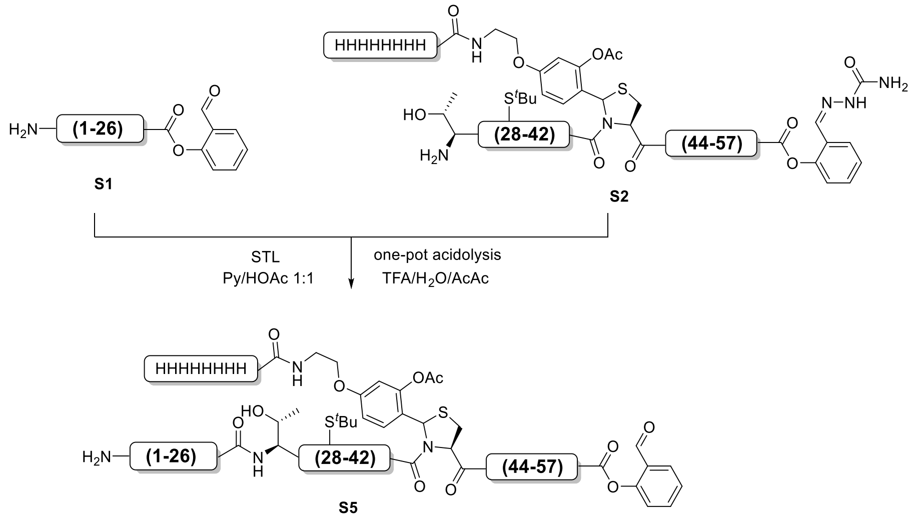

Peptide **S1** (21.6 mg, 6.8 μmol, 1.0 equiv) and peptide **S2** (40.5 mg, 8.1 μmol, 1.2 equiv) were dissolved in pyridine/HOAc (1/1, v/v) cocktail at a concentration of 10 mM under room temperature. The reaction mixture was stirred at room temperature for 2 h. After completion of the reaction indicated by UPLC, the solution was poured into cold diethyl ether to precipitate the peptide. After centrifugation the ether was decanted, and the peptide residue was treated with 5.0 mL of $TFA/H_2O/AcAc$ (95/2.5/2.5) for 2h to

generate a native amide bond as well as to remove the semicarbazone that caped the C-terminal SAL ester. After completion of the ligated peptide acidolysis and SAL ester "off-to-on", 20 mL cold diethyl ether was added to give a white suspension for centrifugation.

After decanting diethyl ether, the remaining solid was dissolved by 8.0 mL 30% CH_3CN /H_2O, and it was filtrated by Syringe Filters (PTFE 0.22μm) and subjected to preparative HPLC purification (20-70% CH_3CN/H_2O over 30 min) and lyophilization to give 12.0 mg (22% yield) of peptide **S5** as a white powder.

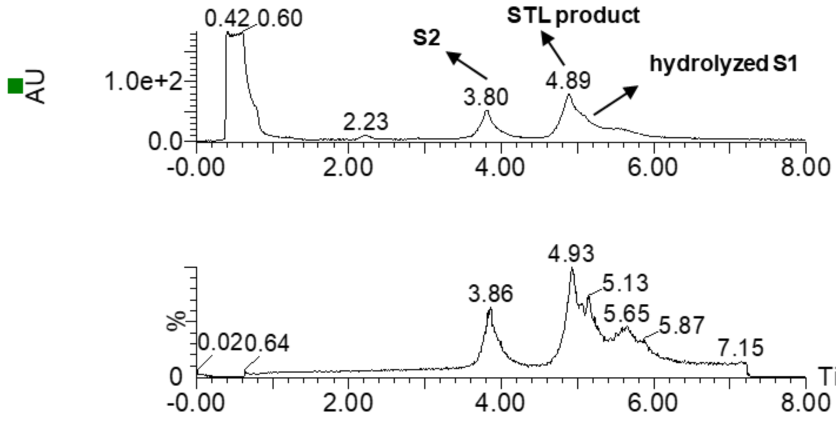

Figure S11. UV (190-400 nm) and MS (300-3000 m/z) trace from UPLC-MS analysis of STL between **S1** and **S2**, gradient 20-70% CH_3CN/H_2O containing 0.1% TFA over 8 min at a flow rate of 0.4 mL/min.

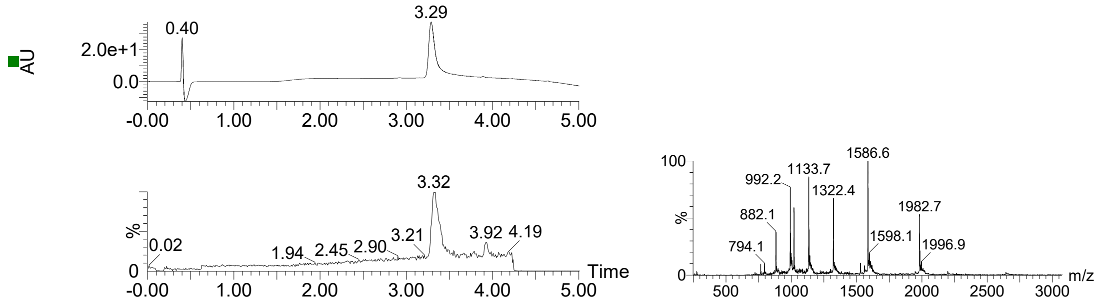

Figure S12. UPLC-MS analysis of purified peptide **S5** after one-pot STL/acidolysis (slightly overlap with inactive hydrolyzed **S1**. Left: UV (190-400 nm) and MS (300-3000 m/z) trace from UPLC-MS analysis of purified **S5**, gradient 5-95% CH_3CN/H_2O containing 0.1% TFA over 5 min at a flow rate of 0.4 mL/min; Right: ESI-MS calcd. for: $[M+4H]^{4+}$ *m/z* = 1983.5, found 1982.7; $[M+5H]^{5+}$ *m/z* = 1587.0, found 1586.6; $[M+6H]^{6+}$ *m/z* = 1322.6, found 1322.4; $[M+7H]^{7+}$ *m/z* =

1133.8, found 1133.7; [M+8H]$^{8+}$ m/z = 992.2, found 992.2; [M+9H]$^{9+}$ m/z = 882.1, found 882.1; [M+10H]$^{10+}$ m/z = 794.0, found 794.1.

4.4.2 Synthesis of S6

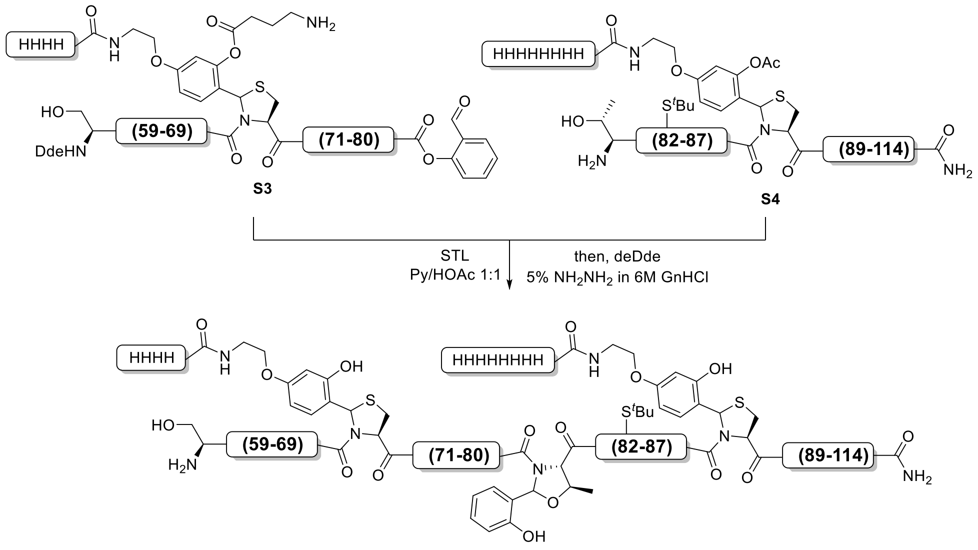

S6

Peptide **S3** (28 mg, 7.8 μmol, 1.0 equiv) and peptide **S4** (51 mg, 9.4 μmol, 1.2 equiv) were dissolved in pyridine/HOAc (1/1, v/v) cocktail at a concentration of 10 mM under room temperature. The reaction mixture was stirred at room temperature for 3 h. After completion of the reaction indicated by UPLC, the solution was poured into cold diethyl ether to precipitate the peptide. After centrifugation the ether was decanted, and the peptide residue was treated with 5.0 mL 5% NH_2NH_2 in CH_3CN/H_2O 50/50 for 30min to deprotect Dde. After completion of the reaction, the pH was adjusted to around 5.0 using 6 N HCl. The solution was filtrated by Syringe Filters (PTFE 0.22μm) and subjected to preparative HPLC purification (20-70% CH_3CN/H_2O over 30 min) and lyophilization to give 25 mg (37% yield) of peptide **S6** as a white powder.

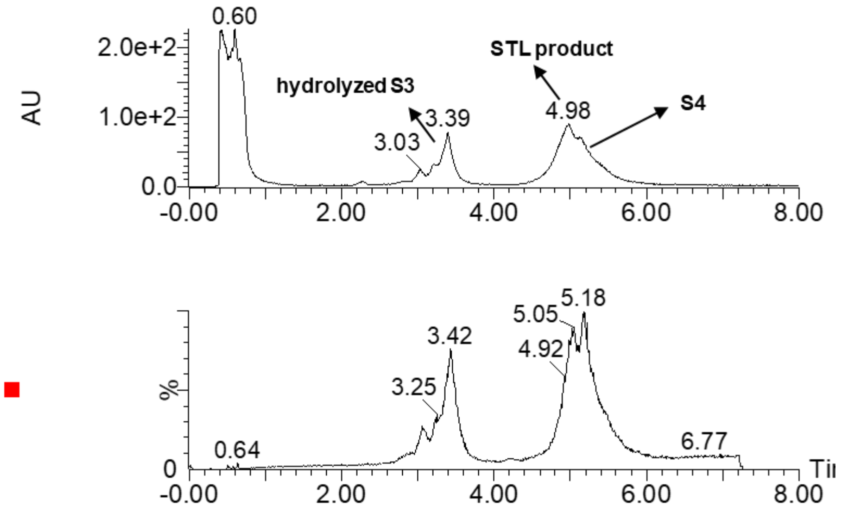

Figure S13. UV (190-400 nm) and MS (300-3000 m/z) trace from UPLC-MS analysis of STL between **S3** and **S4**, gradient 20-70% CH_3CN/H_2O containing 0.1% TFA over 8 min at a flow rate of 0.4 mL/min.

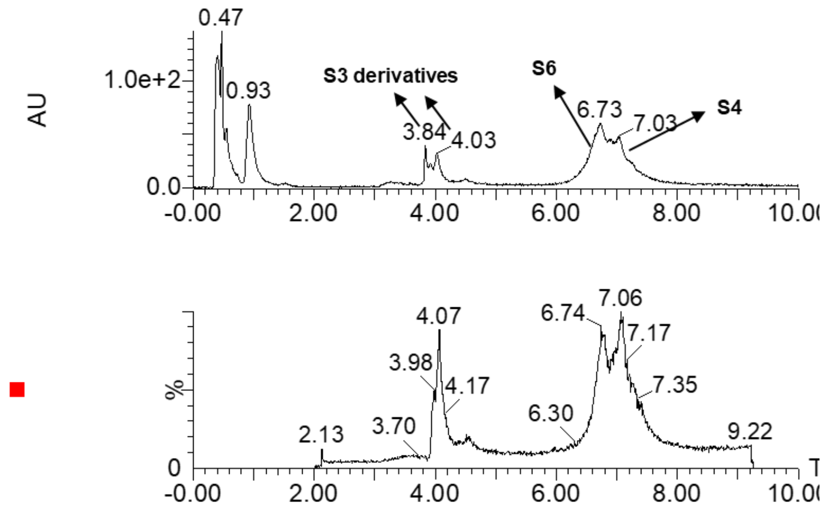

Figure S14. UV (190-400 nm) and MS (300-3000 m/z) trace from UPLC-MS analysis of One-pot Dde removal after STL between **S3** and **S4**, gradient 20-70% CH_3CN/H_2O containing 0.1% TFA over 8 min at a flow rate of 0.4 mL/min.

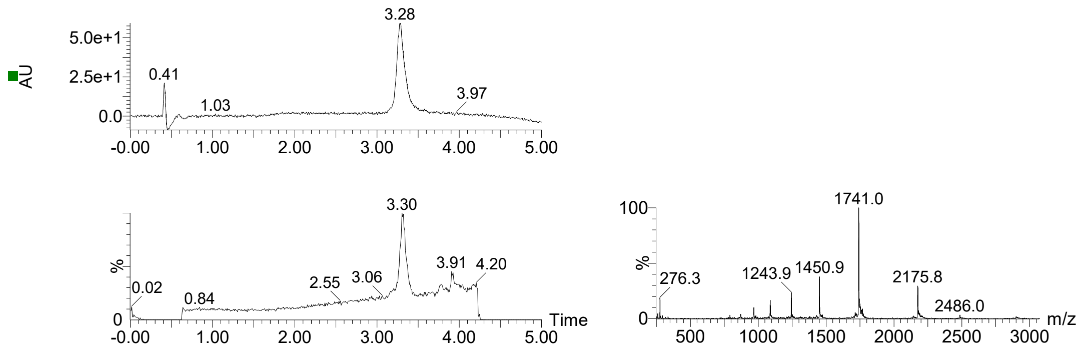

Figure S15. UPLC-MS analysis of purified peptide **S6**. Left: UV (190-400 nm) and MS (300-3000 m/z) trace from UPLC-MS analysis of **S6**, gradient 5-95% CH_3CN/H_2O containing 0.1% TFA over 5 min at a flow rate of 0.4 mL/min; Right: ESI-MS calcd. for $C_{384}H_{567}N_{113}O_{113}S_4$: $[M+4H]^{4+}$ *m/z* = 2176.6, found 2175.8; $[M+5H]^{5+}$ *m/z* = 1741.5, found 1741.0; $[M+6H]^{6+}$ *m/z* = 1451.4, found 1450.9; $[M+7H]^{7+}$ *m/z* = 1244.2, found 1243.9; $[M+8H]^{8+}$ *m/z* = 1088.8, found 1088.9; $[M+9H]^{9+}$ *m/z* = 968.0, found 968.2.

4.4.3 Synthesis of S7

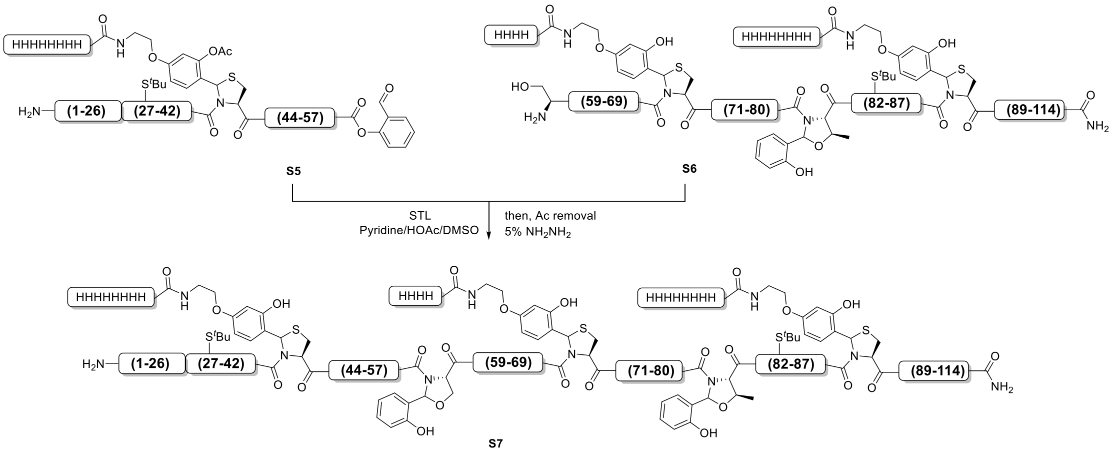

Peptide **S5** (12.3 mg, 1.56 μmol, 1.0 equiv) and peptide **S6** (17.3 mg, 2.03 μmol, 1.3 equiv) were dissolved in Pyridine/HOAc/DMSO (1/1/0.2, v/v/v) cocktail at a concentration of 5 mM under room temperature. The reaction mixture was stirred at room temperature for 4 h. After completion of the reaction indicated by UPLC, the solution was poured into cold diethyl ether to precipitate the peptide. After centrifugation the ether was decanted, and the peptide residue was treated with 1.0 mL 5% NH_2NH_2 in 6 M GnHCl for 30min to remove Ac. After completion of the reaction, the pH was adjusted to around 5.0 using 6 N HCl. The solution was filtrated by Syringe

Filters (PTFE 0.22μm) and subjected to preparative HPLC purification (20-70% CH_3CN/H_2O over 30 min) and lyophilization to give 4.2 mg (16% yield) of peptide **S7** as a white powder.

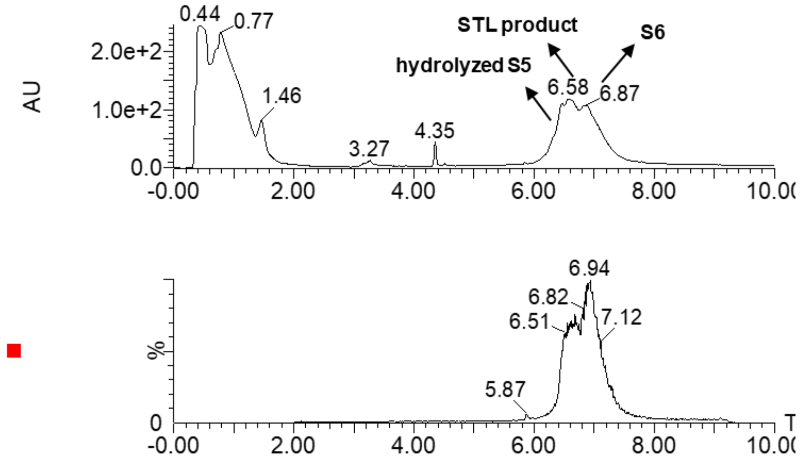

Figure S16. UV (190-400 nm) and MS (300-3000 m/z) trace from UPLC-MS analysis of STL between **S5** and **S6**, gradient 20-70% CH_3CN/H_2O containing 0.1% TFA over 10 min at a flow rate of 0.4 mL/min.

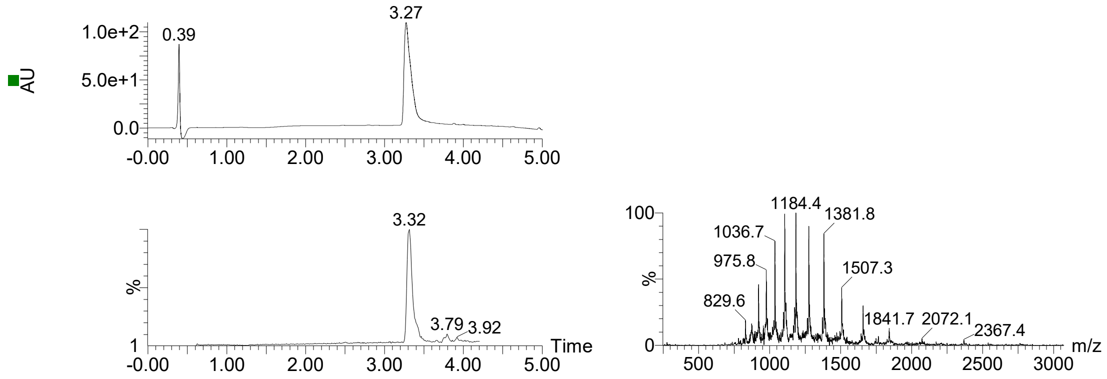

Figure S17. UPLC-MS analysis of purified peptide **S7**. Left: UV (190-400 nm) and MS (300-3000 m/z) trace from UPLC-MS analysis of **S7**, gradient 5-95% CH_3CN/H_2O containing 0.1% TFA over 5 min at a flow rate of 0.4 mL/min; Right: ESI-MS calcd. for $C_{736}H_{1098}N_{208}O_{214}S_9$: $[M+7H]^{7+}$ *m/z* = 2368.5, found 2367.4; $[M+8H]^{8+}$ *m/z* = 2072.5, found 2072.1; $[M+9H]^{9+}$ *m/z* = 1842.4, found 1841.7; $[M+10H]^{10+}$ *m/z* = 1658.2, found 1658.1; $[M+11H]^{11+}$ *m/z* = 1507.6, found 11507.3; $[M+12H]^{12+}$ *m/z* = 1382.0, found 1381.8; $[M+13H]^{13+}$ *m/z* = 1275.8, found 1275.4; $[M+14H]^{14+}$ *m/z* = 1184.7, found 1184.4; $[M+15H]^{15+}$ *m/z* = 1105.8, found 1105.7; $[M+16H]^{16+}$ *m/z* = 1036.8, found 1036.7; $[M+17H]^{17+}$ *m/z* = 975.8, found 975.8; $[M+18H]^{18+}$ *m/z* = 921.7, found 921.5; $[M+20H]^{20+}$ *m/z* = 829.6, found 829.6.

4.4.4 Solubility problem of S8

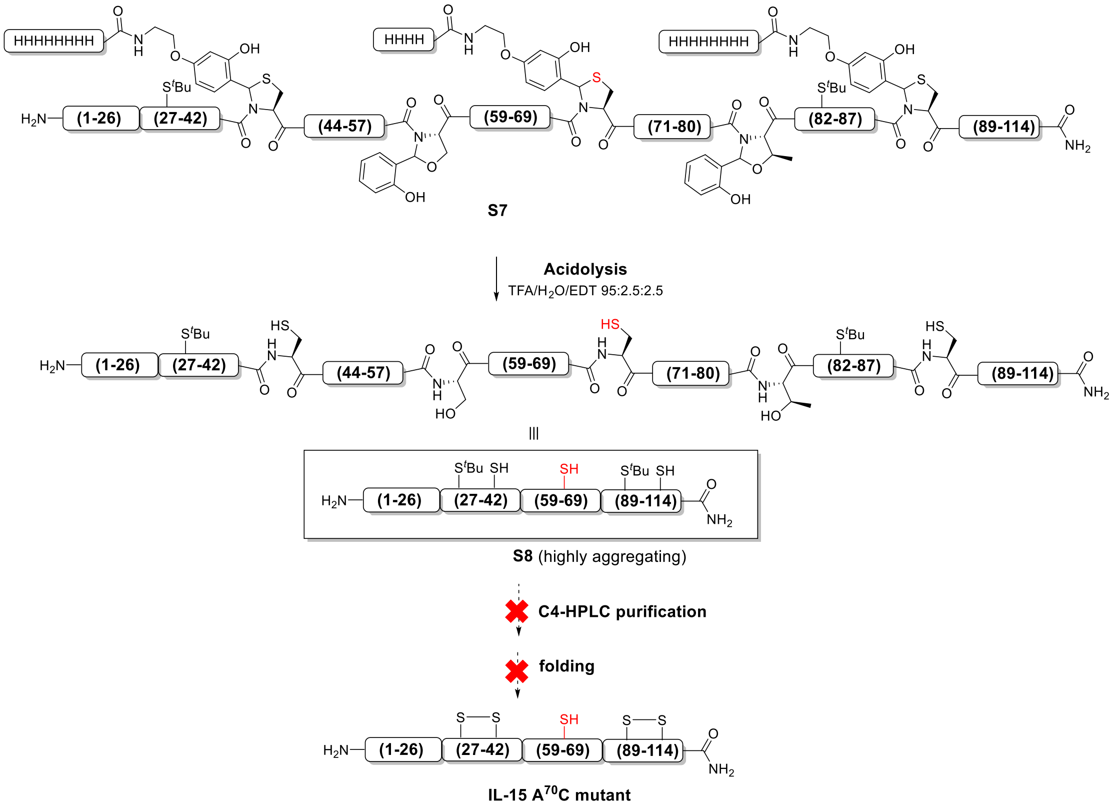

After obtaining full-length IL-15 (**S7**) with several backbone modifications, we tried to perform acidolysis to give linear IL-15. Peptide **S7** (5.2 mg) was dissolved in 3 mL TFA/H_2O/EDT cocktail (95:2.5:2.5) and stirred for 1.5 h. Subsequently, TFA was blown off under a stream of compressed N_2 until there was less than 0.2 mL solvent. The residue was diluted with 5 mL 50% CH_3CN/H_2O. However, all the protein precipitated from the solution. After centrifugation and decanting the liquid phase, the solid was suspended in 8 M GnHCl (pH 6.0 or pH 8.0) for 3 hours and checked by UPLC-MS. Yet no protein was detected both in CH_3CN/H_2O solution or 6M GnHCl suspension.

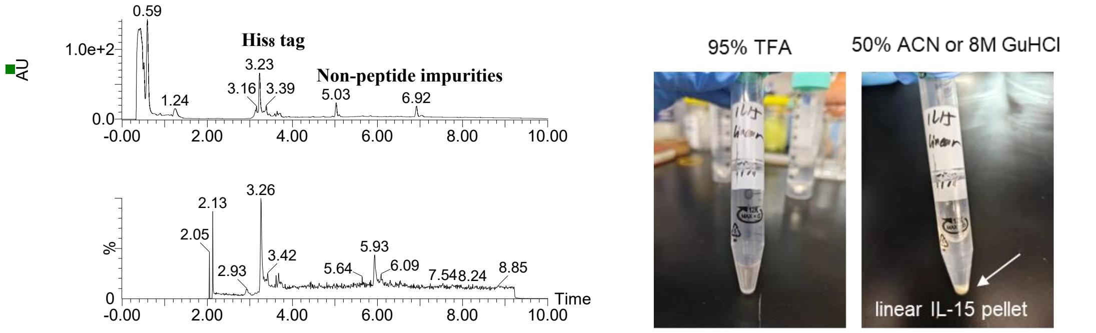

Figure S18. Left: UV (190-400 nm) and MS (300-3000 m/z) trace from UPLC-MS analysis of

precipitate protein suspension in 6M GnHCl after acidolysis of **S7**. Right: Solubility of linear IL-15 is very poor in 50% ACN/H_2O acidic solution and cannot be solubilized again in 50% ACN/H_2O or 8M GnHCl after aggregation and centrifugation. Once forming protein pellet, IL-15 rarely goes back to solution phase, because the temperature that requires to solubilize IL-15 can break peptide bond or lead to side reaction (such as Asn^{77} deamidation).

One-pot third STL/deAc/Acidolysis protocol allowed S8 to be observed:

Peptide **S5** (12.3 mg, 1.56 μmol, 1.0 equiv) and peptide **S6** (17.3 mg, 2.03 μmol, 1.3 equiv) were dissolved in Pyridine/HOAc/DMSO (1/1/0.2, v/v/v) cocktail at a concentration of 5 mM under room temperature. The reaction mixture was stirred at room temperature for 4 h. The solution was poured into cold diethyl ether to precipitate the peptide. After centrifugation the ether was decanted, and the peptide residue was treated with 1.0 mL 2% NH_2NH_2 in 50% ACN and all solvent was removed by lyophilization. TFA/H_2O/EDT (95:2.5:2.5) cocktail was added. After 2h, the peptide solution was diluted, lyophilized and redissolved in 8M GnHCl. This time **S8** was not fully aggregated in the mixture with peptide impurities so it could be observed in UPLC-MS.

However, the protein was not eluted from C4 column during HPLC purification because of linear IL-15 tended to aggregate in the column.

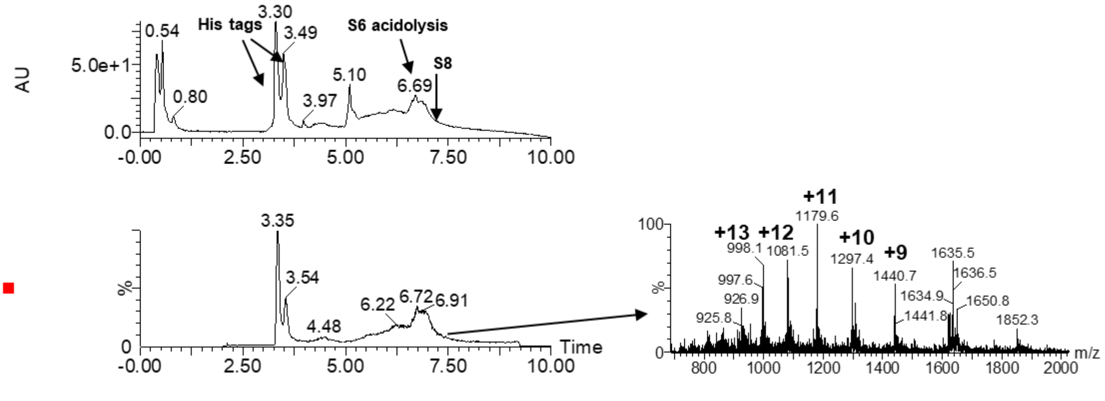

Figure S19. UV (190-400 nm) and MS (300-3000 m/z) trace from UPLC-MS analysis of precipitate protein suspension in 6M GnHCl after one-pot STL/acidolysis of **S7** to afford **S8** (left), gradient 20-70% CH_3CN/H_2O containing 0.1% TFA over 10 min at a flow rate of 0.4 mL/min. This time peptide **S8** was observed in ESI-MS (right), cald. for $C_{567}H_{912}N_{144}O_{183}S_9$: $[M+7H]^{7+}$ *m/z* =1852.8, found 1852.3; $[M+8H]^{8+}$ *m/z* =1621.4, found 1620.7; $[M+9H]^{9+}$ *m/z* =1441.3, found 1440.7; $[M+10H]^{10+}$ *m/z* =1297.3, found 1297.4; $[M+11H]^{11+}$ *m/z* =1179.4, found 1179.6; $[M+12H]^{12+}$ *m/z* =1081.2, found 1081.5; $[M+13H]^{13+}$ *m/z* =998.1, found 998.1.

4.5 Selective removal of TBM for Cys^{70} desulfurization

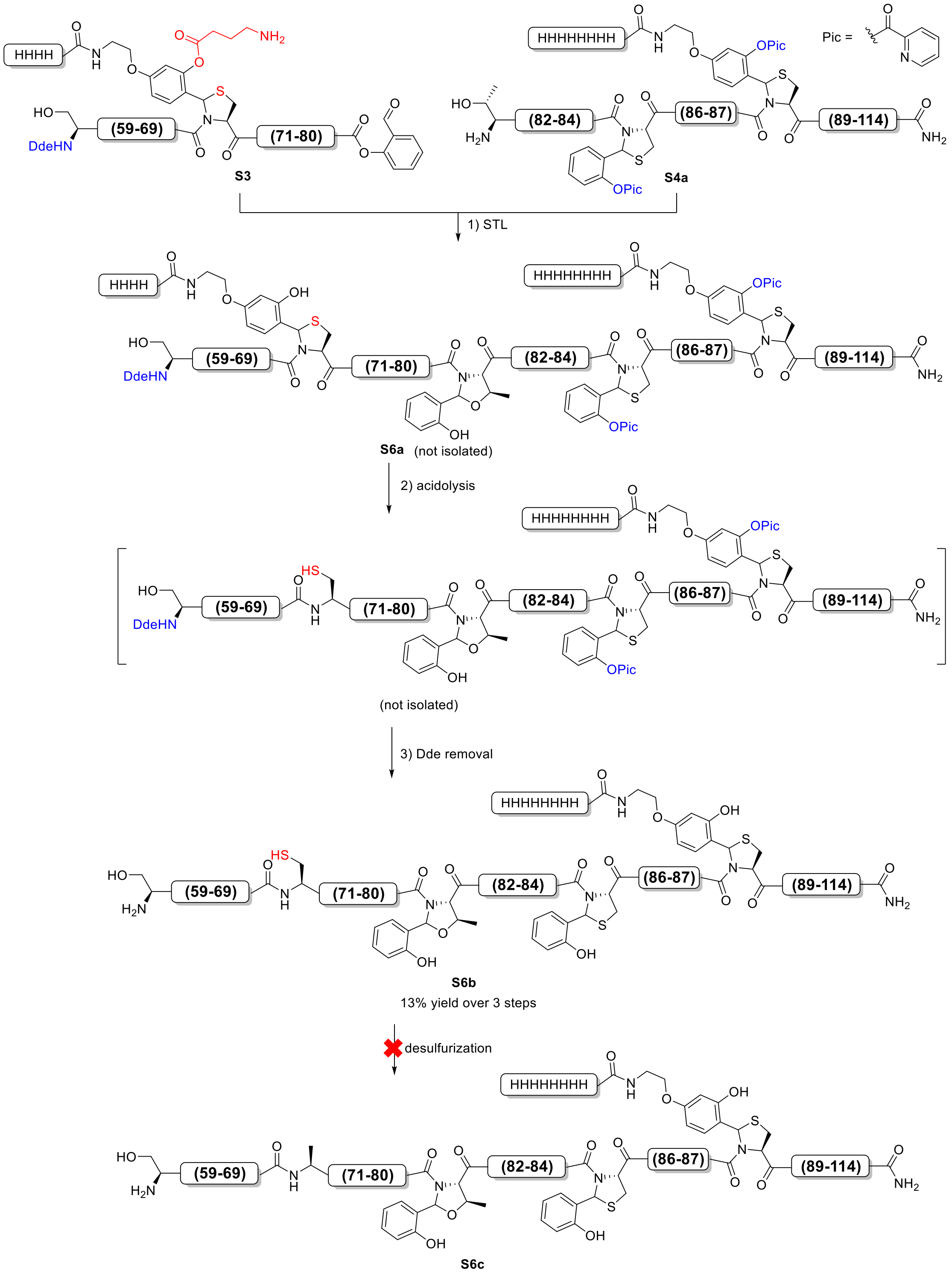

Scheme S3. Attempts to the synthesis of **S6c** via STL, acidolysis, Dde removal followed by desulfurization

4.5.1 Synthesis of S4a

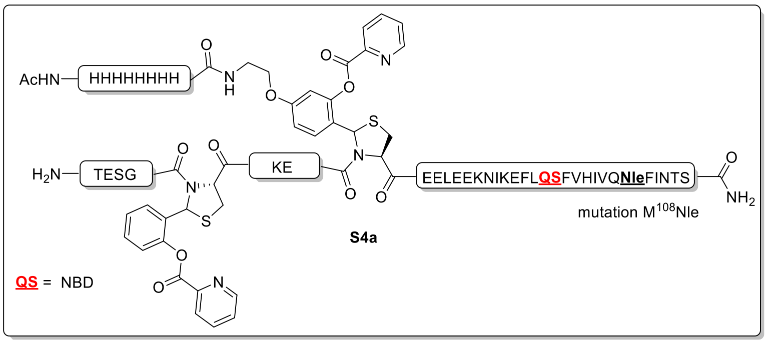

Peptide **S4a** was synthesized by general procedure **2.1** using 200 mg Rink Amide AM resin (loading 0.5 mmol/g). EC-Alloc and GC-OMe dipeptides were synthesized by procedure **2.6** for installation of TBM. Met^{108} was mutated to Nle^{108}. Subsequently, 2-Pic-capping was conducted. After TFA global deprotection (30 mL, TFA/H_2O/TIPS = 95:2.5:2.5), cold diethyl ether precipitation, HPLC purification (20-70% CH_3CN/H_2O over 30 min) and lyophilization, the desired peptide **S4a** was obtained as a white powder (42 mg, 7.5%).

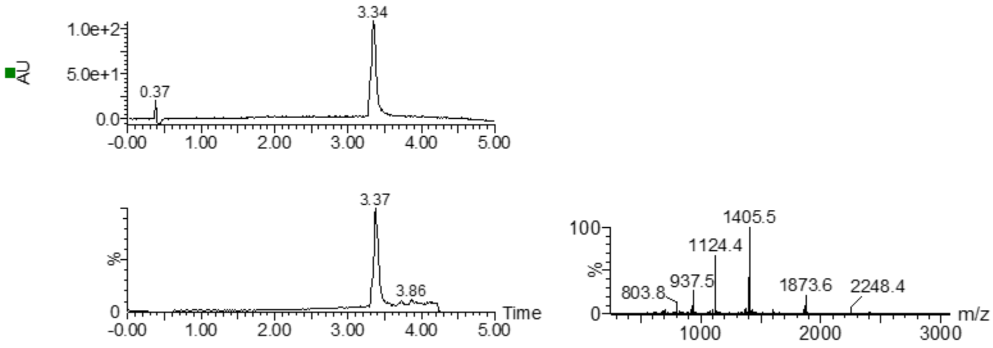

Figure S20. UPLC-MS analysis of purified peptide **S4a**. Left: UV (190-400 nm) and MS (300-3000 m/z) trace from UPLC-MS analysis of **S4a**, gradient 5-95% CH_3CN/H_2O containing 0.1% TFA over 5 min at a flow rate of 0.4 mL/min; Right: ESI-MS cald. for $C_{254}H_{355}N_{71}O_{72}S_2$: $[M+3H]^{3+}$ *m/z* =1874.1, found 1873.6; $[M+4H]^{4+}$ *m/z* =1405.8, found 1405.5; $[M+5H]^{5+}$ *m/z* =1124.8, found 1124.4; $[M+6H]^{6+}$ *m/z* =937.5, found 937.5; $[M+7H]^{7+}$ *m/z* =803.7, found 803.8.

4.5.2 Synthesis of S6b via one-pot STL/acidolysis/Dde removal

Peptide **S3** (19.4 mg, 5.4 μmol, 1.0 equiv) and peptide **S4a** (39.4 mg, 7.03 μmol, 1.3 equiv) were dissolved in Pyridine/HOAc/DMSO (1/1/0.2, v/v/v) cocktail at a concentration of 5 mM under room temperature. The reaction mixture was stirred at

room temperature for 4 h. After completion of the reaction indicated by UPLC, the solution was poured into cold diethyl ether to precipitate the peptide. After centrifugation the ether was decanted, and residual peptide was dissolved in 6 mL TFA/H_2O/EDT (95:2.5:2.5) cocktail. After 2h, 40 mL cold ether was added. After centrifugation the ether was decanted, and the peptide residue was treated with 5.0 mL 2% NH_2NH_2 in 6 M GnHCl for 30min to remove Dde and 2-Pic. Then, the pH was adjusted to around 5.0 using 6 N HCl. The solution was filtrated by Syringe Filters (PTFE 0.22μm) and subjected to preparative HPLC purification (20-70% CH_3CN/H_2O over 30 min) and lyophilization to give 5.5 mg (13% yield over three steps) peptide **S6b** as a white powder.

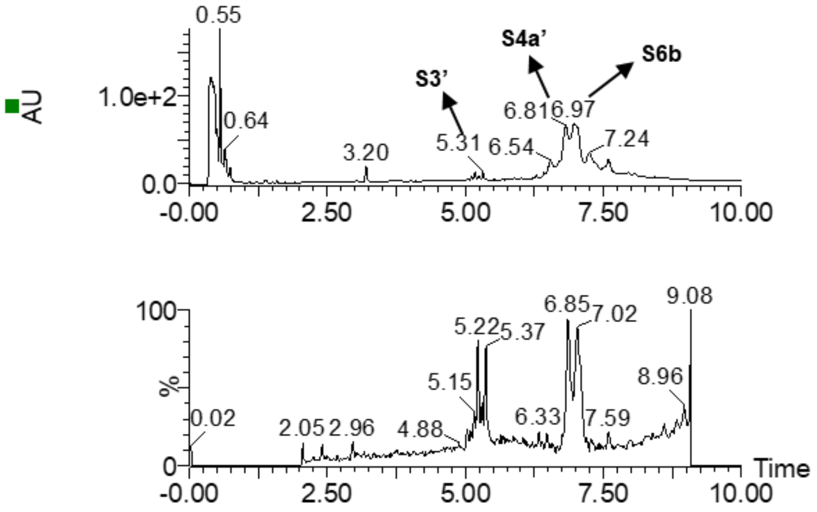

Figure S21. UV (190-400 nm) and MS (300-3000 m/z) trace from UPLC-MS analysis of One-pot STL/acidolysis/Dde removal, gradient 20-70% CH_3CN/H_2O containing 0.1% TFA over 10 min at a flow rate of 0.4 mL/min. **S3'** was hydrolysis product of **S3**, and **S4a'** was acidolysis product of **S4**.

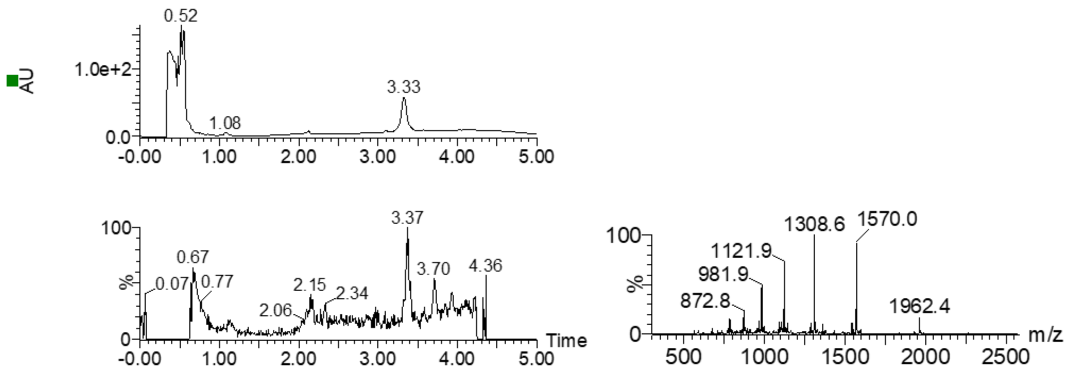

Figure S22. UPLC-MS analysis of purified peptide **S6b**. Left: UV (190-400 nm) and MS (300-3000 m/z) trace from UPLC-MS analysis of **S6b**, gradient 5-95% CH_3CN/H_2O containing 0.1% TFA over

5 min at a flow rate of 0.4 mL/min; Right: ESI-MS Calcd. for $C_{342}H_{521}N_{99}O_{106}S_4$: $[M+4H]^{4+}$ *m/z* =1961.9, found1962.4; $[M+5H]^{5+}$ *m/z* =1569.8, found 1570.0; $[M+6H]^{6+}$ *m/z* =1308.3, found 1308.6; $[M+7H]^{7+}$ *m/z* =1121.5, found 1121.9; $[M+8H]^{8+}$ *m/z* =981.5, found 981.9; $[M+9H]^{9+}$ *m/z* =872.5, found 872.8.

During desulfurization of **S6b** using $NaBEt_4$, peptide precipitated from the solution after adding $NaBEt_4$, and no starting material or desulfurization product was observed in the UPLC-MS spectrum.

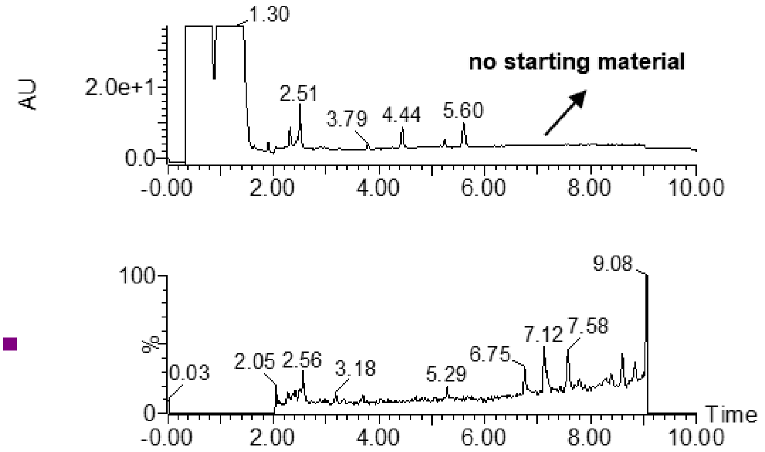

Figure S23. UV (190-400 nm) and MS (300-3000 m/z) trace from UPLC-MS analysis of desulfurization of **S6b** using $NaBEt_4$ as radical initiator (Add-and-Done Desulfurization) after 20h reaction, gradient 20-70% CH_3CN/H_2O containing 0.1% TFA over 10 min at a flow rate of 0.4 mL/min.

During desulfurization of **S6b** using VA044, peptide signal gradually disappeared in the UPLC-MS spectrum, generating some new small peptide signals. We did not do further optimization for this desulfurization because we encountered a more severe challenge that the 3rd STL between **S5 and S6b** did not work (see **4.7**) without TBM on **Cys[70]**.

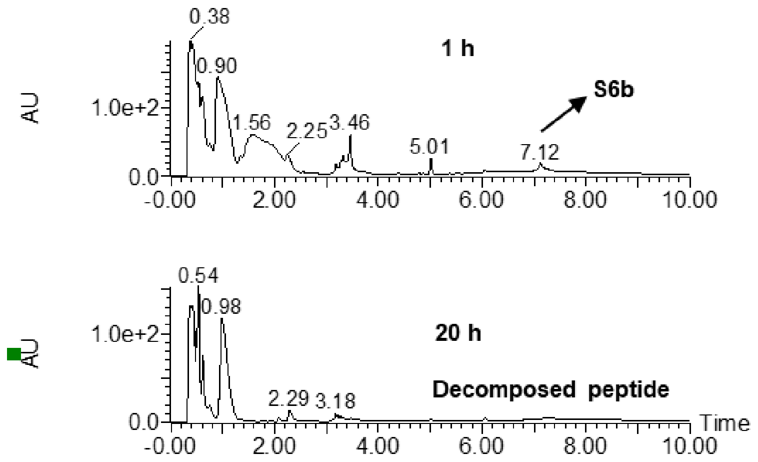

Figure S24. UV (190-400 nm) and MS (300-3000 m/z) trace from UPLC-MS analysis of desulfurization of **S6b** using VA044 as radical initiator after 1h (up) or 20h (down) reaction, gradient 20-70% CH_3CN/H_2O containing 0.1% TFA over 10 min at a flow rate of 0.4 mL/min.

4.6 Combination of RST-2.0 and TBM for site-specific desulfurization

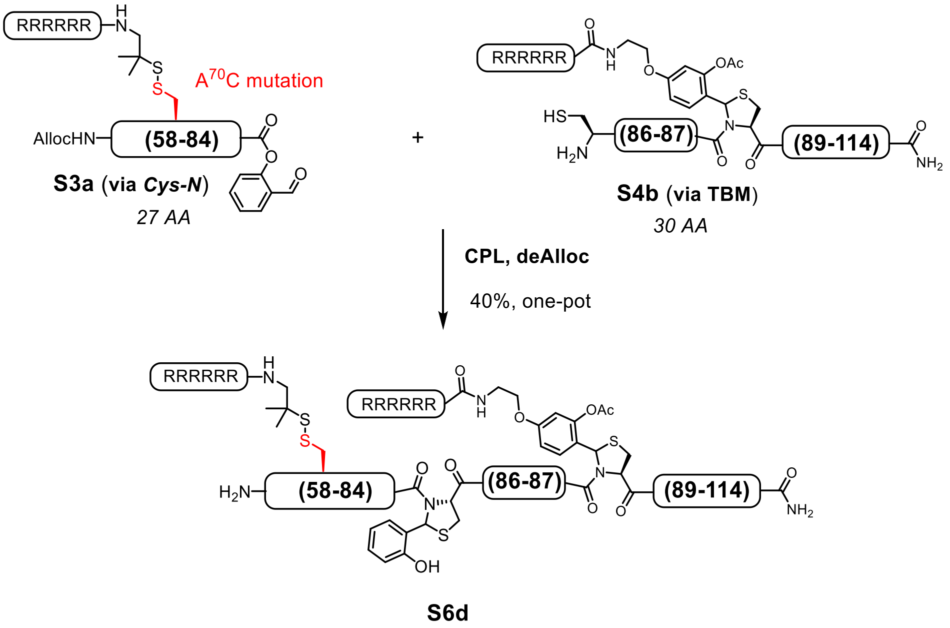

Scheme S4. Synthesis of **S6d** via CPL and Alloc removal in one pot.

4.6.1 Synthesis of S3a

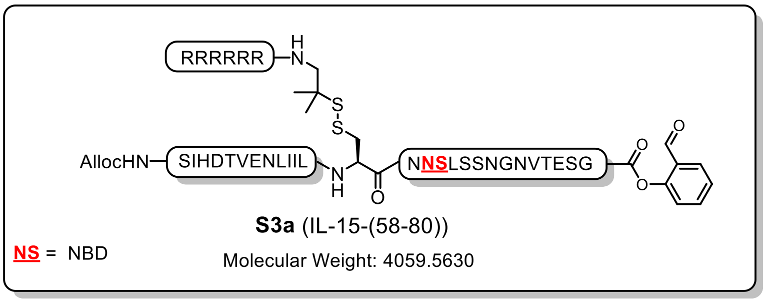

Peptide **S3a** was synthesized by general procedure **2.1** and **2.2** (**Cys-N**, method A) using 200 mg 2-chlorotrityl chloride resin (loading 0.5 mmol/g). Subsequently, following procedure **2.3**, **2.5**, TFA global deprotection (30 mL, TFA/H_2O/Pyruvic acid = 95:2.5:2.5), cold diethyl ether precipitation, HPLC purification (15-60% CH_3CN/H_2O over 30 min) and lyophilization, the desired peptide SAL ester was obtained as a white

powder (93 mg, 23%).

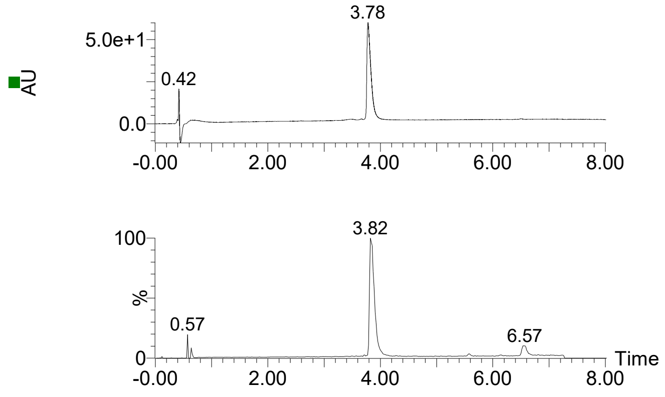

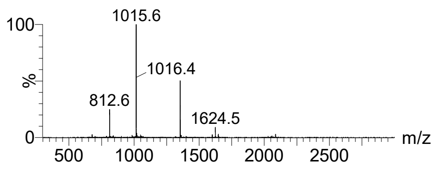

Figure S25. UPLC-MS analysis of purified peptide **S3a**. Left: UV (190-400 nm) and MS (300-3000 m/z) trace from UPLC-MS analysis of **S3a**, gradient 15-60% CH_3CN/H_2O containing 0.1% TFA over 8 min at a flow rate of 0.4 mL/min; Right: ESI-MS cald. for $C_{167}H_{281}N_{59}O_{55}S_2$: $[M+3H]^{3+}$ *m/z* =1354.2, found 1354.0; $[M+4H]^{4+}$ *m/z* =1015.9, found 1015.6; $[M+5H]^{5+}$ *m/z* =812.9, found 812.6.

4.6.2 Synthesis of S4b

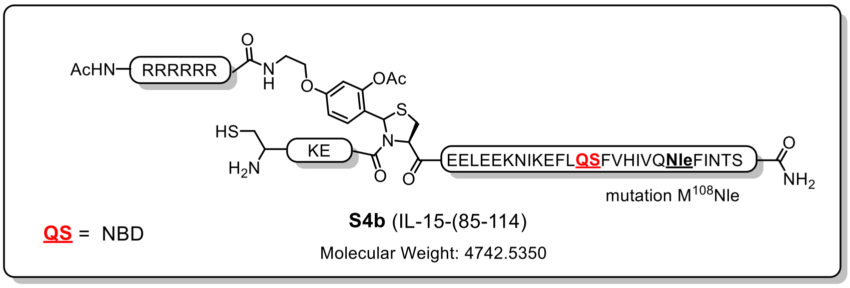

Peptide **S4b** was synthesized by general procedure **2.1** using 200 mg Rink Amide AM resin (loading 0.5 mmol/g). EC-Alloc dipeptide was synthesized by procedure **2.6** for installation of TBM. Met^{108} was mutated to Nle^{108}. Subsequently, Ac-capping was conducted by treating with Ac_2O/DIEA/DCM (2:4:100 v/v/v) cocktail. After TFA global deprotection (30 mL, TFA/H_2O/TIPS = 95:2.5:2.5), cold diethyl ether precipitation, HPLC purification (20-70% CH_3CN/H_2O over 30 min) and lyophilization, the desired peptide **S4b** was obtained as a white powder (169 mg, 33%).

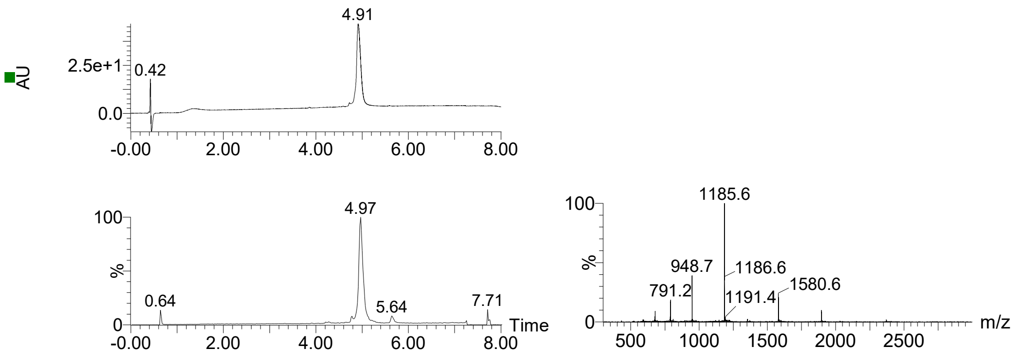

Figure S26. UPLC-MS analysis of purified peptide **S4b**. Left: UV (190-400 nm) and MS (300-3000 m/z) trace from UPLC-MS analysis of **S4b**, gradient 20-70% CH_3CN/H_2O containing 0.1% TFA over 8 min at a flow rate of 0.4 mL/min; Right: ESI-MS cald. for $C_{207}H_{335}N_{65}O_{57}S_3$: $[M+3H]^{3+}$ *m/z* =1581.8, found 1580.6; $[M+4H]^{4+}$ *m/z* =1186.6, found 1185.6; $[M+5H]^{5+}$ *m/z* =949.5, found 948.7; $[M+6H]^{6+}$ *m/z* =791.4, found 791.2.

4.6.2 Synthesis of S6d by Cysteine/Penicillamine ligation (CPL)

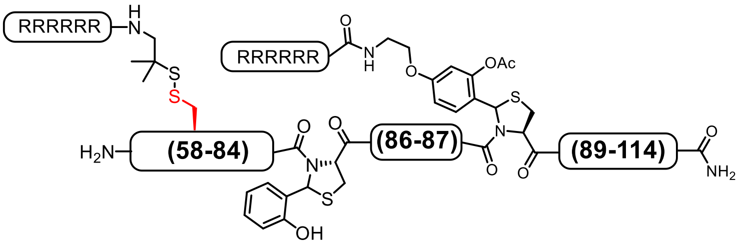

S6d

Peptide **S3** (6.1 mg, 1.3 equiv) and peptide **S4b** (5.5 mg, 1.0 equiv) were dissolved in Pyridine/HOAc (1/1, v/v) cocktail at a concentration of 5 mM under room temperature. The reaction mixture was stirred at room temperature for 2 h. After completion of the reaction indicated by UPLC, the solution was poured into cold diethyl ether to precipitate the peptide. After centrifugation the ether was decanted, and residual peptide was dissolved by 1 mL HOAc containing 10 mg $Pd(PPh_3)_4$ and 40 mg 1,3-dimethyl barbituric acid and stirred vigorously for 1h. Reaction was monitored by UPLC-MS. After completion, the solution was filtrated by Syringe Filters (PTFE 0.22μm) and subjected to preparative HPLC purification (20-70% CH_3CN/H_2O over 30 min) and lyophilization to give 4.0 mg peptide **S6d** (40% yield, one-pot) as a white powder.

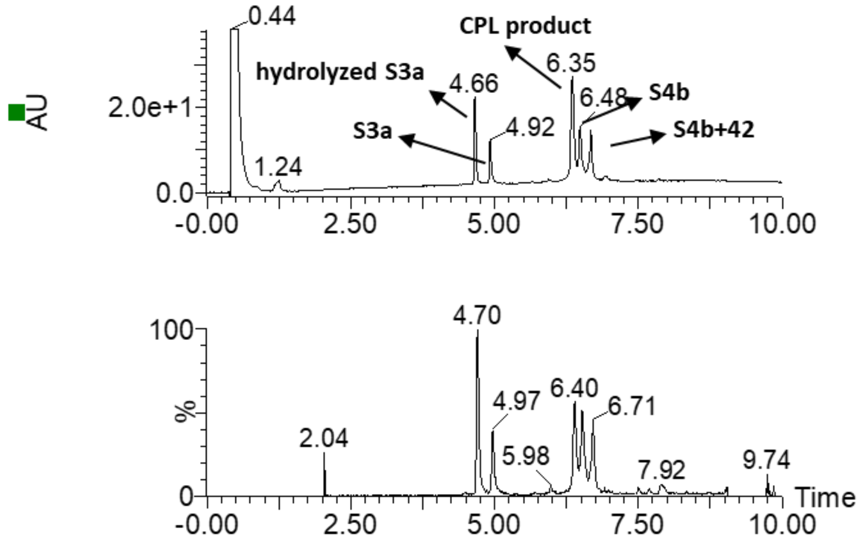

Figure S27. UV (190-400 nm) and MS (300-3000 m/z) trace from UPLC-MS analysis of CPL between **S3a** and **S4b**, gradient 20-70% CH_3CN/H_2O containing 0.1% TFA over 10 min at a flow rate of 0.4 mL/min.

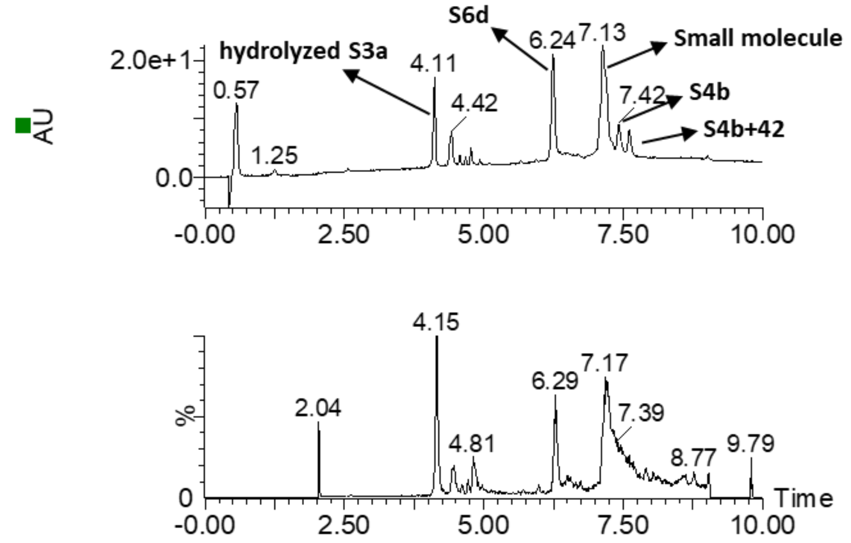

Figure S28. UV (190-400 nm) and MS (300-3000 m/z) trace from UPLC-MS analysis of one-pot Alloc removal after CPL between **S3a** and **S4b**, gradient 20-70% CH_3CN/H_2O containing 0.1% TFA over 10 min at a flow rate of 0.4 mL/min.

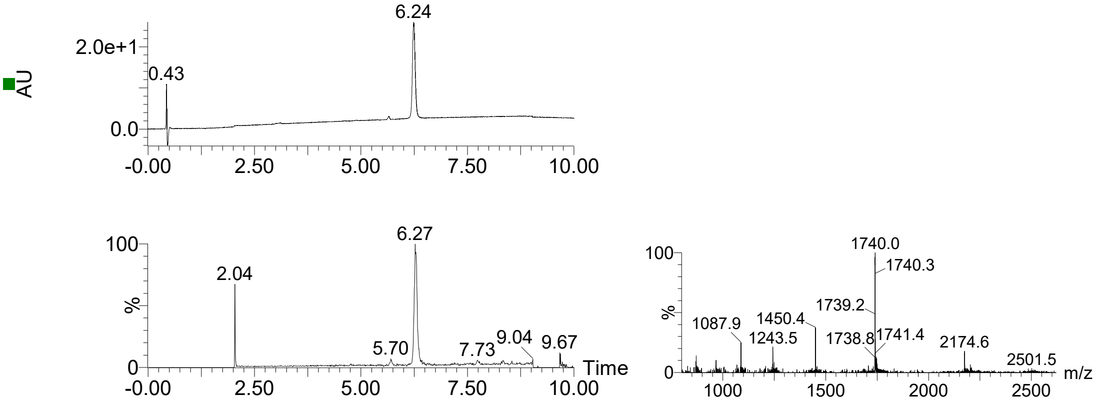

Figure S29. UPLC-MS analysis of purified peptide **S6d**. Left: UV (190-400 nm) and MS (300-3000 m/z) trace from UPLC-MS analysis of **S6d**, gradient 20-70% CH_3CN/H_2O containing 0.1% TFA over 10 min at a flow rate of 0.4 mL/min; Right: ESI-MS cald. for $C_{370}H_{610}N_{124}O_{109}S_5$ (Molecular Weight: 8700.0090): $[M+4H]^{4+}$ *m/z* =2176.0, found 2174.6; $[M+5H]^{5+}$ *m/z* =1741.0, found 1740.0; $[M+6H]^{6+}$ *m/z* =1451.0, found 1450.4; $[M+7H]^{7+}$ *m/z* =1243.8, found 1243.5; $[M+8H]^{8+}$ *m/z* =1088.5, found 1087.9.

4.7 STL problem between S5 and S6b

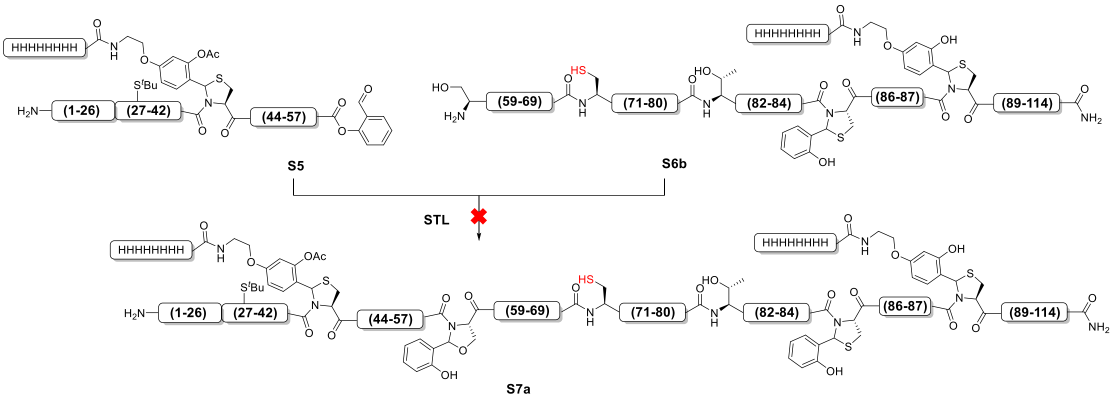

S5 (7 mg) and **S6b** (9.8 mg) were dissolved in Pyridine/HOAc/DMSO (1/1/0.2, v/v/v) cocktail at a concentration of 5 mM under room temperature. The reaction mixture was stirred at room temperature for 4 h. However, only **S5** hydrolysis side product was observed, and no ligation product was found in UPLC-MS spectra.

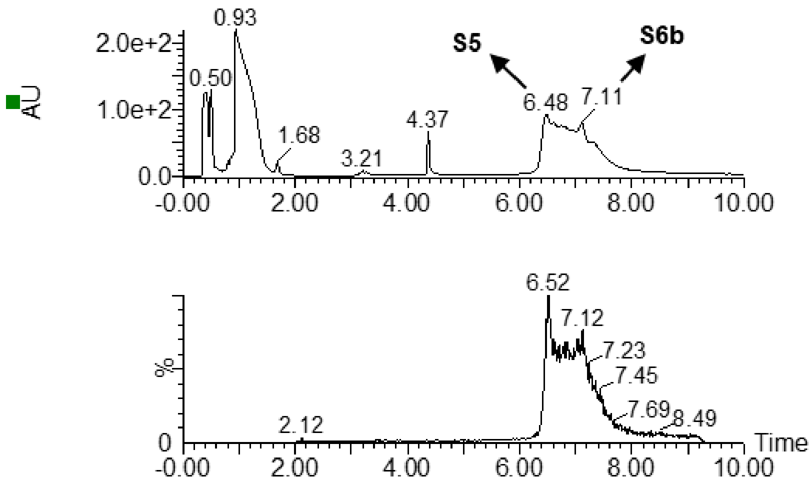

Figure S30. UV (190-400 nm) and MS (300-3000 m/z) trace from UPLC-MS analysis of STL between **S5** and **S6b** after 4h reaction, no STL product was observed gradient 20-70% CH_3CN/H_2O containing 0.1% TFA over 10 min at a flow rate of 0.4 mL/min.

5. Synthesis of Wild-type and N79-Glycosylated IL-15

5.1 Synthesis of peptide segments

5.1.1 Synthesis of peptide 2

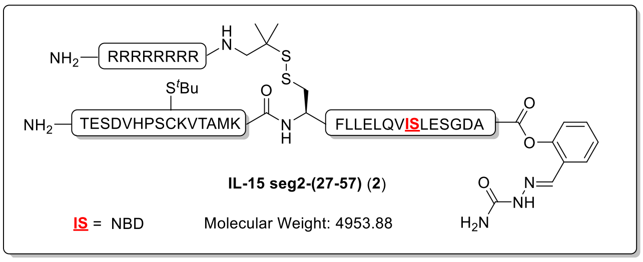

Peptide **2** was synthesized by general procedure **2.1, 2.2** (Method A) using 200 mg 2-chlorotrityl chloride resin (loading 0.5 mmol/g) and Cys-N. Subsequently, following procedure **2.3**, **2.5**, TFA global deprotection (30 mL, TFA/H_2O = 95:5), cold diethyl ether precipitation, HPLC purification (15-60% CH_3CN/H_2O over 30 min) and lyophilization, the desired peptide SAL ester **2** was obtained as a white powder (109 mg, 22%).

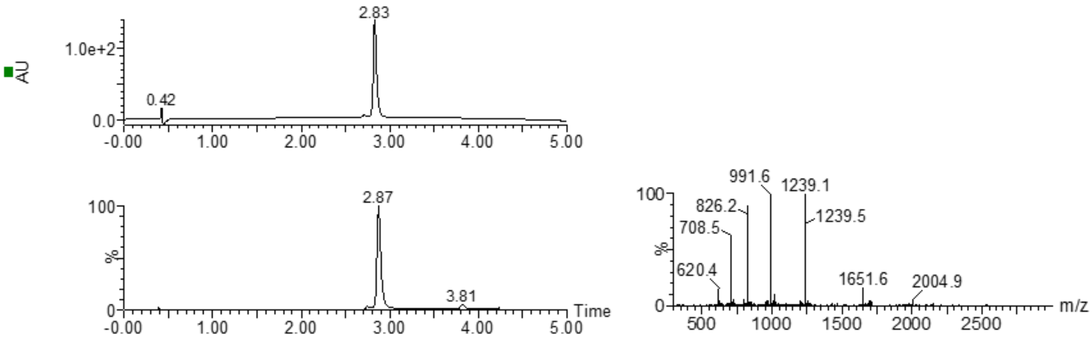

Figure S31. UPLC-MS analysis of purified peptide **2**. Left: UV (190-400 nm) and MS (300-3000 m/z) trace from UPLC-MS analysis of purified **2**, gradient 5-95% CH_3CN/H_2O containing 0.1% TFA over 5 min at a flow rate of 0.4 mL/min; Right: ESI-MS calcd. for $C_{208}H_{356}N_{72}O_{58}S_5$: $[M+3H]^{3+}$ *m/z* = 1652.3, found 1651.6; $[M+4H]^{4+}$ *m/z* = 1239.4, found 1239.1; $[M+5H]^{5+}$ *m/z* = 991.7, found 991.6; $[M+6H]^{6+}$ *m/z* = 826.6, found 826.2; $[M+7H]^{7+}$ *m/z* = 708.7, found 708.5; $[M+8H]^{8+}$ *m/z* = 620.2, found 620.4.

5.1.2 Synthesis of peptide 3a

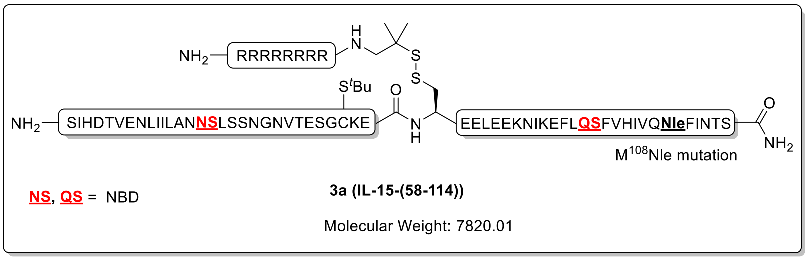

Peptide **3a** was synthesized by general procedure **2.1, 2.2** (Method B) using 200 mg Rink Amide AM resin (loading 0.29 mmol/g) and **Cys-Sc**. Subsequently, after TFA global deprotection (30 mL, TFA/H_2O = 95:5), cold diethyl ether precipitation, HPLC purification (20-70% CH_3CN/H_2O over 30 min) and lyophilization, the desired peptide **3** was obtained as a white powder (156 mg, 20%).

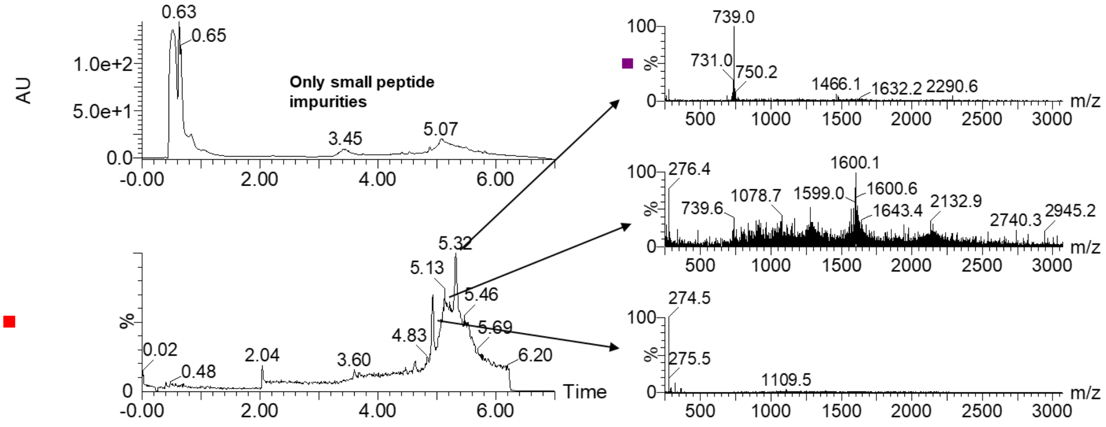

Figure S32. UV (190-400 nm) and MS (300-3000 m/z) trace from UPLC-MS analysis of conventional SPPS of peptide **IL-15-(58-114)**, gradient 20-70% CH_3CN/H_2O containing 0.1% TFA over 7 min at a flow rate of 0.4 mL/min.

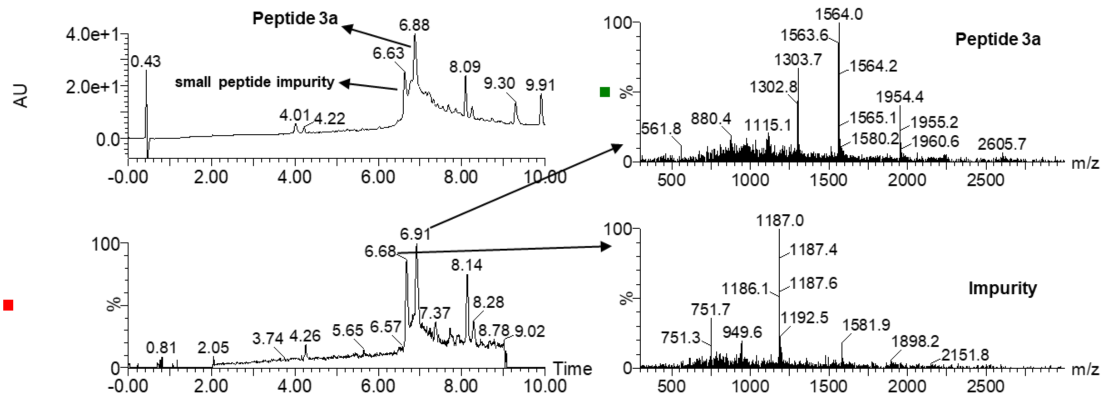

Figure S33. UV (190-400 nm) and MS (300-3000 m/z) trace from UPLC-MS analysis of crude peptide from SPPS enabled by RST-2.0 (via **Cys-Sc**), the major peak at 6.88 min is desired peptide **3a**, gradient 20-70% CH_3CN/H_2O containing 0.1% TFA over 10 min at a flow rate of 0.4 mL/min.

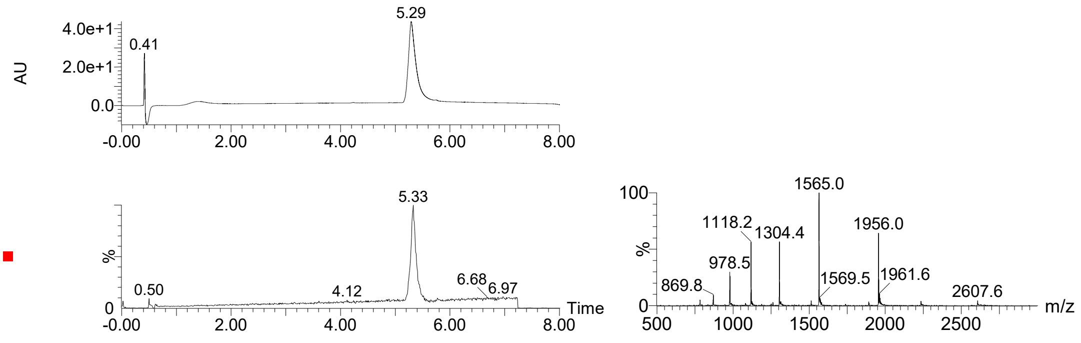

Figure S34. UPLC-MS analysis of purified peptide **3**. Left: UV (190-400 nm) and MS (300-3000 m/z) trace from UPLC-MS analysis of purified **3**, gradient 20-70% CH_3CN/H_2O containing 0.1% TFA over 8 min at a flow rate of 0.4 mL/min; Right: ESI-MS calcd. for $C_{333}H_{557}N_{107}O_{102}S_4$: $[M+3H]^{3+}$ *m/z* = 2607.7, found 2607.6; $[M+4H]^{4+}$ *m/z* = 1956.0, found 1956.0; $[M+5H]^{5+}$ *m/z* = 1565.0, found 1565.0; $[M+6H]^{6+}$ *m/z* = 1304.3, found 1304.4; $[M+7H]^{7+}$ *m/z* = 1118.1, found 1118.2; $[M+8H]^{8+}$ *m/z* = 978.5, found 978.5; $[M+9H]^{9+}$ *m/z* = 869.9, found 869.8.

5.1.3 Synthesis of glycopeptide 3b

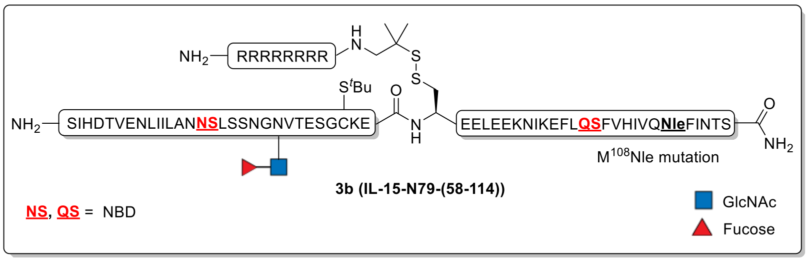

3b was synthesized by general procedure **2.1, 2.2** (**Cys-Sc**, Method B) and **2.6** using 20 mg Rink Amide AM resin (loading 0.29 mmol/g), Fmoc-Asn(Fuc(α1,6)GlcNAc)-OH and **Cys-Sc**. Subsequently, after TFA global deprotection (5 mL, TFA/H_2O = 95:5), cold diethyl ether precipitation, Acetyl removal (8M GnHCl, pH 9.5-10.0, 4 h), HPLC purification (20-70% CH_3CN/H_2O over 30 min) and lyophilization, peptide **3c** was obtained as a white powder (36 mg, 22%).

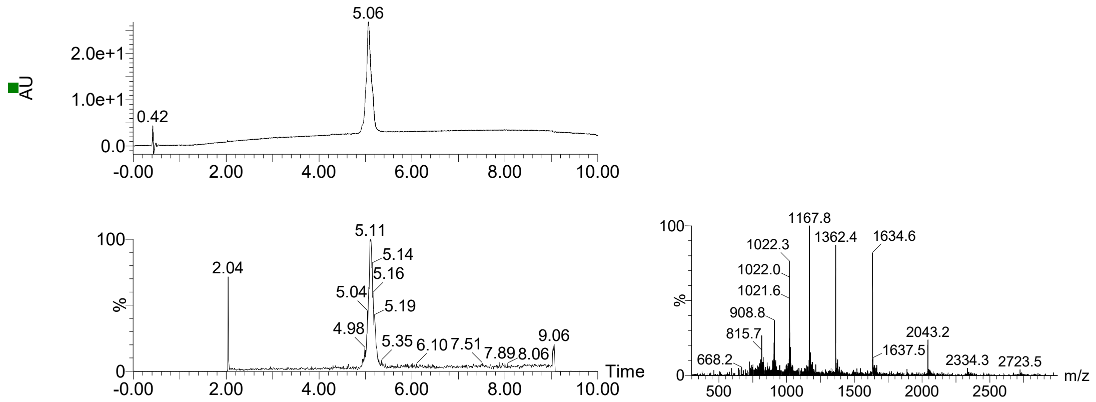

Figure S35. UPLC-MS analysis of purified peptide **3c**. Left: UV (190-400 nm) and MS (300-3000 m/z) trace from UPLC-MS analysis of purified **3c**, gradient 30-80% CH_3CN/H_2O containing 0.1% TFA over 10 min at a flow rate of 0.4 mL/min; Right: ESI-MS calcd. for $C_{347}H_{580}N_{108}O_{111}S_4$: $[M+4H]^{4+}$ *m/z* = 2043.3, found 2043.2; $[M+5H]^{5+}$ *m/z* = 1634.9, found 1634.6; $[M+6H]^{6+}$ *m/z* = 1362.6, found 1362.4; $[M+7H]^{7+}$ *m/z* = 1168.0, found 1167.8; $[M+8H]^{8+}$ *m/z* = 1022.2, found 1022.3; $[M+9H]^{9+}$ *m/z* = 908.7, found 908.8.

5.2 Assembly of peptide segments using STL

5.2.1 Synthesis of peptide 4

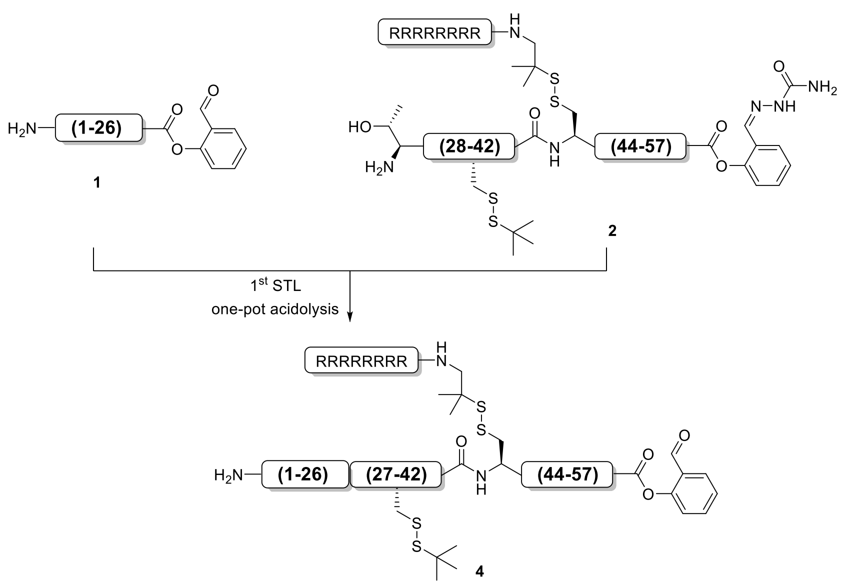

Peptide **1** (**S1**) (21.6 mg, 6.8 μmol, 1.0 equiv) and peptide **2** (40.5 mg, 8.1 μmol, 1.2 equiv) were dissolved in pyridine/HOAc (1/1, v/v) cocktail at a concentration of 10 mM under room temperature. The reaction mixture was stirred at room temperature for 2 h. The solution was poured into cold diethyl ether to precipitate the peptide. After

centrifugation the ether was decanted, and the peptide residue was treated with 5.0 mL of TFA/H_2O/AcAc (95/2.5/2.5) for 2h to generate a native amide bond and to remove the semicarbazone that protects the aldehyde. After completion, 20 mL cold diethyl ether was added to give a white suspension for centrifugation. After centrifugation and decanting diethyl ether, the remaining solid was dissolved by 8.0 mL 30% CH_3CN /H_2O, filtrated by Syringe Filters (PTFE 0.22μm) and subjected to preparative HPLC purification (20-70% CH_3CN/H_2O over 30 min) and lyophilization to give 19.4 mg (36% yield, one pot two steps) of peptide **4** as a white powder.

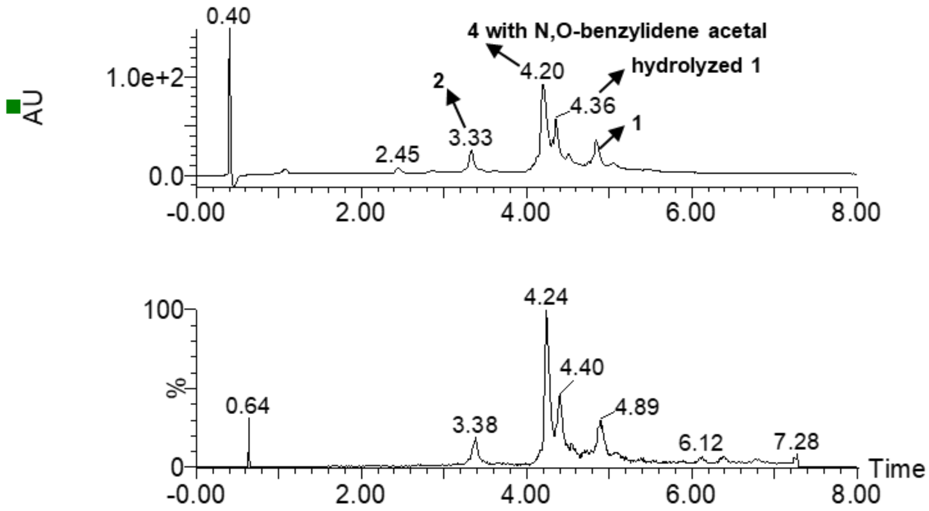

Figure S36. UV (190-400 nm) and MS (300-3000 m/z) trace from UPLC-MS analysis of STL between peptide **1** and **2**, gradient 20-70% CH_3CN/H_2O containing 0.1% TFA over 8 min at a flow rate of 0.4 mL/min.

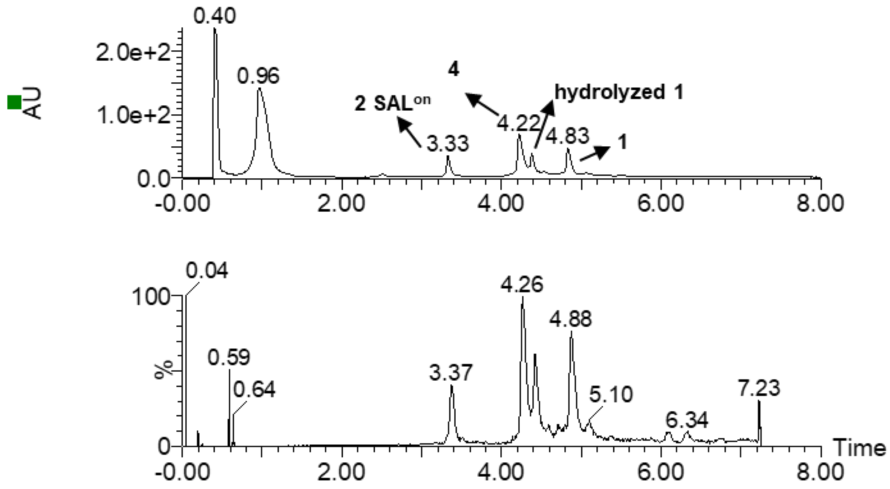

Figure S37. UV (190-400 nm) and MS (300-3000 m/z) trace from UPLC-MS analysis of one-pot acidolysis after STL between peptide **1** and **2**, gradient 20-70% CH_3CN/H_2O containing 0.1% TFA

over 8 min at a flow rate of 0.4 mL/min.

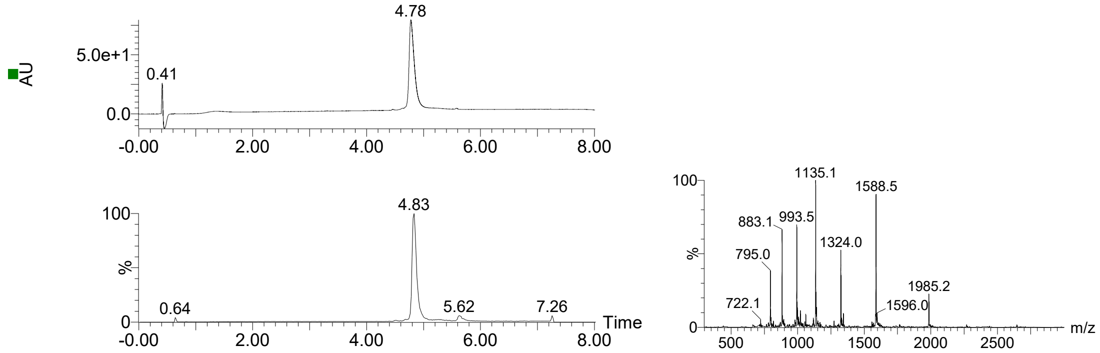

Figure S38. UPLC-MS analysis of purified peptide **4**. Left: UV (190-400 nm) and MS (300-3000 m/z) trace from UPLC-MS analysis of purified **4**, gradient 5-95% CH_3CN/H_2O containing 0.1% TFA over 5 min at a flow rate of 0.4 mL/min; Right: ESI-MS calcd. for $C_{345}H_{571}N_{103}O_{99}S_6$: $[M+4H]^{4+}$ *m/z* = 1985.6, found 1985.2; $[M+5H]^{5+}$ *m/z* = 1588.7, found 1588.5; $[M+6H]^{6+}$ *m/z* = 1324.1, found 1324.0; $[M+7H]^{7+}$ *m/z* = 1135.1, found 1135.1; $[M+8H]^{8+}$ *m/z* = 993.3, found 993.5; $[M+9H]^{9+}$ *m/z* = 883.0, found 883.1; $[M+10H]^{10+}$ *m/z* = 794.8, found 795.0.

5.2.2 Synthesis of peptide 5a

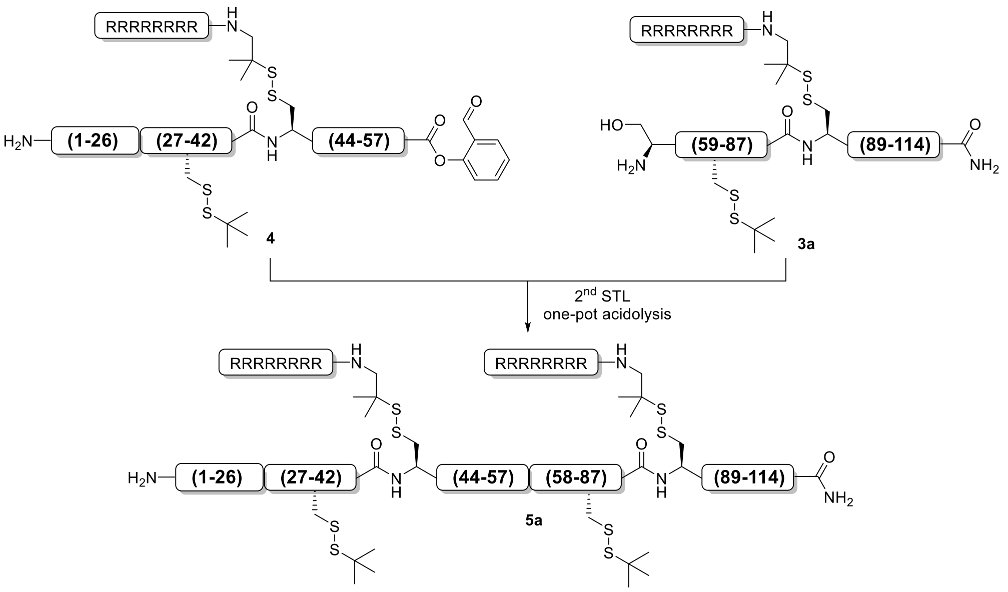

Peptide **4** (2.4 mg, 0.3 μmol, 1 equiv.) and peptide **3a** (2.8 mg, 0.36 μmol, 1.2 equiv.) were dissolved in pyridine/HOAc (1/1, v/v) cocktail at a concentration of 5 mM under room temperature. The reaction mixture was stirred at room temperature for 6 h. The solution was poured into cold diethyl ether to precipitate the peptide. After centrifugation the ether was decanted, and the peptide residue was treated with 0.5 mL

of TFA/H_2O/TIPS (95/2.5/2.5) for 30 min. Then, 5 mL cold diethyl ether was added to give a white suspension for centrifugation. After centrifugation and decanting diethyl ether, the remaining solid was dissolved by 8.0 mL 30% CH_3CN /H_2O, filtrated by Syringe Filters (PTFE 0.22μm) and subjected to preparative HPLC purification (30-80% CH_3CN/H_2O over 30 min) and lyophilization to give 2.4 mg (51% yield, one pot two steps) of peptide **5a** as a white powder.

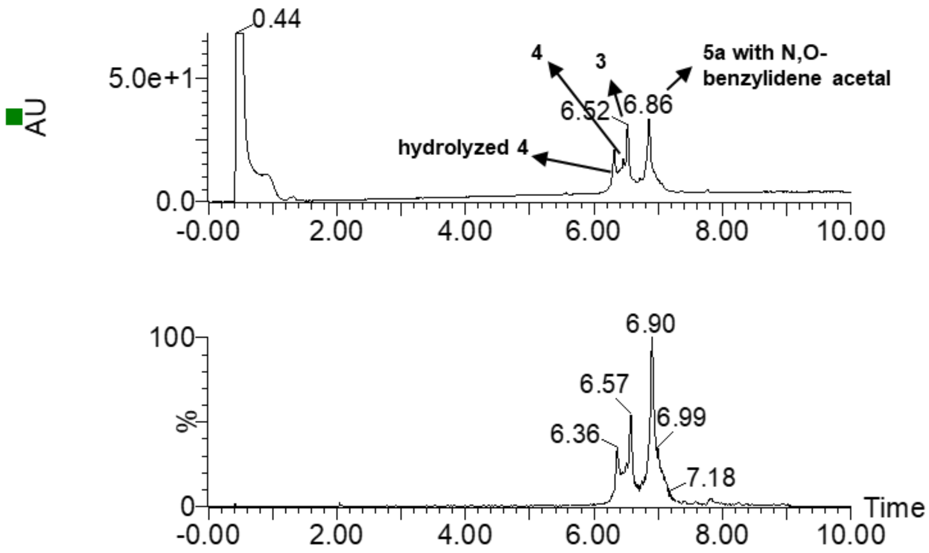

Figure S39. UV (190-400 nm) and MS (300-3000 m/z) trace from UPLC-MS analysis of STL between peptide **4** and **3a**, gradient 20-70% CH_3CN/H_2O containing 0.1% TFA over 8 min at a flow rate of 0.4 mL/min.

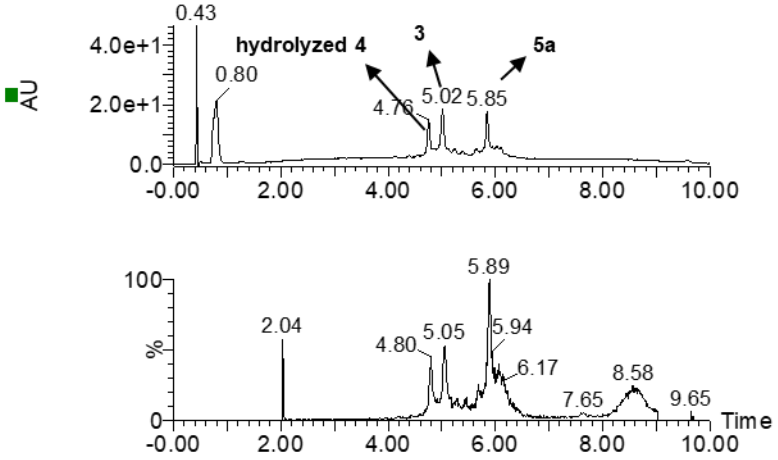

Figure S40. UV (190-400 nm) and MS (300-3000 m/z) trace from UPLC-MS analysis of one-pot acidolysis after STL between peptide **4** and **3a**, gradient 30-80% CH_3CN/H_2O containing 0.1% TFA over 8 min at a flow rate of 0.4 mL/min.

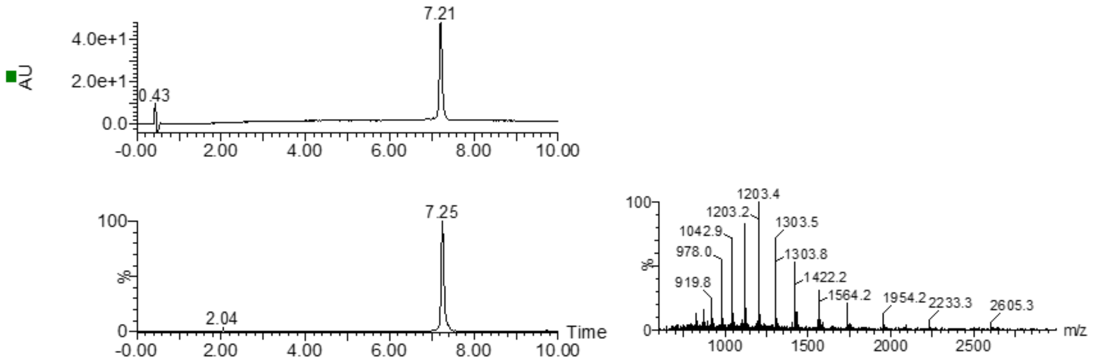

Figure S41. UPLC-MS analysis of purified peptide **5a**. Left: UV (190-400 nm) and MS (300-3000 m/z) trace from UPLC-MS analysis of purified **5a**, gradient 20-70% CH_3CN/H_2O containing 0.1% TFA over 10 min at a flow rate of 0.4 mL/min; Right: ESI-MS calcd. for $C_{671}H_{1122}N_{210}O_{199}S_{10}$: $[M+6H]^{6+}$ *m/z* = 2605.4, found 2605.3; $[M+7H]^{7+}$ *m/z* = 2233.3, found 2233.3; $[M+8H]^{8+}$ *m/z* = 1954.3, found 1954.2; $[M+9H]^{9+}$ *m/z* = 1737.2, found 1737.4; $[M+10H]^{10+}$ *m/z* = 1563.6, found 15634.2; $[M+11H]^{11+}$ *m/z* =1421.6, found 14212.2; $[M+12H]^{12+}$ *m/z* =1303.2, found 1303.5; $[M+13H]^{13+}$ *m/z* =1203.0, found 1203.4; $[M+14H]^{14+}$ *m/z* =1117.2, found 1117.3; $[M+15H]^{15+}$ *m/z* =1042.7, found 1042.9; $[M+11H]^{11+}$ *m/z* =977.6, found 978.0; $[M+11H]^{11+}$ *m/z* =920.2, found 919.8.

5.2.4 Synthesis of glycopeptide 5b

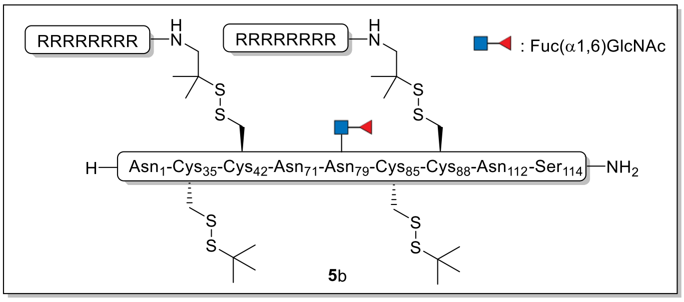

Peptide **4** (7.7, 1 equiv.) and peptide **3b** (5.6 mg, 1.2 equiv.) were dissolved in pyridine/HOAc (1/1, v/v) cocktail at a concentration of 5 mM under room temperature. The reaction mixture was stirred at room temperature for 6 h. The solution was poured into cold diethyl ether to precipitate the peptide. After centrifugation the ether was decanted, and the peptide residue was treated with 0.5 mL of TFA/ACN/H_2O (20/40/40) for 3 h. Then, the solvent was removed by lyophilization and the remaining solid was dissolved by 8.0 mL 30% CH_3CN /H_2O, filtrated by Syringe Filters (PTFE 0.22μm) and subjected to preparative HPLC purification (30-80% CH_3CN/H_2O over 30 min) and lyophilization to give 4.6 mg (42% yield, one pot two steps) of peptide **5b** as a

white powder.

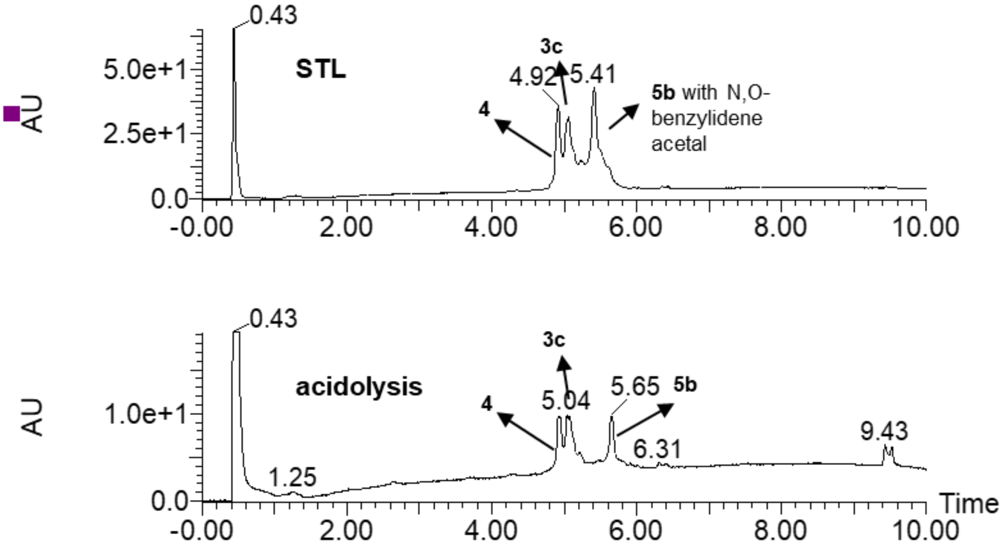

Figure S42. UPLC analysis of STL (up) and one-pot acidolysis (down) between peptide **4** and **3b** gradient 30-80% CH_3CN/H_2O containing 0.1% TFA over 10 min at a flow rate of 0.4 mL/min.

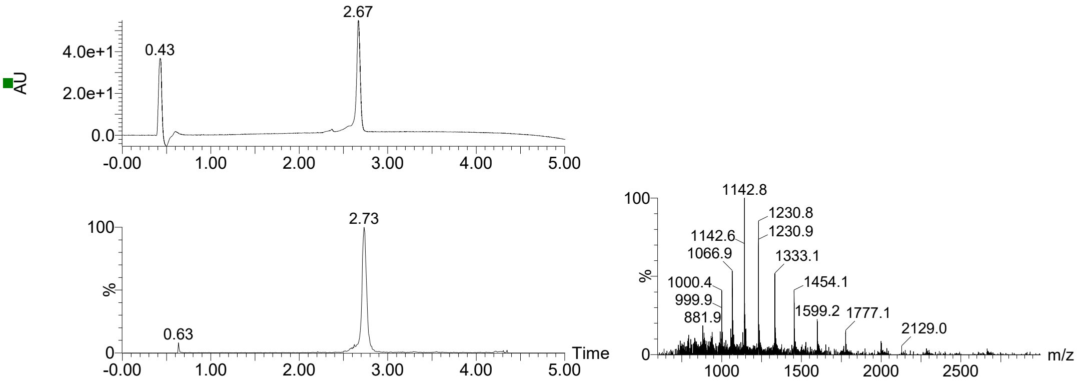

Figure S43. UPLC-MS analysis of purified peptide **5b**. Left: UV (190-400 nm) and MS (600-3000 m/z) trace from UPLC-MS analysis of purified **5b**, gradient 5-95% CH_3CN/H_2O containing 0.1% TFA over 5 min at a flow rate of 0.4 mL/min; Right: ESI-MS calcd. for $C_{685}H_{1145}N_{211}O_{208}S_{10}$: $[M+9H]^{9+}$ *m/z* =1777.2, found 1777.1; $[M+10H]^{10+}$ *m/z* =1599.6, found 1599.2; $[M+11H]^{11+}$ *m/z* =1454.2, found 1454.1; $[M+12H]^{12+}$ *m/z* =1333.1, found 1333.1; $[M+13H]^{13+}$ *m/z* =1230.7, found 1230.8; $[M+14H]^{14+}$ *m/z* =1142.8, found 1142.8; $[M+15H]^{15+}$ *m/z* =1066.7, found 1066.9; $[M+16H]^{16+}$ *m/z* =1000.1, found 1000.4.

5.3 Folding

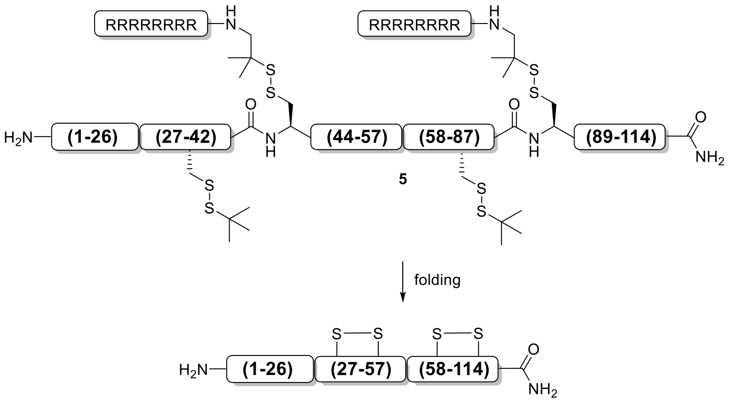

6 (IL-15-WT)

Generally, the folding of synthetic IL-15 was performed with two major steps:

Firstly, linear IL-15 bearing RSTs and –S^{t}Bu protecting group on four cysteines was dissolved in denaturation buffer to break all secondary structure and remove all disulfides. However, when doing this at 50 ºC, it always generated two peaks in UPLC. It was reported that Asn^{77} is a major deamidation site of IL-15. We thus realized that Asn^{77} deamidation (aspartimide formation and subsequent side reaction) might be very fast at 50 ºC. Therefore, this step (denaturation/reduction) was finally performed at 37 ºC with TCEP as reductant.

Secondly, the resulting linear IL-15 solution was diluted to final folding concentration (dilution protocol) or dialyzed against folding buffer containing proper redox pair. It turned out that dilution protocol was not suitable for this folding. Several redox pairs were tested and finally Cysteamine/Cystamine proved to be the best one. However, there was always some [protein+75] side product (cysteamine adduct), so one more dialysis with GSH/GSSG as redox pair was required to obtain separable crude product of folded IL-15.

Folding conditions and results were summarized in **Table S1**.

5.3.1 folding condition screening

Table S1: Folding condition screening for synthetic IL-15

Entry	Redox pair	Folding buffer	Operations	UPLC trace
1	GSH/GSSG 10/1 mM	(1) denaturation: 6M Gn, 0.1 M Tris, 30 mM GSH, pH 8.0; (2) dilution buffer: 0.1 M Tris, 1.5 mM GSSG, pH 8.0.	(1) 50 °C, 2h; (2) 1:2 dilution to 2M Gn, rt, 24h	(1) two peaks in UPLC, likely deamidation; (2) messy, disulfide with GSH, much aggregate
2	GSH/GSSG 10/1 mM	(1) denaturation: 6M Gn, 0.1 M Tris, 30 mM GSH, pH 8.0; (2) dialysis buffer: 2M Gn, 0.1 M Tris, 10/1 mM GSH/GSSG, pH 8.0	(1) 50 °C, 2h; (2) dialysis, 4 °C 24h	Same as above
3	TCEP 2 mM; GSH/GSSG 10/1 mM	(1) denaturation: 6M Gn, 0.1 M Tris, 2 mM TCEP, pH 8.0; (2) dialysis buffer: 2M Gn, 0.1 M Tris, 10/1 mM GSH/GSSG, pH 8.0	(1) 37 °C, 2h; (2) dialysis, 4 °C 24h	Same as above
4	TCEP, 2 mM; 2-BME/ /2,2' dithiodiethanol 10/1 mM	(1) denaturation: 6M Gn, 0.1 M Tris, 2 mM TCEP, pH 8.0; (2) dialysis buffer: 1M Gn, 0.1 M Tris, 10/1 mM 2-BME/ /2,2' dithiodiethanol, pH 8.0	(1) 37 °C, 2h; (2) dialysis, 4 °C 24h	All protein precipitate
5	TCEP, 2 mM; Cysteamine/ Cystamine, 10/1 mM	(1) denaturation: 6M Gn, 0.1 M Tris, 2 mM TCEP, pH 8.0; (2) dialysis buffer: 1M Gn, 0.1 M Tris, 10/1 mM Cysteamine/Cystamine, pH 8.0	(1) 37 °C, 2h; (2) dialysis, 4 °C 24h	Sharp peak in UPLC, but overlap with [M+75] (disulfide with cysteamine)
6	TCEP, 2 mM; Cysteamine/ Cystamine, 10/1 mM; GSH/GSSG 10/1 mM	(1) denaturation: 6M Gn, 0.1 M Tris, 2 mM TCEP, pH 8.0; (2) dialysis buffer: 1M Gn, 0.1 M Tris, 10/1 mM Cysteamine/Cystamine, pH 8.0 (3) dialysis buffer: 1M Gn, 0.05 M Tris, 1/0.5 mM GSH/GSSG, pH 8.0	(1) 37 °C, 2h; (2) dialysis, 4 °C 24h (3) dialysis, 4 °C 12h	Sharp peak in UPLC, separable folded protein

Note: experiments were performed 1–3 times.

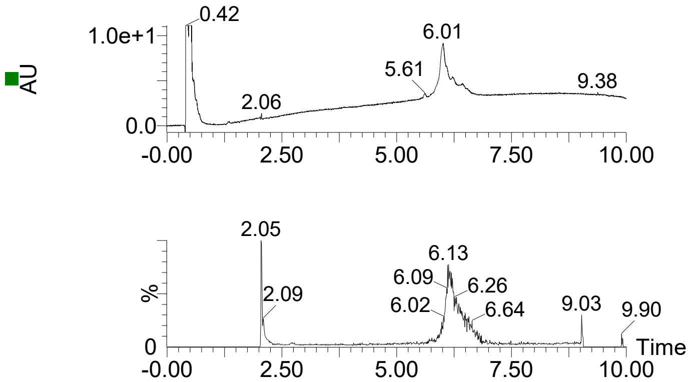

Figure S44. Folding of **5a** (IL-15 with RSTs) by performing tag cleavage and protein folding concurrently, yet ESI-MS indicates that the major product is IL-15-RST adduct, with +1353 (Arg_8 tag) +88 (–S^{t}Bu) higher molecular weight than linear IL-15.

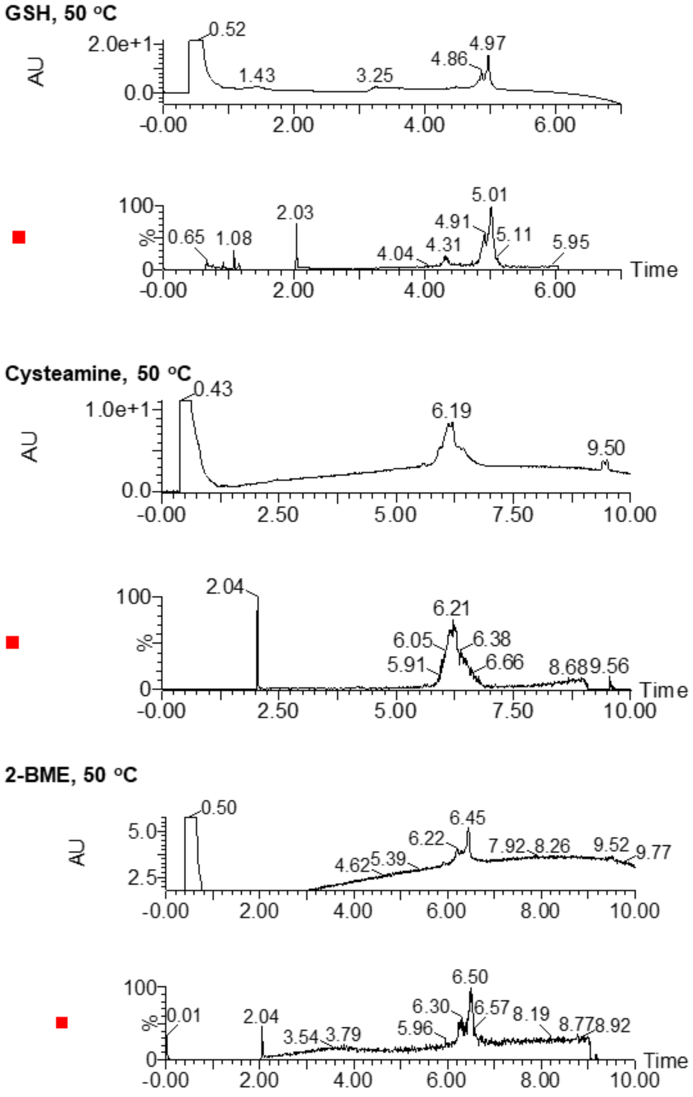

Figure S45. UPLC analysis of synthetic IL-15 denaturation/reduction using GSH, Cysteamine or 2-BME as reductant at 50 °C.

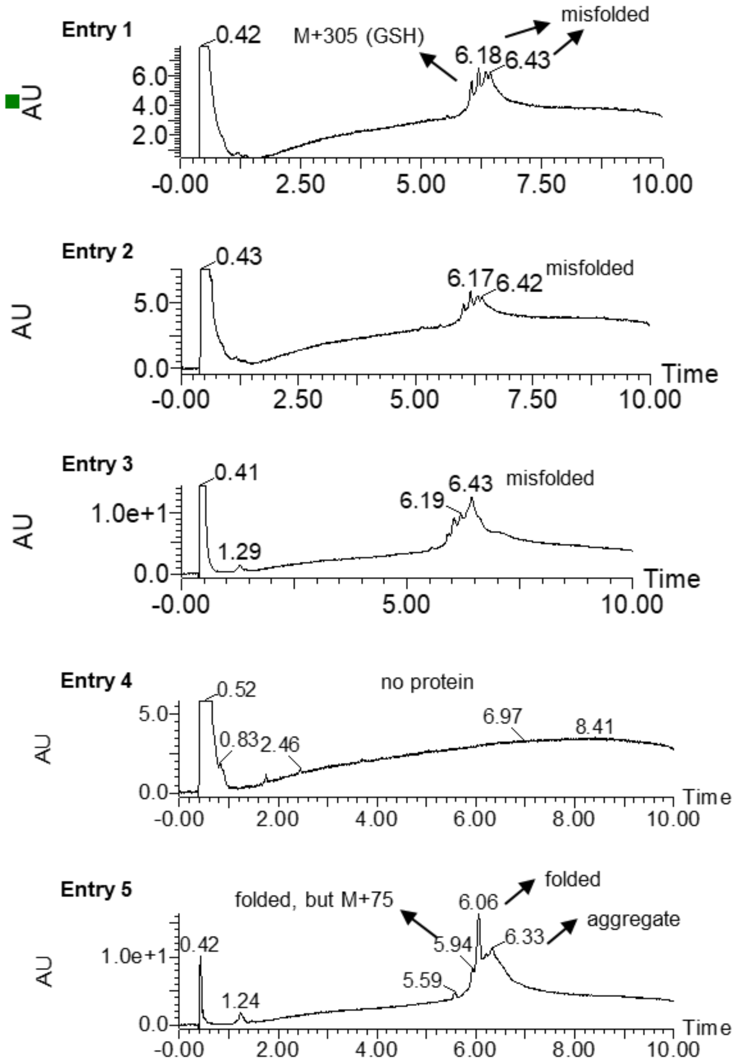

Figure S46. UPLC analysis of folding of synthetic IL-15 using different redox pairs and folding operation.

5.3.2 final folding protocol

Peptide **5a** (2 mg) was dissolved in 10 mL denaturation buffer (6M GnHCl, 0.1M Tris, 2 mM TCEP, pH 8.0) and incubated at 37 ºC for 2h. The linear IL-15 solution was transferred to a dialysis tube (MW cut-off 3.5 KDa) and dialysis against the first folding buffer (1M GnHCl, 0.1M Tris, 10/2.5 mM Cysteamine/Cystamine, pH 8.5) at 4 ºC for 24h. Then, the dialysis tube was transferred to the second folding buffer (1M GnHCl, 0.05M Tris, 1/0.5 mM GSH/GSSG, pH 8.0) at 4 ºC for 12h. The protein solution was concentrated by ultrafiltration using Millipore tube (MWCO 3000) until the volume was around 2 mL. After that, the concentrated protein was transferred to a centrifuge

tube and underwent a centrifugation (20000 rpm, 10 min). The crude protein solution was checked by UPLC-MS and then subjected to preparative HPLC purification using C4 column (30-80% ACN/H_2O, 35 min) and lyophilization to give folded **IL-15-WT** (**6a**) (0.27 mg, 11%).

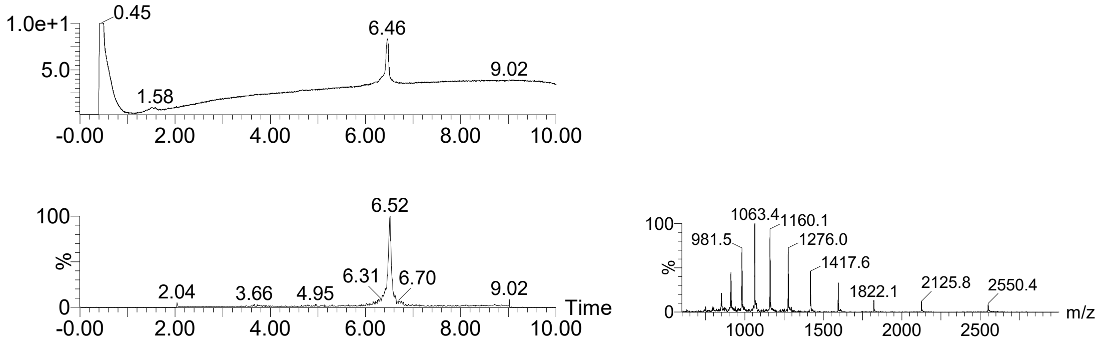

Figure S47. UPLC-MS analysis of denaturation/reduction of peptide **5**.

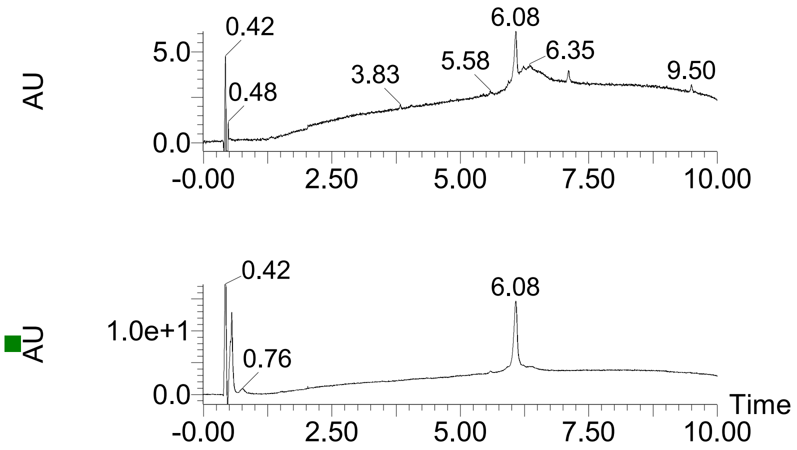

Figure S48. UPLC trace of crude folding solution (up) and purified folded **IL-15-WT** (down).

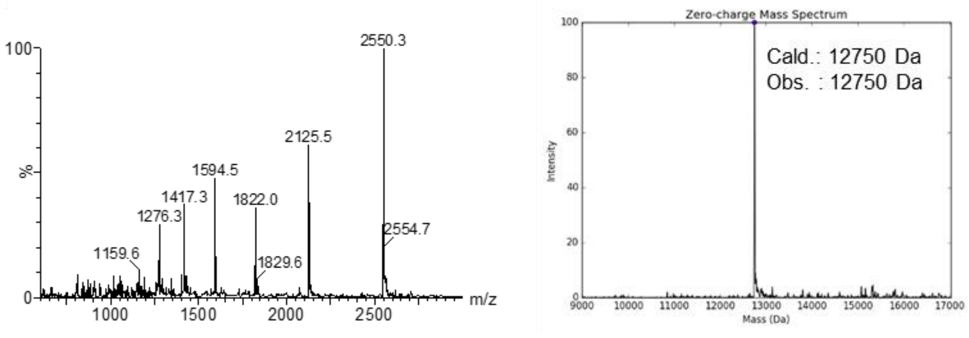

Figure S49. ESI-MS (left) and deconvolution MS (right) of folded **IL-15-WT**.

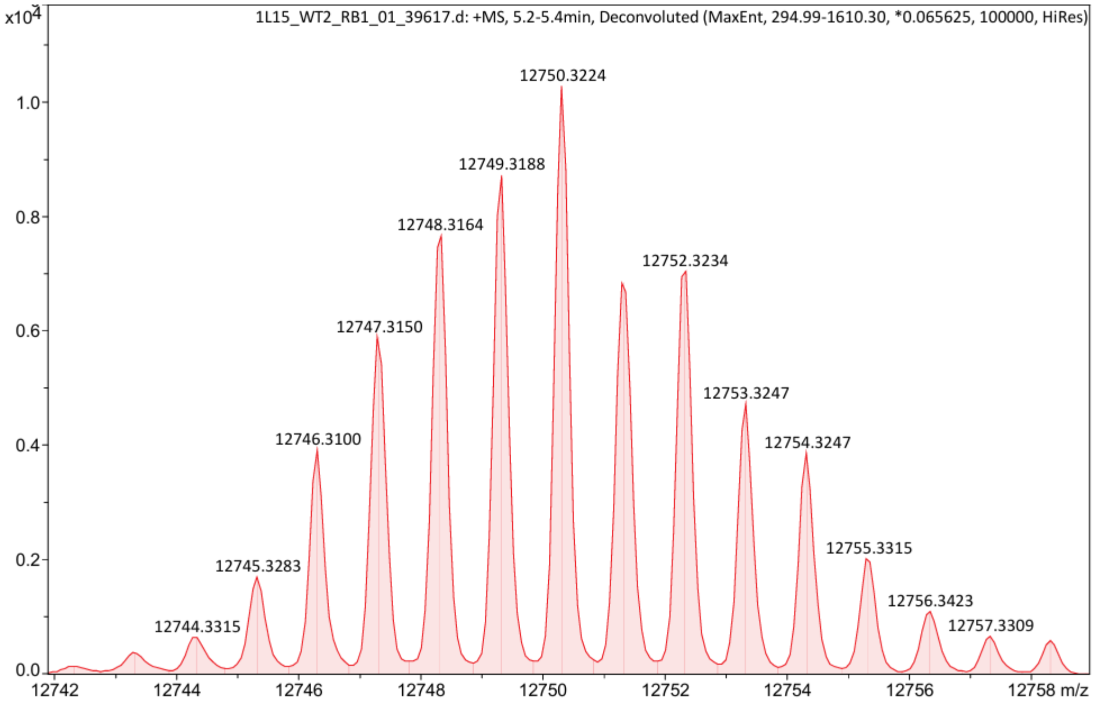

Figure S50. ESI-HRMS of folded **IL-15-WT**. Cald. for $C_{559}H_{892}N_{144}O_{183}S_6$ m/z = 12750.4700, measured 12750.3224. HRMS indicated the formation of two disulfide bonds.

5.3.3 folding of 6b (IL-15-N79)

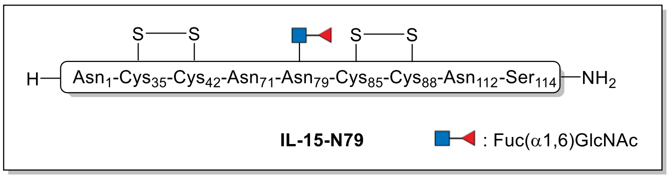

IL-15-N79 was folded using the same folding protocol (**5.3.2**) as **IL-15-WT**. After HPLC purification, 0.9 mg **5b** provided 95 ug desired protein (13% yield). The amount of protein was determined by reconstituting the protein powder in PBS and measuring the UV absorption at 280 nm (NanoDrop UV spectrometer, thermo scientific).

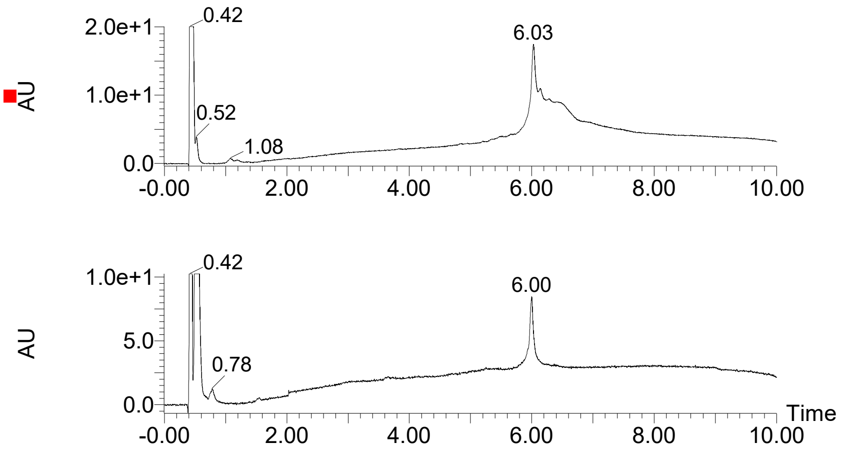

Figure S51. UPLC analysis of crude (up) and purified (down) folded **IL-15-N79** (**6b**), gradient 30-80% CH_3CN/H_2O containing 0.1% TFA over 10 min at a flow rate of 0.4 mL/min.

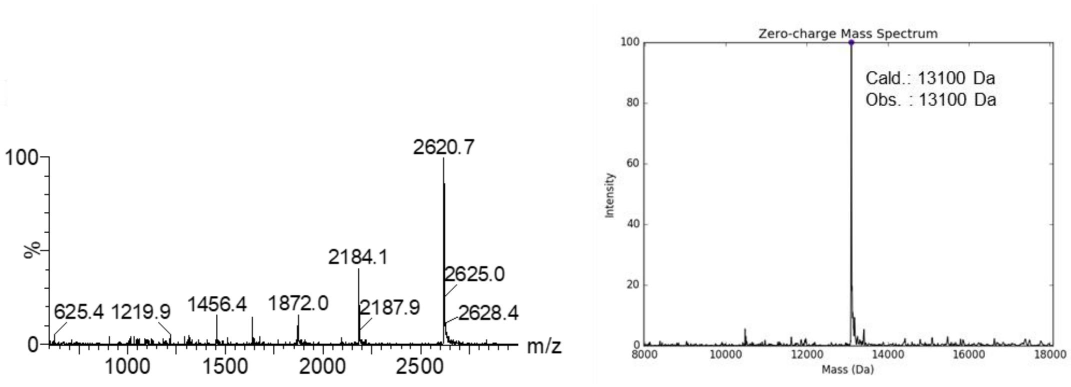

Figure S52. ESI-MS (left) and deconvolution MS (right) of folded **IL-15-N79** (**6b**).

5.4 SDS-PAGE

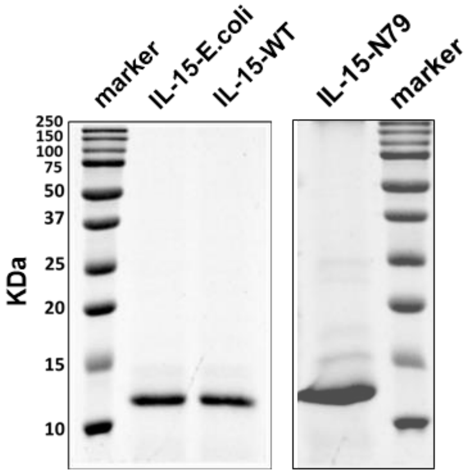

Figure S53. SDS-PAGE of folded **IL-15-WT** (**6a**) and **IL-15-N79** (**6b**) using reducing condition.

5.5 Circular dichroism

The synthesized **IL-15-WT**, **IL-15-N79** and E. coli expressed human IL-15 (MedChemExpress, HY-P7034) were dissolved by PBS. The final concentration of the synthetic and recombinant IL-15 proteins was adjusted to around 0.25 mg/mL. (The protein concentration was determined by Thermo Scientific NanoDrop UV-Vis spectrophotometer). The CD spectrum was measured by a J-815 circular dichroism spectrometer (JASCO), each sample was scanned for 1 time at room temperature using PBS buffer (pH 7.4) as blank in a 0.1 cm cell. The combined spectra were drawn with Origin 9.

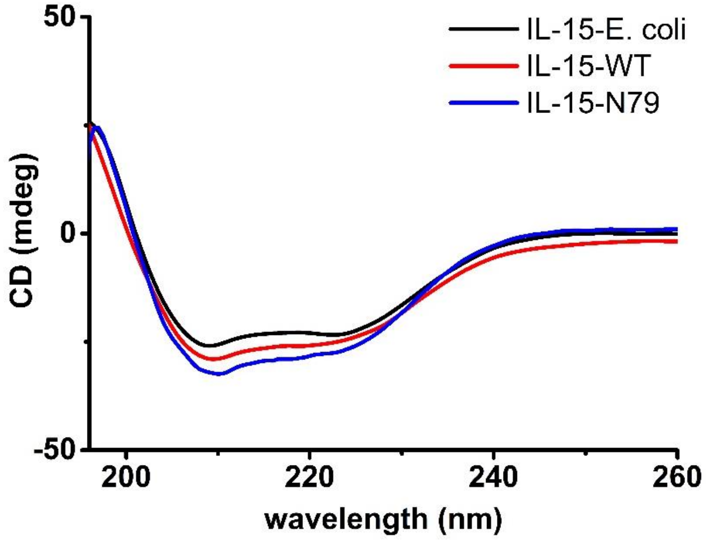

Figure S54. CD of synthetic and recombinant IL-15 proteins.

6. Synthesis of IL-2-WT and IL-2-AzK

6.1 Synthesis of peptide segments

6.1.1 Synthesis of peptide 7

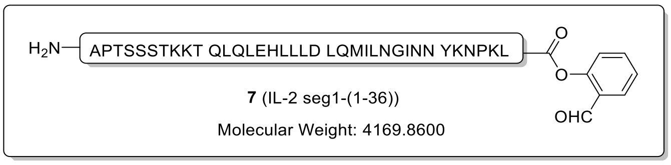

Peptide **7** was synthesized by general procedure **2.1** using 500 mg 2-chlorotrityl chloride resin (loading 0.5 mmol/g). Subsequently, following procedure **2.3**, **2.5**, TFA global deprotection (30 mL, TFA/H_2O/Pyruvic acid = 95:2.5:2.5), cold diethyl ether precipitation, HPLC purification (20-70% CH_3CN/H_2O over 30 min) and lyophilization, the desired peptide SAL ester was obtained as a white powder (325 mg, 31%).[4]

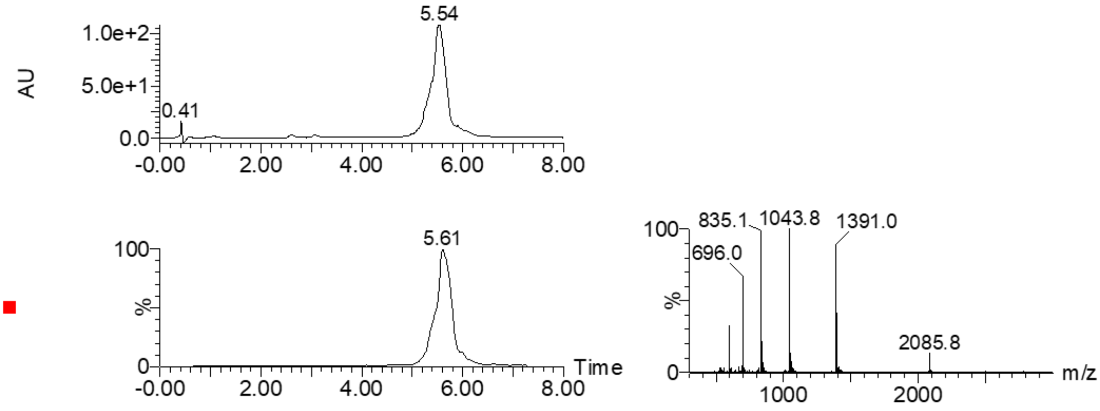

Figure S55. UPLC-MS analysis of purified peptide **7**. Left: UV (190-400 nm) and MS (300-3000 m/z) trace from UPLC-MS analysis of purified **7**, gradient 20-70% CH_3CN/H_2O containing 0.1% TFA over 8 min at a flow rate of 0.4 mL/min; Right: ESI-MS calcd. for $C_{187}H_{307}N_{49}O_{56}S$: $[M+2H]^{2+}$ m/z = 2085.9, found 2085.8; $[M+3H]^{3+}$ m/z = 1391.0, found 1391.0; $[M+4H]^{4+}$ m/z = 1043.5, found 1043.8; $[M+5H]^{5+}$ m/z = 835.0, found 835.1; $[M+6H]^{6+}$ m/z = 696.0, found 696.0.

6.1.2 Synthesis of peptide 8a

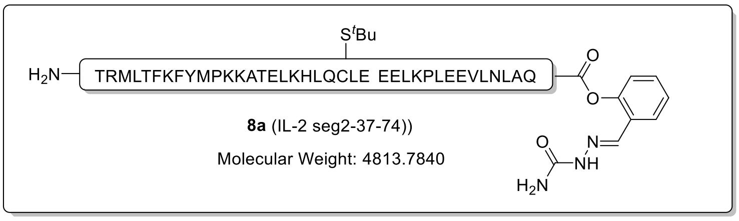

Peptide **8a** was synthesized by general procedure **2.1** using 500 mg 2-chlorotrityl chloride resin (loading 0.5 mmol/g). Subsequently, following procedure **2.3**, **2.5**, TFA global deprotection (30 mL, TFA/H_2O/Pyruvic acid = 95:2.5:2.5), cold diethyl ether precipitation, HPLC purification (20-70% CH_3CN/H_2O over 30 min) and lyophilization, the desired peptide SAL ester was obtained as a white powder (265 mg, 22%).[4]

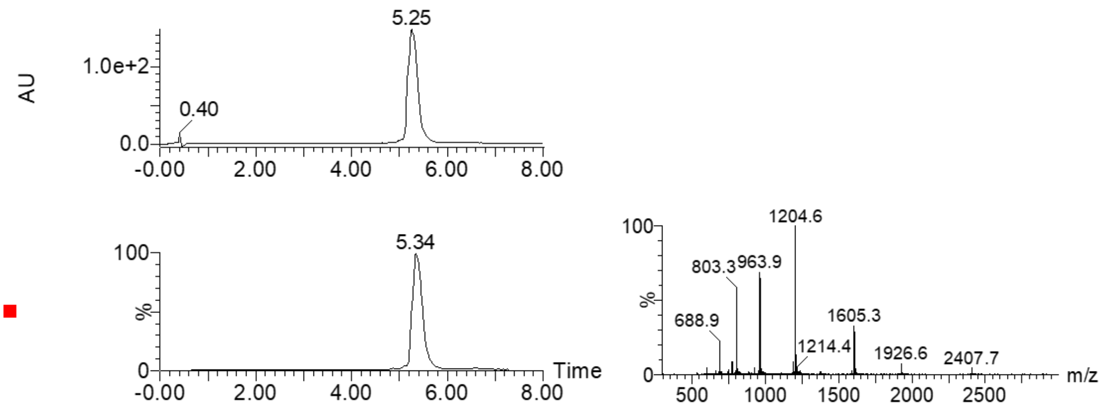

Figure S56. UPLC-MS analysis of purified peptide **8a**. Left: UV (190-400 nm) and MS (300-3000 m/z) trace from UPLC-MS analysis of purified **8a**, gradient 20-70% CH_3CN/H_2O containing 0.1% TFA over 8 min at a flow rate of 0.4 mL/min; Right: ESI-MS calcd. for $C_{219}H_{352}N_{54}O_{59}S_4$: $[M+2H]^{2+}$ *m/z* = 2407.9, found 2407.7; $[M+3H]^{3+}$ *m/z* = 1605.6, found 1605.3; $[M+4H]^{4+}$ *m/z* = 1204.4, found 1204.6; $[M+5H]^{5+}$ *m/z* = 963.8, found 963.9; $[M+6H]^{6+}$ *m/z* = 803.3, found 803.3; $[M+7H]^{7+}$ *m/z* = 688.7, found 688.9.

6.1.3 Synthesis of peptide 8b

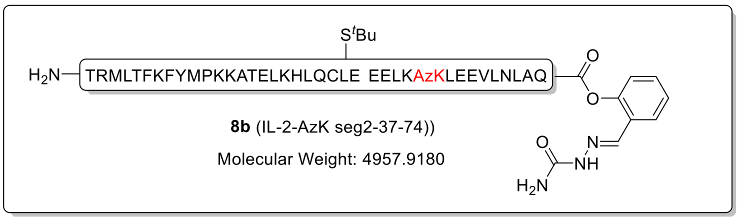

Peptide **8b** was synthesized by general procedure **2.1** using 200 mg 2-chlorotrityl chloride resin (loading 0.5 mmol/g). Subsequently, following procedure **2.3**, **2.5**, TFA global deprotection (30 mL, TFA/H_2O/Pyruvic acid = 95:2.5:2.5), cold diethyl ether precipitation, HPLC purification (20-70% CH_3CN/H_2O over 30 min) and lyophilization, the desired peptide SAL ester was obtained as a white powder (40 mg, 8%).

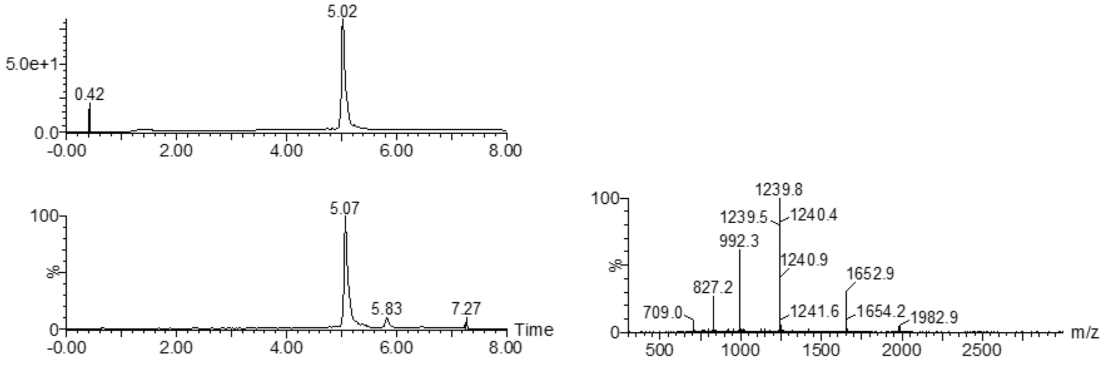

Figure S57. UPLC-MS analysis of purified peptide **8b**. Left: UV (190-400 nm) and MS (300-3000 m/z) trace from UPLC-MS analysis of purified **8b**, gradient 20-70% CH_3CN/H_2O containing 0.1% TFA over 8 min at a flow rate of 0.4 mL/min; Right: ESI-MS calcd. for $C_{223}H_{360}N_{58}O_{61}S_4$: $[M+3H]^{3+}$ m/z = 1653.6, found 1652.9; $[M+4H]^{4+}$ m/z = 1240.5, found 1239.8; $[M+5H]^{5+}$ m/z = 992.6, found 992.3; $[M+6H]^{6+}$ m/z = 827.3, found 827.2; $[M+7H]^{7+}$ m/z = 709.3, found 709.0.

6.1.4 Synthesis of peptide 9

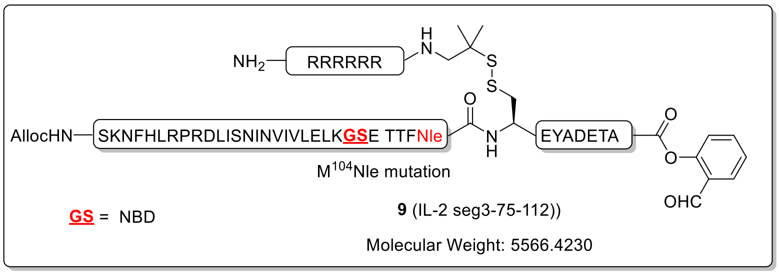

Peptide **9** was synthesized by general procedure **2.1** and **2.2** (**Cys-Sc**, method B) using 500 mg 2-chlorotrityl chloride resin (loading 0.5 mmol/g). Subsequently, following procedure **2.3**, **2.5**, TFA global deprotection (30 mL, TFA/H_2O/Pyruvic acid = 95:2.5:2.5), cold diethyl ether precipitation, HPLC purification (20-70% CH_3CN/H_2O over 30 min) and lyophilization, the desired peptide SAL ester was obtained as a white powder (210 mg, 15%).

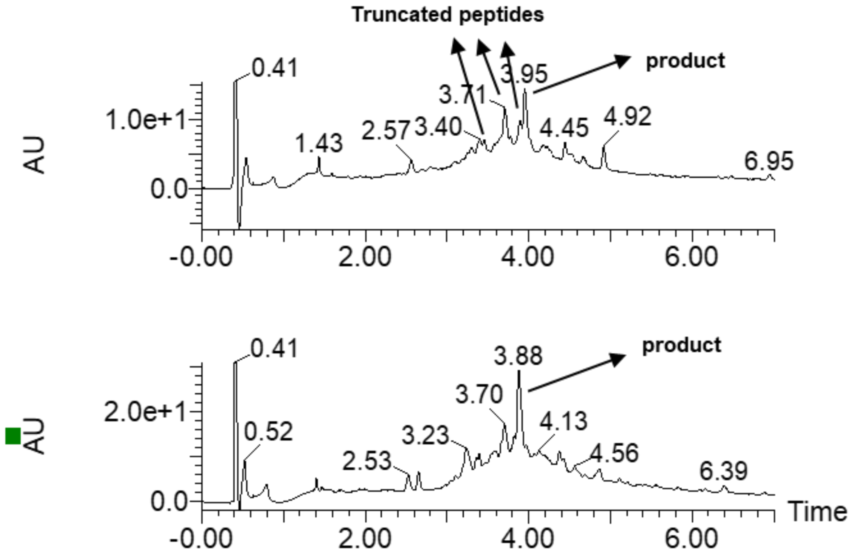

Figure S58. UPLC-MS analysis of crude peptide **9** from SPPS using **Cys-N** (up) and **Cys-Sc** (down), gradient 20-70% CH_3CN/H_2O containing 0.1% TFA over 8 min at a flow rate of 0.4 mL/min.

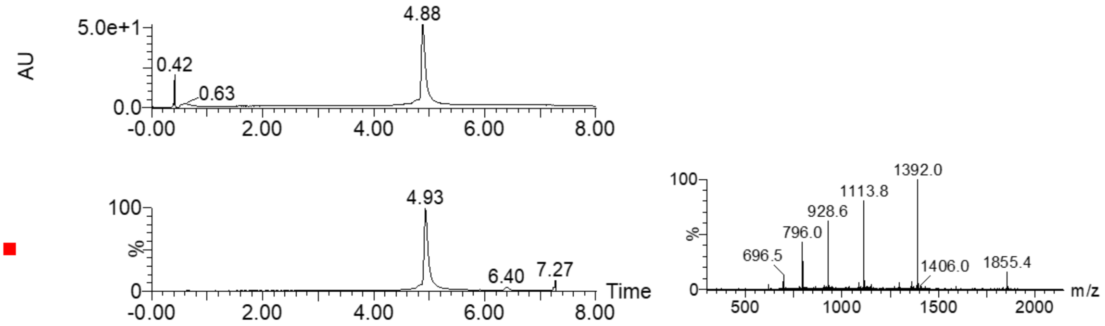

Figure S59. UPLC-MS analysis of purified peptide **9**. Left: UV (190-400 nm) and MS (300-3000 m/z) trace from UPLC-MS analysis of purified **9**, gradient 20-70% CH_3CN/H_2O containing 0.1% TFA over 8 min at a flow rate of 0.4 mL/min; Right: ESI-MS calcd. for $C_{243}H_{396}N_{76}O_{70}S_2$: $[M+3H]^{3+}$ m/z = 1856.5, found 1855.4; $[M+4H]^{4+}$ m/z = 1392.6, found 1392.0; $[M+5H]^{5+}$ m/z = 1114.3, found 1113.8; $[M+6H]^{6+}$ m/z = 928.7, found 928.6; $[M+7H]^{7+}$ m/z = 796.2, found 796.0; $[M+8H]^{8+}$ m/z = 696.8, found 696.5.

6.1.4 Synthesis of peptide 10

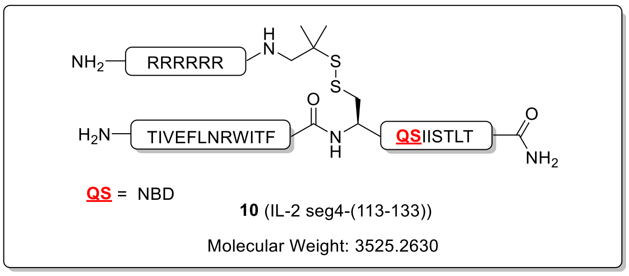

Peptide **9** was synthesized by general procedure **2.1** and **2.2** (**Cys-N**, method A) using 500 mg Rink amide AM resin (loading 0.29 mmol/g). Subsequently, after TFA global deprotection (30 mL, TFA/H_2O = 95:5), cold diethyl ether precipitation, HPLC purification (20-70% CH_3CN/H_2O over 30 min) and lyophilization, the desired peptide was obtained as a white powder (211 mg, 24%).

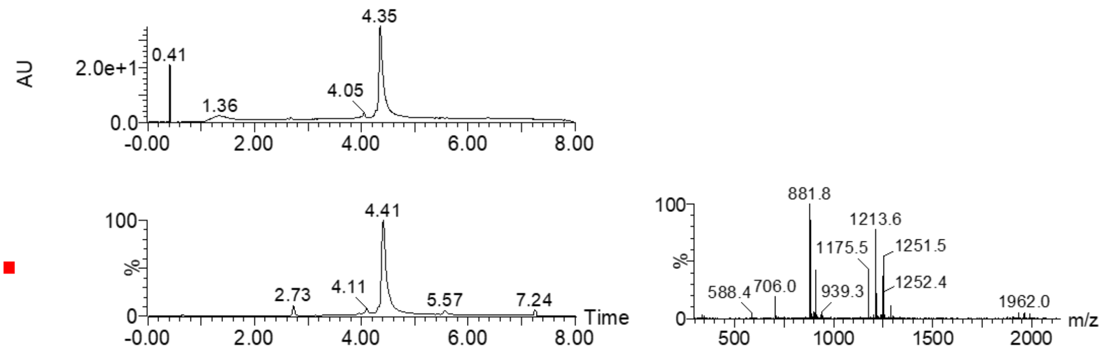

Figure S60. UPLC-MS analysis of purified peptide **10**. Left: UV (190-400 nm) and MS (300-3000 m/z) trace from UPLC-MS analysis of purified **10**, gradient 20-70% CH_3CN/H_2O containing 0.1% TFA over 8 min at a flow rate of 0.4 mL/min; Right: ESI-MS calcd. for $C_{155}H_{263}N_{53}O_{37}S_2$: $[M+3H]^{3+}$ *m/z* = 1176.1, found 1175.5; $[M+3H+TFA]^{3+}$ *m/z* = 1214.1, found 1213.8; $[M+3H+2TFA]^{3+}$ *m/z* = 1252.1, found 1252.4; $[M+4H]^{4+}$ *m/z* = 882.3, found 881.8; $[M+5H]^{5+}$ *m/z* =706.1, found 706.0; $[M+6H]^{6+}$ *m/z* = 588.5, found 588.4.

6.2 Peptide assembly by STL

6.2.1 Synthesis of peptide 11a by first STL/acidolysis

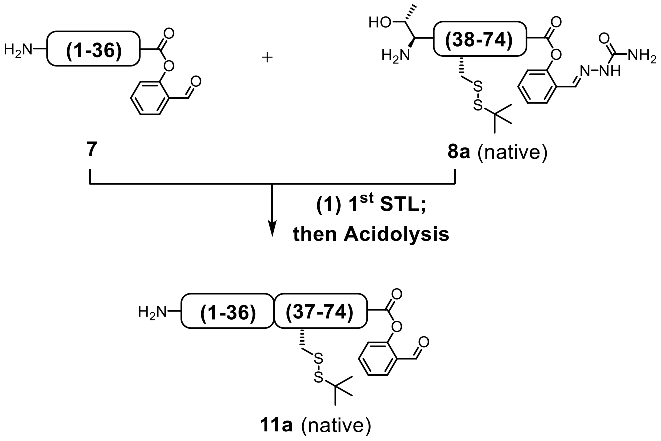

Peptide **7** (20.0 mg, 4.8 μmol, 1.0 equiv) and peptide **8a** (27.9 mg, 5.8 μmol, 1.2 equiv) were dissolved in pyridine/HOAc (1/1, v/v) cocktail at a concentration of 10 mM under

room temperature. The reaction mixture was stirred at room temperature for 2 h. The solution was poured into cold diethyl ether to precipitate the peptide. After centrifugation the ether was decanted, and the peptide residue was treated with 5.0 mL of TFA/H_2O/Pyruvic acid (95/2.5/2.5) for 2h. After completion, 20 mL cold diethyl ether was added to give a white suspension for centrifugation. After centrifugation and decanting diethyl ether, the remaining solid was dissolved by 8.0 mL 30% CH_3CN /H_2O, filtrated by Syringe Filters (PTFE 0.22μm) and subjected to preparative HPLC purification (20-70% CH_3CN/H_2O over 30 min) and lyophilization to give 16.6 mg (39% yield, one pot two steps) of peptide **11a** as a white powder.[4]

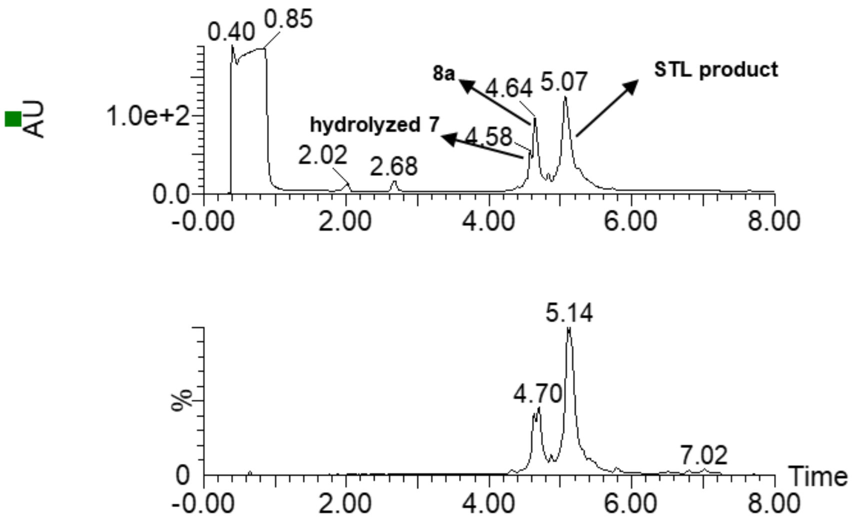

Figure S61. UV (190-400 nm) and MS (300-3000 m/z) trace from UPLC-MS analysis of STL between peptide **7** and **8a**, gradient 20-70% CH_3CN/H_2O containing 0.1% TFA over 8 min at a flow rate of 0.4 mL/min.

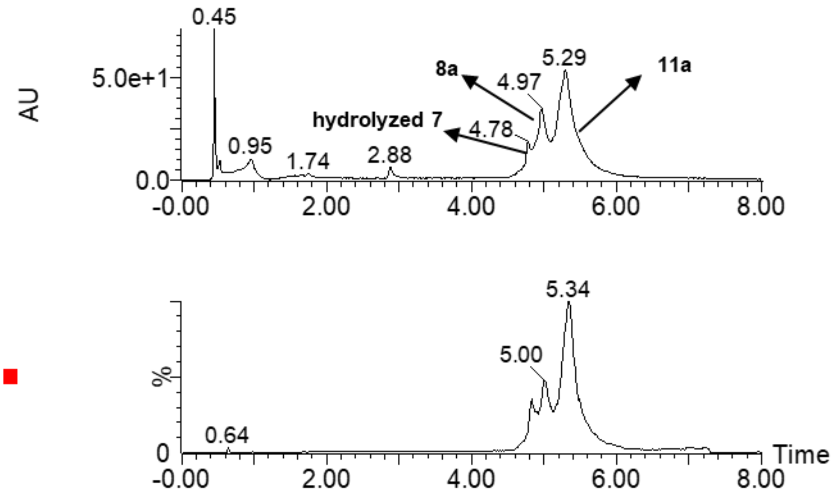

Figure S62. UV (190-400 nm) and MS (300-3000 m/z) trace from UPLC-MS analysis of one-pot acidolysis after STL between peptide **7** and **8a**, gradient 20-70% CH_3CN/H_2O containing 0.1% TFA over 8 min at a flow rate of 0.4 mL/min.

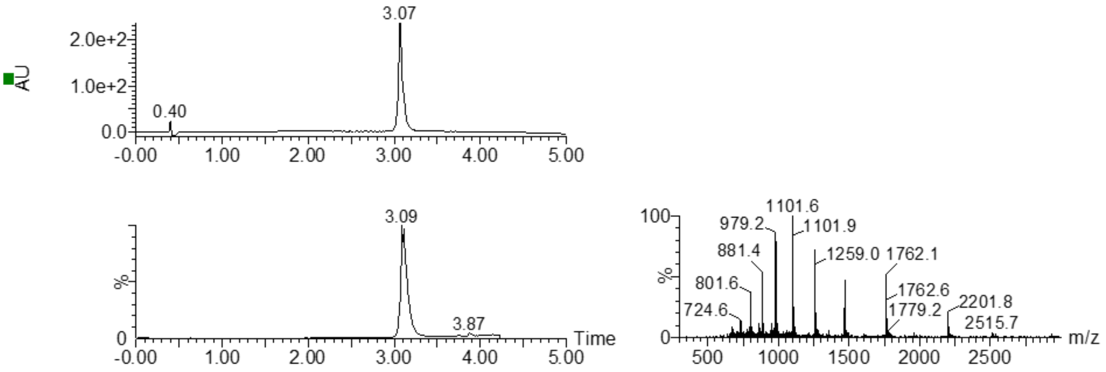

Figure S63. UPLC-MS analysis of purified peptide **11a**. Left: UV (190-400 nm) and MS (300-3000 m/z) trace from UPLC-MS analysis of purified **11a**, gradient 5-95% CH_3CN/H_2O containing 0.1% TFA over 5 min at a flow rate of 0.4 mL/min; Right: ESI-MS calcd. for $C_{398}H_{650}N_{100}O_{113}S_5$ (Molecular Weight: 8804.4650): $[M+4H]^{4+}$ *m/z* = 2202.1, found 2201.8; $[M+5H]^{5+}$ *m/z* = 1761.9, found 1762.1; $[M+6H]^{6+}$ *m/z* = 1468.4, found 1468.4; $[M+7H]^{7+}$ *m/z* = 1258.8, found 1259.0; $[M+8H]^{8+}$ *m/z* = 1101.6, found 1101.6; $[M+9H]^{9+}$ *m/z* = 979.3, found 979.2; $[M+10H]^{10+}$ *m/z* = 881.4, found 881.4; $[M+11H]^{11+}$ *m/z* = 801.4, found 801.6.

6.2.2 Synthesis of peptide 11b by first STL/acidolysis

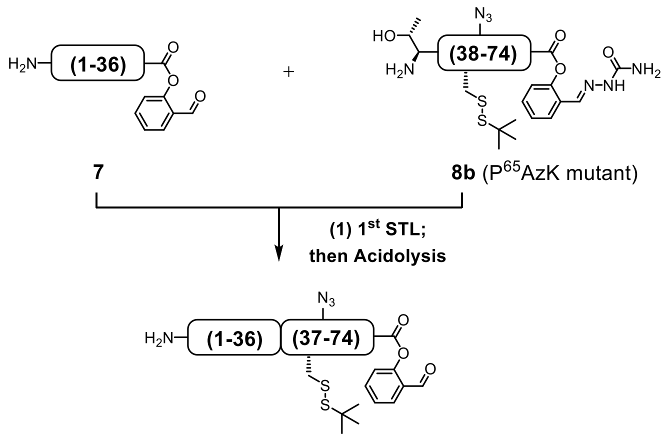

Peptide **7** (20.0 mg, 4.8 μmol, 1.0 equiv) and peptide **8b** (26.0 mg, 5.3 μmol, 1.1 equiv) were dissolved in pyridine/HOAc (1/1, v/v) cocktail at a concentration of 10 mM under room temperature. The reaction mixture was stirred at room temperature for 2 h. The solution was poured into cold diethyl ether to precipitate the peptide. After

centrifugation the ether was decanted, and the peptide residue was treated with 5.0 mL of TFA/H_2O/Pyruvic acid (95/2.5/2.5) for 2h. After completion, 20 mL cold diethyl ether was added. After centrifugation and decanting ether, the peptide was dissolved by 8.0 mL 30% CH_3CN /H_2O, filtrated by Syringe Filters (PTFE 0.22μm) and subjected to preparative HPLC purification (20-70% CH_3CN/H_2O over 30 min) and lyophilization to give 15.0 mg (35% yield, one-pot) of peptide **11b** as a white powder.

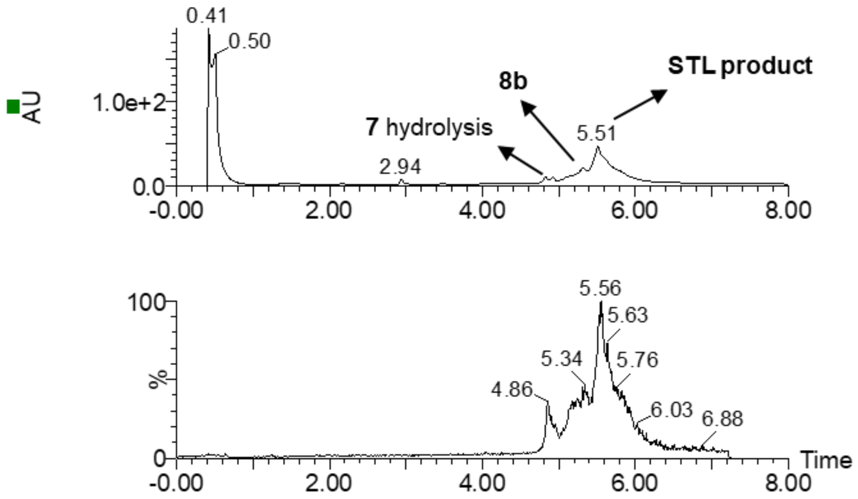

Figure S64. UV (190-400 nm) and MS (300-3000 m/z) trace from UPLC-MS analysis of STL between peptide **7** and **8b**, gradient 20-70% CH_3CN/H_2O containing 0.1% TFA over 8 min at a flow rate of 0.4 mL/min.

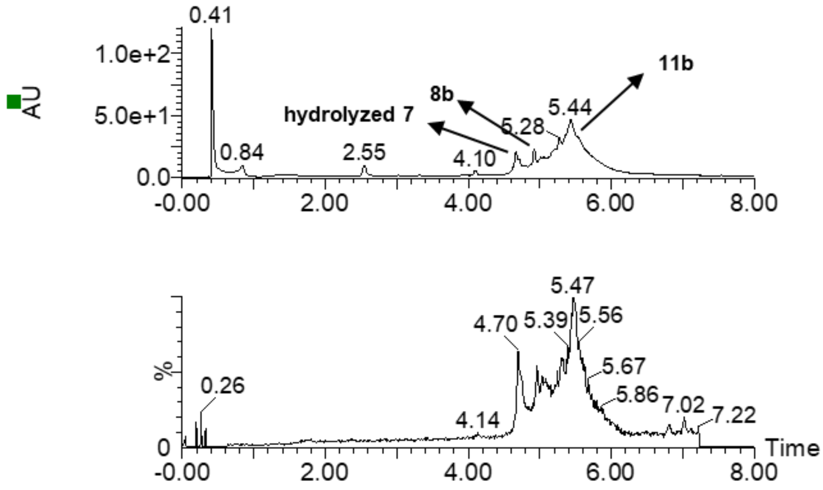

Figure S65. UV (190-400 nm) and MS (300-3000 m/z) trace from UPLC-MS analysis of one-pot acidolysis after STL between peptide **7** and **8b**, gradient 20-70% CH_3CN/H_2O containing 0.1% TFA over 8 min at a flow rate of 0.4 mL/min.

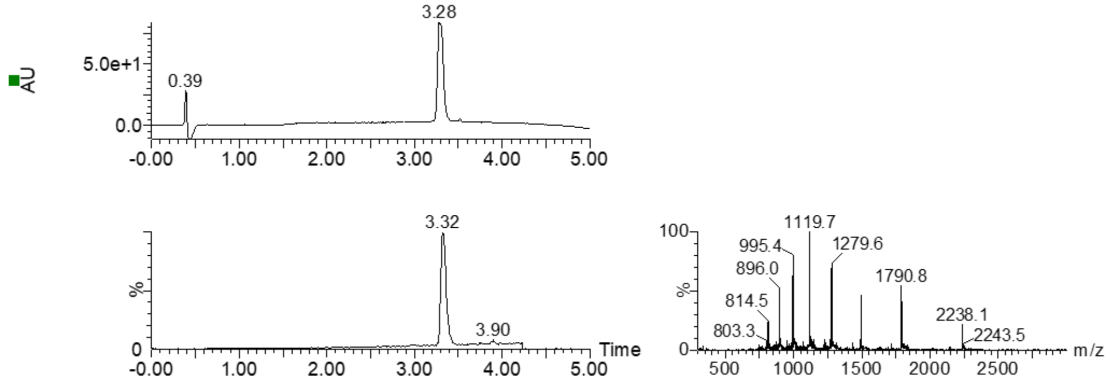

Figure S66. UPLC-MS analysis of purified peptide **11b**. Left: UV (190-400 nm) and MS (300-3000 m/z) trace from UPLC-MS analysis of purified **11b**, gradient 5-95% CH_3CN/H_2O containing 0.1% TFA over 5 min at a flow rate of 0.4 mL/min; Right: ESI-MS calcd. for $C_{402}H_{658}N_{104}O_{115}S_5$ (Molecular Weight: 8948.5990): $[M+4H]^{4+}$ *m/z* = 2238.1, found 2238.1; $[M+5H]^{5+}$ *m/z* = 1790.7, found 1790.8; $[M+6H]^{6+}$ *m/z* = 1492.4, found 1492.5; $[M+7H]^{7+}$ *m/z* = 1279.4, found 1279.6; $[M+8H]^{8+}$ *m/z* = 1119.6, found 1119.7; $[M+9H]^{9+}$ *m/z* = 995.3, found 995.4; $[M+10H]^{10+}$ *m/z* = 895.9, found 896.0; $[M+11H]^{11+}$ *m/z* = 814.5, found 814.5.

6.2.3 Synthesis of peptide 12 by second STL/deAlloc

Peptide **9** (20.6 mg, 3.6 μmol, 1.0 equiv) and peptide **10** (29.0 mg, 5.4 μmol, 1.5 equiv) were dissolved in Collidine/HOAc/DMSO (1/1/0.2, v/v/v) cocktail at a concentration of 5 mM under room temperature. The reaction mixture was stirred at room temperature for 16 h. The solution was poured into cold diethyl ether to precipitate the peptide. After centrifugation the ether was decanted, and the peptide residue was dissolved by 2 mL HOAc containing 20 mg $Pd(PPh_3)_4$ and 80 mg 1,3-dimethyl barbituric acid and stirred vigorously for 1h. Reaction was monitored by UPLC-MS to prevent much side reaction. After completion, 20 mL cold diethyl ether was added. After centrifugation and decanting ether, the peptide was dissolved by 8.0 mL 30% CH_3CN /H_2O, filtrated by Syringe Filters (PTFE 0.22μm) and subjected to preparative C4-HPLC purification (20-70% CH_3CN/H_2O over 30 min) and lyophilization to give 7.2 mg (22% yield, one-pot) of peptide **12** as a white powder.

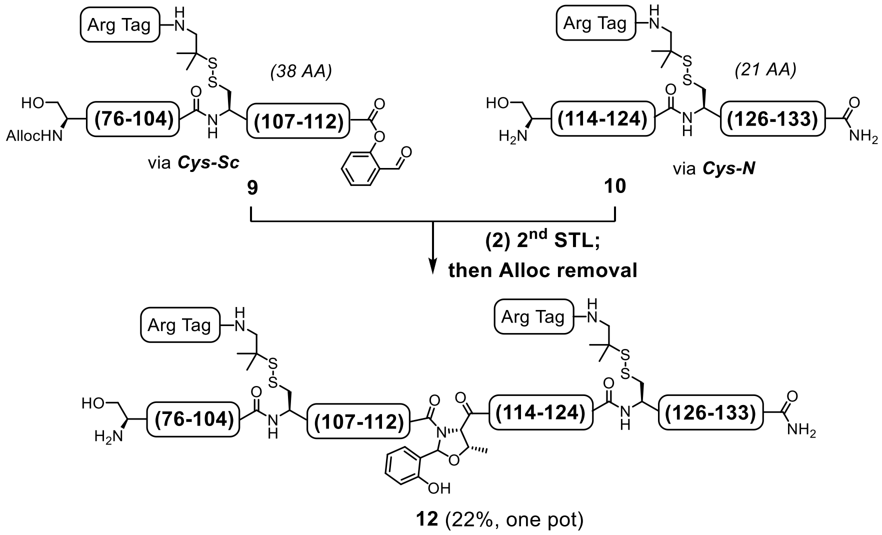

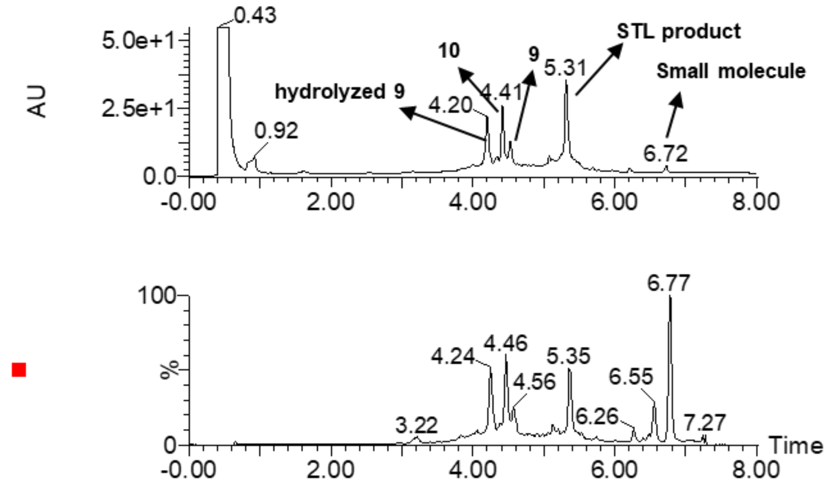

Figure S67. UV (190-400 nm) and MS (300-3000 m/z) trace from UPLC-MS analysis of STL between peptide **9** and **10**, gradient 20-70% CH_3CN/H_2O containing 0.1% TFA over 8 min at a flow rate of 0.4 mL/min.

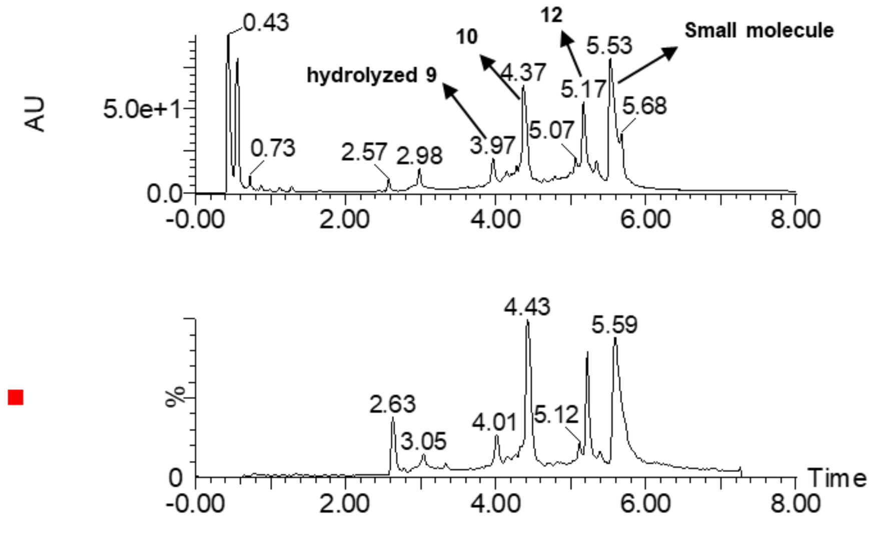

Figure S68. UV (190-400 nm) and MS (300-3000 m/z) trace from UPLC-MS analysis of one-pot Alloc removal after STL between peptide **9** and **10**, gradient 20-70% CH_3CN/H_2O containing 0.1% TFA over 8 min at a flow rate of 0.4 mL/min.

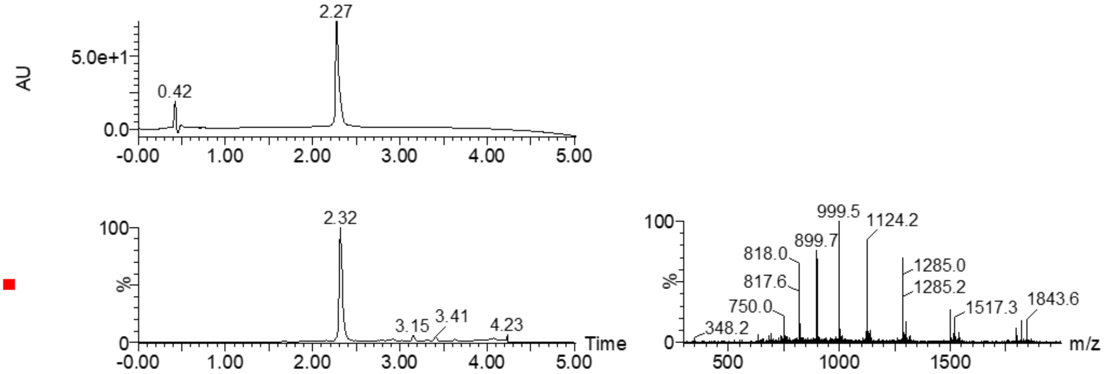

Figure S69. UPLC-MS analysis of purified peptide **12**. Left: UV (190-400 nm) and MS (300-3000 m/z) trace from UPLC-MS analysis of purified **12**, gradient 25-95% CH_3CN/H_2O containing 0.1% TFA over 5 min at a flow rate of 0.4 mL/min; Right: ESI-MS calcd. for $C_{394}H_{653}N_{129}O_{104}S_4$ (Molecular Weight: 8989.5970): $[M+5H+2TFA]^{5+}$ *m/z* = 1844.5, found 1843.6; $[M+6H+TFA]^{6+}$ *m/z* = 1518.3, found 1517.3; $[M+7H]^{7+}$ *m/z* = 1285.2, found 1285.2; $[M+8H]^{8+}$ *m/z* = 1124.7, found 1124.2; $[M+9H]^{9+}$ *m/z* = 999.8, found 999.5; $[M+10H]^{10+}$ *m/z* = 899.9, found 899.7; $[M+11H]^{11+}$ *m/z* = 818.2, found 818.0.

6.2.4 Synthesis of peptide (protein) 13a by third STL/acidolysis

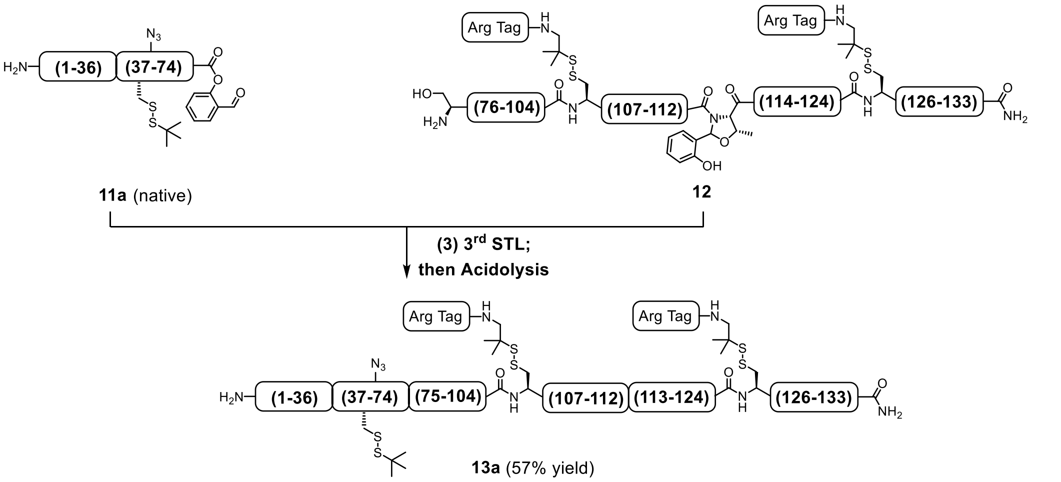

Peptide **11a** (4.1 mg, 1.3 equiv) and peptide **12** (3.2 mg, 1.0 equiv) were dissolved in Collidine/HOAc/DMSO (1/1/0.2, v/v/v) cocktail at a concentration of 3 mM under room temperature. The reaction mixture was stirred at room temperature for 12 h. The solution was poured into cold diethyl ether to precipitate the peptide. After centrifugation the ether was decanted, and the peptide residue was treated with 0.5 mL

of TFA/H_2O/TIPS (95/2.5/2.5) for 30min. After completion, 3.0 mL cold diethyl ether was added. After centrifugation and decanting ether, the peptide was dissolved by 5.0 mL 30% CH_3CN /H_2O, filtrated by Syringe Filters (PTFE 0.22μm) and subjected to preparative HPLC purification (30-80% CH_3CN/H_2O over 30 min) and lyophilization to give 3.6 mg (57% yield, one-pot) of peptide **13a** as a white powder.

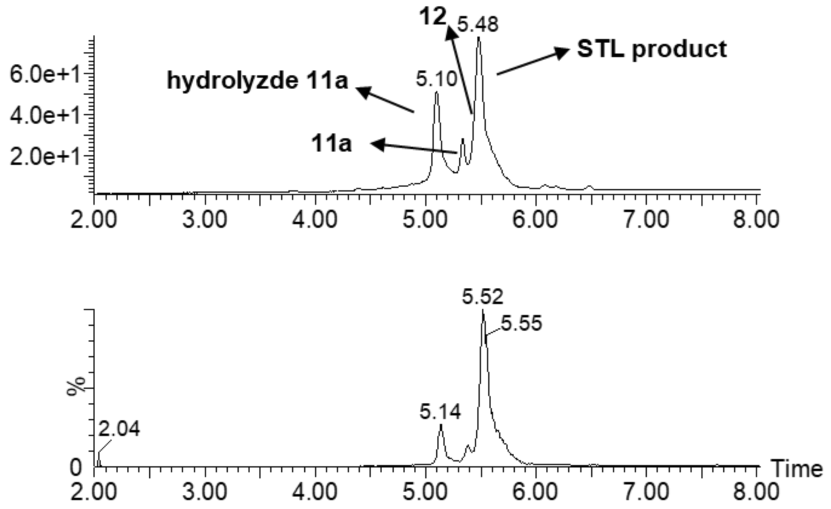

Figure S70. UV (190-400 nm) and MS (300-3000 m/z) trace from UPLC-MS analysis of STL between peptide **11a** and **12**, gradient 30-80% CH_3CN/H_2O containing 0.1% TFA over 10 min at a flow rate of 0.4 mL/min.

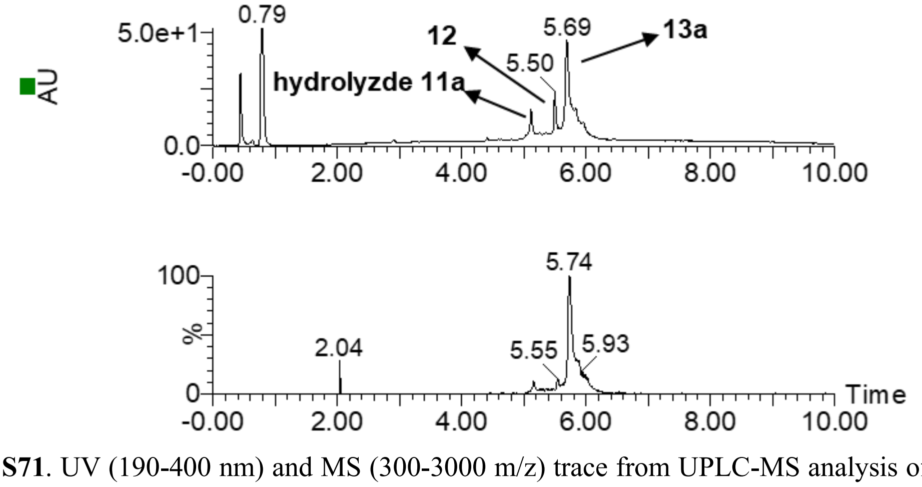

Figure S71. UV (190-400 nm) and MS (300-3000 m/z) trace from UPLC-MS analysis of one-pot acidolysis after STL between peptide **11a** and **12**, gradient 30-80% CH_3CN/H_2O containing 0.1% TFA over 10 min at a flow rate of 0.4 mL/min.

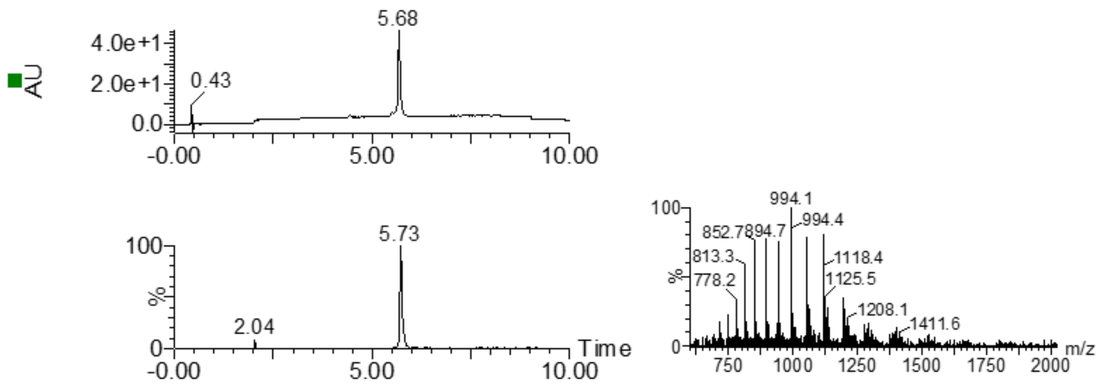

Figure S72. UPLC-MS analysis of purified peptide **13a** (with R_8 and R_6 tag). Left: UV (190-400 nm) and MS (300-3000 m/z) trace from UPLC-MS analysis of purified **13a**, gradient 30-80% CH_3CN/H_2O containing 0.1% TFA over 10 min at a flow rate of 0.4 mL/min; Right: ESI-MS calcd. for $C_{790}H_{1317}N_{237}O_{216}S_9$ (Molecular Weight: 17880.2090): $[M+16H]^{16+}$ *m/z* = 1118.5, found 1118.4; $[M+17H]^{17+}$ *m/z* = 1052.8, found 1052.7; $[M+18H]^{18+}$ *m/z* = 994.3, found 994.1; $[M+19H]^{19+}$ *m/z* = 942.1, found 942.3; $[M+20H]^{20+}$ *m/z* = 895.0, found 894.7; $[M+21H]^{21+}$ *m/z* = 852.4, found 852.7; $[M+22H]^{22+}$ *m/z* = 813.7, found 813.3; $[M+23H]^{23+}$ *m/z* = 778.4, found 778.2.

6.2.5 Synthesis of peptide (protein) 13b by third STL/acidolysis

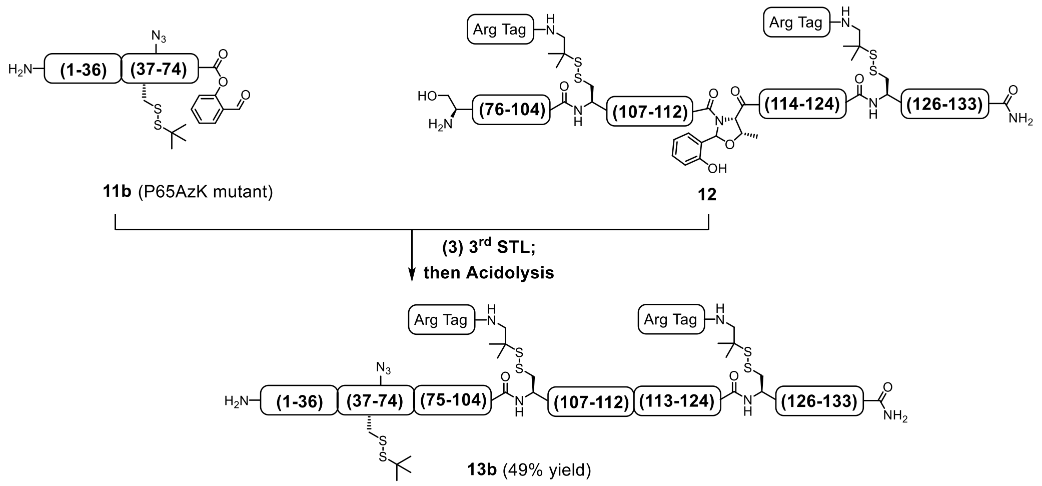

Peptide **11b** (1.8 mg, 1.2 equiv) and peptide **12** (1.7 mg, 1.0 equiv) were dissolved in Collidine/HOAc/DMSO (1/1/0.2, v/v/v) cocktail at a concentration of 3 mM under room temperature. The reaction mixture was stirred at room temperature for 12 h. The solution was poured into cold diethyl ether to precipitate the peptide. After centrifugation the ether was decanted, and the peptide residue was treated with 0.5 mL of TFA/H_2O (95/5) for 30min. After completion, 3.0 mL cold diethyl ether was added. After centrifugation and decanting ether, the peptide was dissolved by 5.0 mL 30% CH_3CN /H_2O, filtrated by Syringe Filters (PTFE 0.22μm) and subjected to preparative

HPLC purification (30-80% CH_3CN/H_2O over 30 min) and lyophilization to give 1.6 mg (49% yield, one-pot) of peptide **13b** as a white powder.

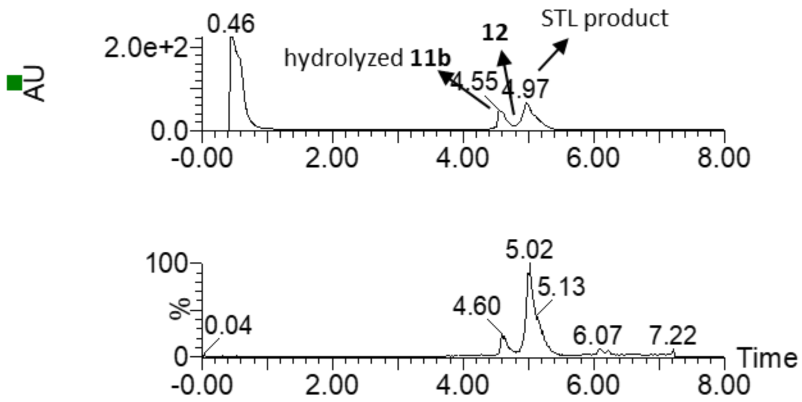

Figure S73. UV (190-400 nm) and MS (300-3000 m/z) trace from UPLC-MS analysis of STL between peptide **11b** and **12**, gradient 30-80% CH_3CN/H_2O containing 0.1% TFA over 8 min at a flow rate of 0.4 mL/min.

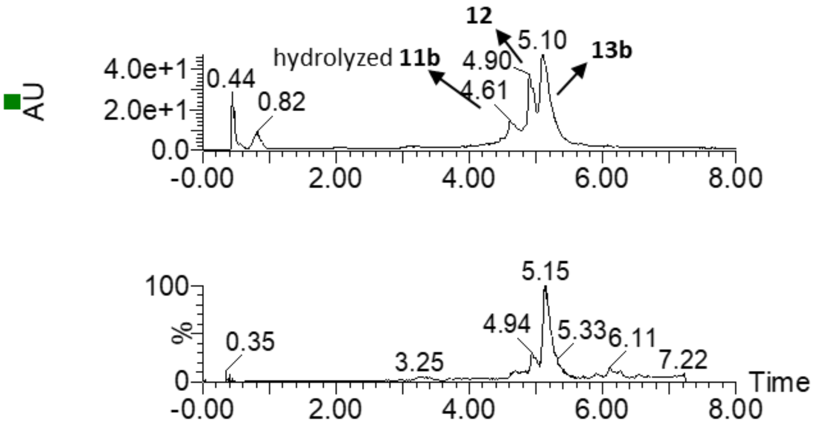

Figure S74. UV (190-400 nm) and MS (300-3000 m/z) trace from UPLC-MS analysis of one-pot acidolysis after STL between peptide **11b** and **12**, gradient 30-80% CH_3CN/H_2O containing 0.1% TFA over 8 min at a flow rate of 0.4 mL/min.

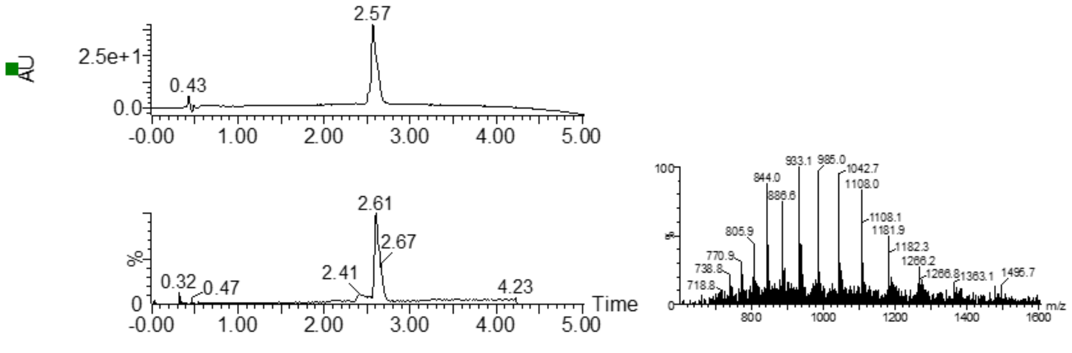

Figure S75. UPLC-MS analysis of purified peptide **13b** (with two R_6 tags). Left: UV (190-400 nm) and MS (300-3000 m/z) trace from UPLC-MS analysis of purified **13b**, gradient 25-95% CH_3CN/H_2O containing 0.1% TFA over 5 min at a flow rate of 0.4 mL/min; Right: ESI-MS calcd. for $C_{782}H_{1301}N_{233}O_{216}S_9$ (Molecular Weight: 17711.9650): $[M+16H]^{16+}$ *m/z* = 1118.5, found 1118.4; $[M+17H]^{17+}$ *m/z* = 1052.8, found 1052.7; $[M+18H]^{18+}$ *m/z* = 994.3, found 994.1; $[M+19H]^{19+}$ *m/z* = 942.1, found 942.3; $[M+20H]^{20+}$ *m/z* = 895.0, found 894.7; $[M+21H]^{21+}$ *m/z* = 852.4, found 852.7; $[M+22H]^{22+}$ *m/z* = 813.7, found 813.3; $[M+23H]^{23+}$ *m/z* = 778.4, found 778.2.

6.3 Folding of IL-2 analogs

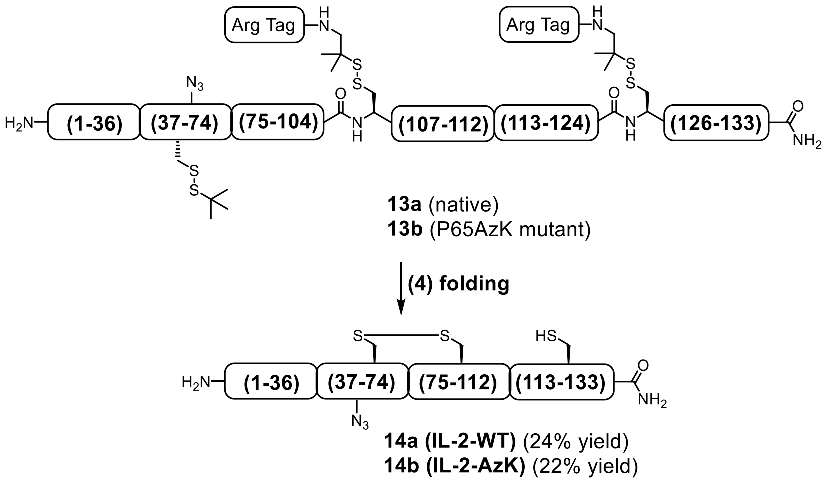

Step 1 (denaturation and reduction):

13a or **13b** was dissolved in buffer containing 6M GnHCl, 0.1M Tris, 30 mM GSH (pH 8.0) at a concentration of 0.1 mg/mL, then incubated at 50 °C for 2h.

Step 2 (dialysis):

The linear protein solution was transferred to a dialysis tube (MWCO 3500) and dialyzed against folding buffer (2M Gn, 0.1M tris, 10mM GSH, 1mM GSSG, pH 8.0) at 4°C for 24h. After that, the protein was dialyzed against PBS (pH 7.4) for at least four times,12h in total.

Step 3 (ultrafiltration):

The folded protein was transferred to a Millipore ultrafiltration tube (MWCO 3000) and concentrated by centrifugation. When the protein concentration was about 0.5 mg/mL (by NanoDrop), 10-fold volume of PBS (pH 7.4) was added to dilute the protein and then ultrafiltration, repeat this workflow twice. Protein purity was checked by

UPLC-MS and SDS-PAGE and the secondary structure was confirmed by CD. Following the above procedure, no misfolded protein was observed, and folded **IL-2-WT** (24% yield) and **IL-2-AzK** (22% yield) were obtained for bioactivity evaluation.

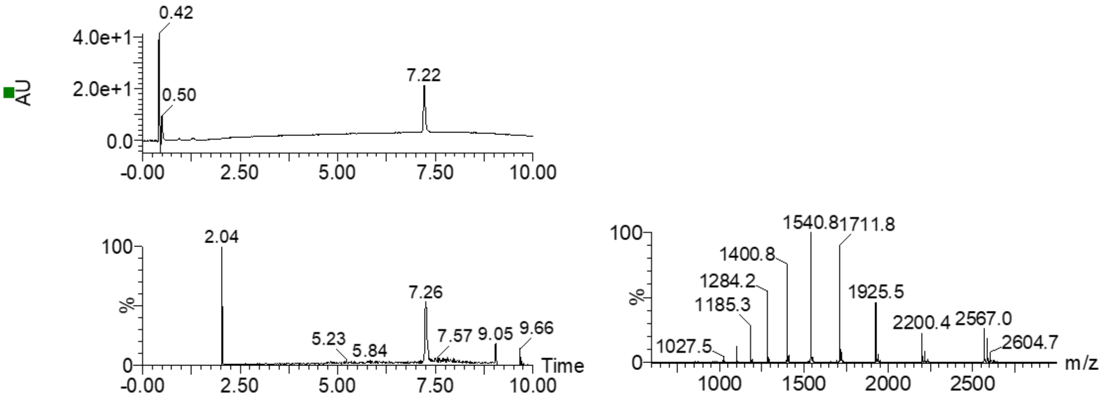

Figure S76. UPLC-MS analysis of folding of **14a** (**IL-2-WT**) after dialysis in PBS and concentrated by ultrafiltration, no HPLC purification, gradient 30-80% CH_3CN/H_2O containing 0.1% TFA over 10 min at a flow rate of 0.4 mL/min.

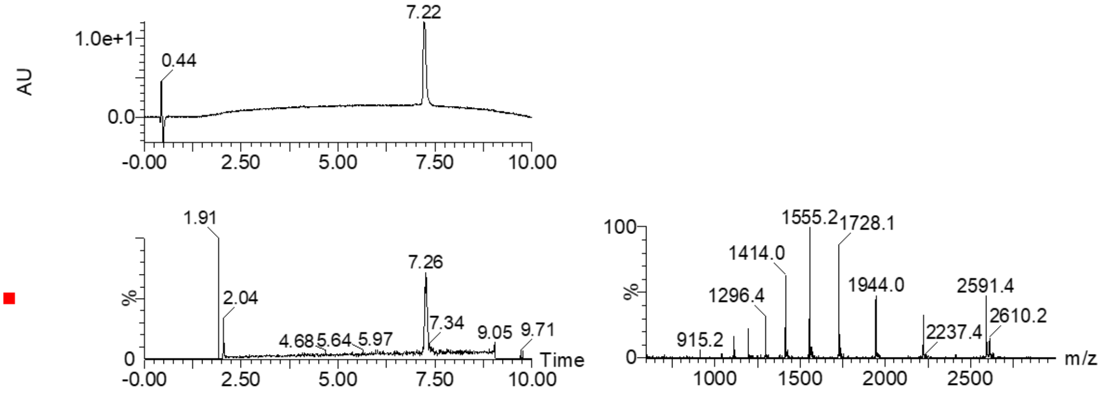

Figure S77. UPLC-MS analysis of folding of **14b** (**IL-2-AzK**) after dialysis in PBS and concentrated by ultrafiltration, no HPLC purification, gradient 30-80% CH_3CN/H_2O containing 0.1% TFA over 10 min at a flow rate of 0.4 mL/min.

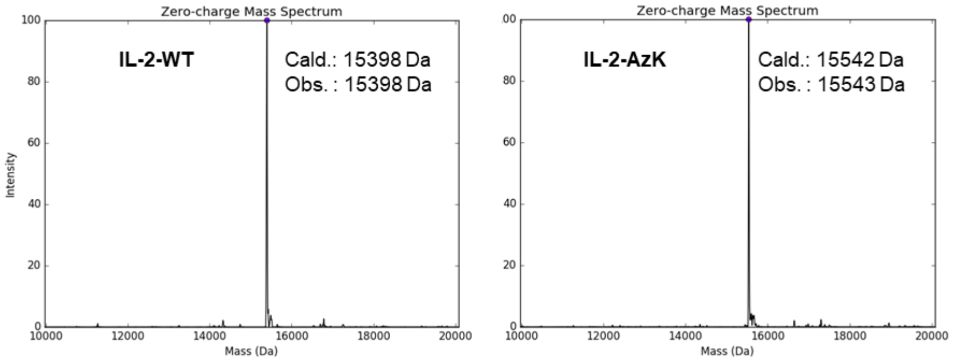

Figure S78. ESI-MS (deconvolution) of **IL-2-WT** and **IL-2-AzK**.

Stepwise RST removal/folding protocol (dilution):

This protocol is the same as the reported protocol for the *in vitro* folding of synthetic IL-2.[4] **13a** was dissolved in reductive denaturation buffer containing 6M GnHCl, 0.1 M Tris and 30 mM GSH (pH 8.0) at a concentration of 0.1 mg/mL, then incubated at 50 ºC for 2h. Subsequently, two-fold volume of GnHCl-free buffer (0.1 M Tris, 1.5 mM GSSG, pH 8.0) was added to protein solution. The folding solution was incubated at room temperature for 24 h.

Simultaneous RST removal/folding protocol (dilution):

In this folding protocol, RST cleavage and protein folding proceed concurrently. Firstly, **13a** was dissolved into denaturation buffer (6M GnHCl, 0.1 M Tris, pH 8.0) at a concentration of 0.1 mg/mL and incubated at 37 ºC for 1 h. Secondly, two-fold volume GnHCl-free buffer (0.1 M Tris, 15 mM GSH, 1.5 mM GSSG, pH 8.0) was added to protein solution. The folding solution was incubated at room temperature for 24 h.

The above two protocols provide similar results, yet HPLC purification is needed to remove misfolded IL-2.

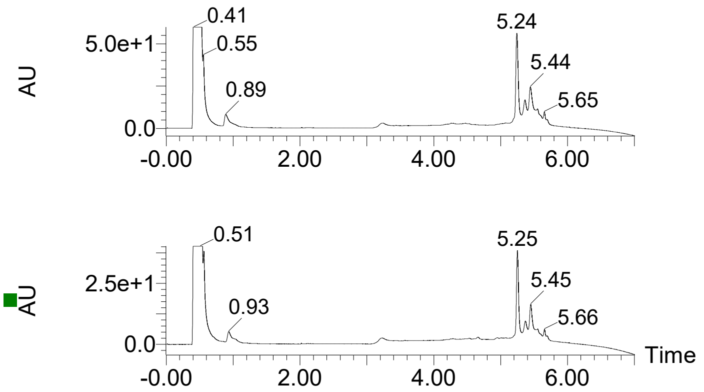

Figure S79. UPLC trace of folding of **13a** (IL-2 with RSTs) by a stepwise reduction/folding protocol (up) or performing tag cleavage and protein folding concurrently (down), gradient 25-95% CH_3CN/H_2O containing 0.1% TFA over 7 min at a flow rate of 0.4 mL/min. Peak at around 5.24 min represents folded IL-2-WT (**14a**).

6.4 SDS-PAGE

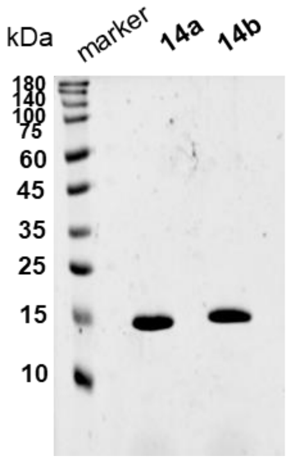

Figure S80. SDS-PAGE of folded **IL-2-WT** (**14a**) and **IL-2-AzK** (**14b**) using reducing condition.

6.5 Circular dichroism

The synthesized **IL-2-WT**, **IL-2-AzK** and HEK-293 expressed human IL-2 were thawed and diluted to around 0.35 mg/mL using PBS (pH 7.4). (protein concentration was determined by NanoDrop UV-Vis spectrophotometer, Thermo Scientific). The CD spectrum was measured by a J-815 circular dichroism spectrometer (JASCO), each sample was scanned for 1 time at room temperature using PBS buffer (pH 7.4) as blank in a 0.1 cm cell. The combined spectra were drawn with Origin 9.

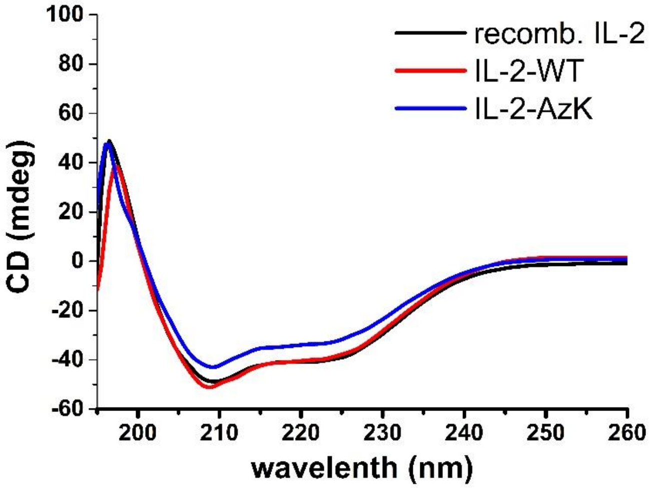

Figure S81. CD of synthetic and recombinant IL-2 proteins.

6.6 Microscale thermophoresis

Freshly folded **IL-2-WT**, **IL-2-AzK** and commercial recombinant IL-2 (ACRO Biosystems) were subjected to MST assay according to the manufacturer's guidelines. Raw data were shown in Table S2 and Table S3. K_d of IL-2 analogs was given by Monolith. The K_d values in this assay were larger than reported value because IL-2Rα used in this assay has an Fc tag which may influence the binding kinetics. The binding between **IL-2-AzK** and IL-2Rα was too weak to be detected by MST, which was consistent with the literature.[5]

Table S2. Results from MST assay between IL-2-WT and IL-2Rα

Protein	Ligand Concentration [M]	Ratio 670nm / 650nm (fraction bound)	Stdev
IL-2-WT	4.82E-10	0.000787454	0.019854537
	9.64E-10	0.01699403	0.013635721
	1.93E-09	0.032492892	0.035237276
	3.85E-09	-0.012239185	0.014961783
	7.71E-09	0.003684046	0.020286564
	1.54E-08	-0.007494124	0.051161596
	3.08E-08	0.041270461	0.027517349
	6.17E-08	0.093475261	0.022442852
	1.23E-07	0.125093468	0.067202248
	2.47E-07	0.251440636	0.015131437
	4.93E-07	0.428168324	0.0252647
	9.87E-07	0.593837354	0.042857674
	1.97E-06	0.68009768	0.033242702
	3.95E-06	0.806854353	0.037397836
	7.90E-06	0.96449587	0.035698895
	1.58E-05	1.509496529	0.04048796

Table S3. Results from MST assay between recombinant IL-2 and IL-2Rα

Protein	Ligand Concentration [M]	Ratio 670nm / 650nm (fraction bound)	Stdev
IL2-H5215, ACRO Biosystems	3.53E-10	0.044093769	0.054287579
	7.07E-10	0.011736146	0.011436058
	1.41E-09	-0.021358441	0.054653658
	2.83E-09	-0.020819092	0.048626779
	5.65E-09	0.041972947	0.038084168
	1.13E-08	-0.004545339	0.106892332
	2.26E-08	0.101043817	0.031727499
	4.52E-08	0.003552382	0.149694955
	9.05E-08	0.164703088	0.067514463
	1.81E-07	0.185160378	0.046759895
	3.62E-07	0.292974694	0.117469504
	7.24E-07	0.75672755	0.084741665
	1.45E-06	0.710757051	0.12632183
	2.90E-06	0.729920539	0.20321009
	5.79E-06	0.978518266	0.174309564
	1.16E-05	2.349132427	0.093689042

7. CTLL-2 Proliferation Assay for Characterization of IL-15 and IL-2

Reagents

• CTLL-2 cells (ATCC)

• Fetal bovine serum (Thermo fisher)

• RPMI 1640 medium (Thermo fisher)

• Human IL-15, premium grade (MedChemExpress, Cat. No. HY-P7034)

• Human IL-2 (ACROBiosystems, Cat. No. IL2-H5215)

• WST-1 Cell Proliferation and Cytotoxicity Assay Kit (Beyotime)

Materials

• CO_2 Incubator

• Cell counter

• Pipette Tips and Pipettes (including a multichannel Pipette)

• Centrifuge

• Centrifuge tube

• 96-well plate

• Microplate Reader

Experimental procedure

1. Culture CTLL-2 cells in RPMI1640 medium with 10% of FBS and 30 ng/mL IL-2 in the CO_2 incubator (at 37°C, 5% CO_2).

2. Harvest the cells and wash once by RPMI1640 medium with 10% of FBS.

3. Count the cells number and the viability, then adjust the cell density to around 1×10^6 (for IL-15) or 2×10^5 (for IL-2) cells/mL. Seed 50 μL of the cell suspension in 96-well-plate. The final cell density is 5×10^4 per well for IL-15 and 1×10^4 for IL-2.

4. Prepare IL-15/IL-2 sample solution at serial concentrations, then add 50uL of each dilution to 96-well-plate with CTLL-2 cells.

5. Incubate the 96-well-plate at 37°C, 5% CO_2 for 48 h.

6. Add 10 μL of WST-1 Reagent to each well, then mix the contents by shaking gently.

7. Allow the plate to incubate at room temperature for 4 h to stabilize luminescent signal.

8. Record luminescence by Microplate Reader.

Data processing

EC_{50} of IL-15 or IL-2 proteins were determined by GraphPad Prism 10 analysis of raw data from three parallel experiments using nonlinear regression: log(agonist) vs. response--Variable slope (four parameters).

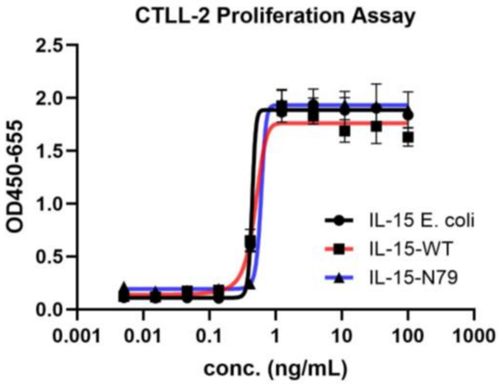

Protein	EC_{50} (ng/mL)
IL-15-E. coli	0.44
IL-15-WT	0.53
IL-15-N79	0.60

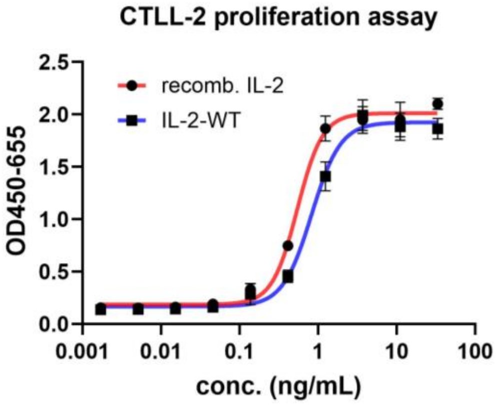

Protein	EC_{50} (ng/mL)
IL-2-recomb.	0.54
IL-2-WT	0.82

Figure S82. Dose-dependent response of IL-15 (up) and IL-2 (down) proteins in CTLL-2 proliferation assay.

8. NMR Data

^{1}H NMR in $CDCl_3$

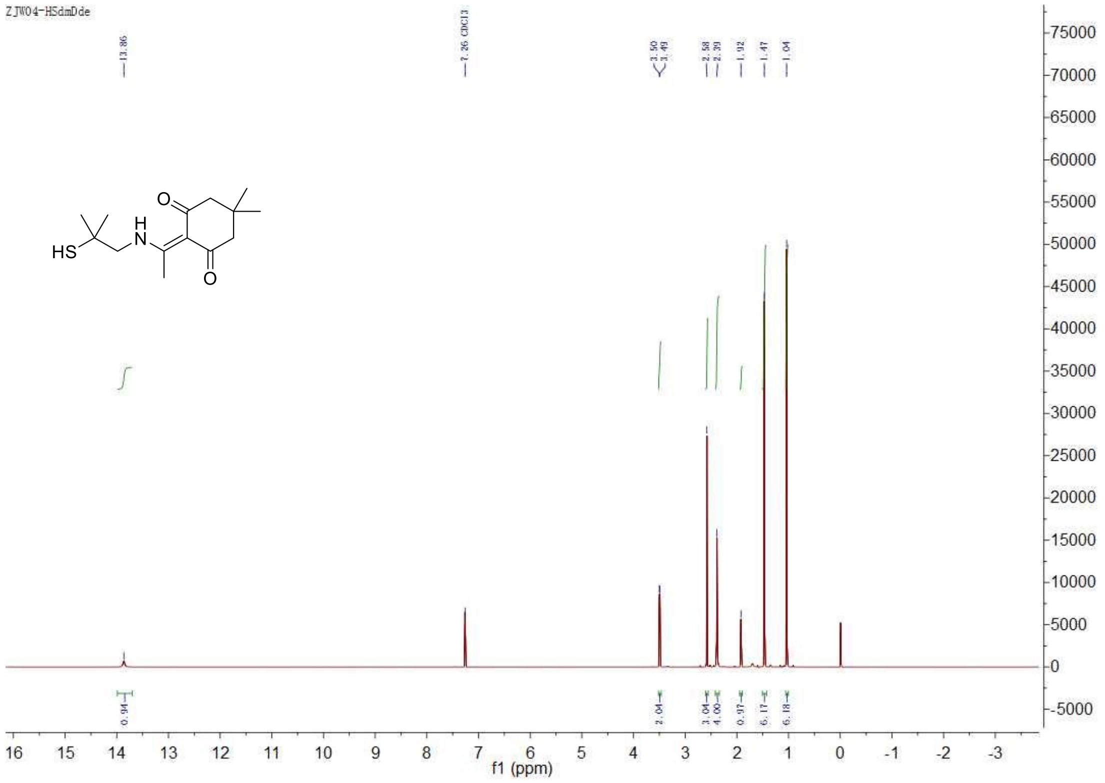

^{13}C NMR in $CDCl_3$

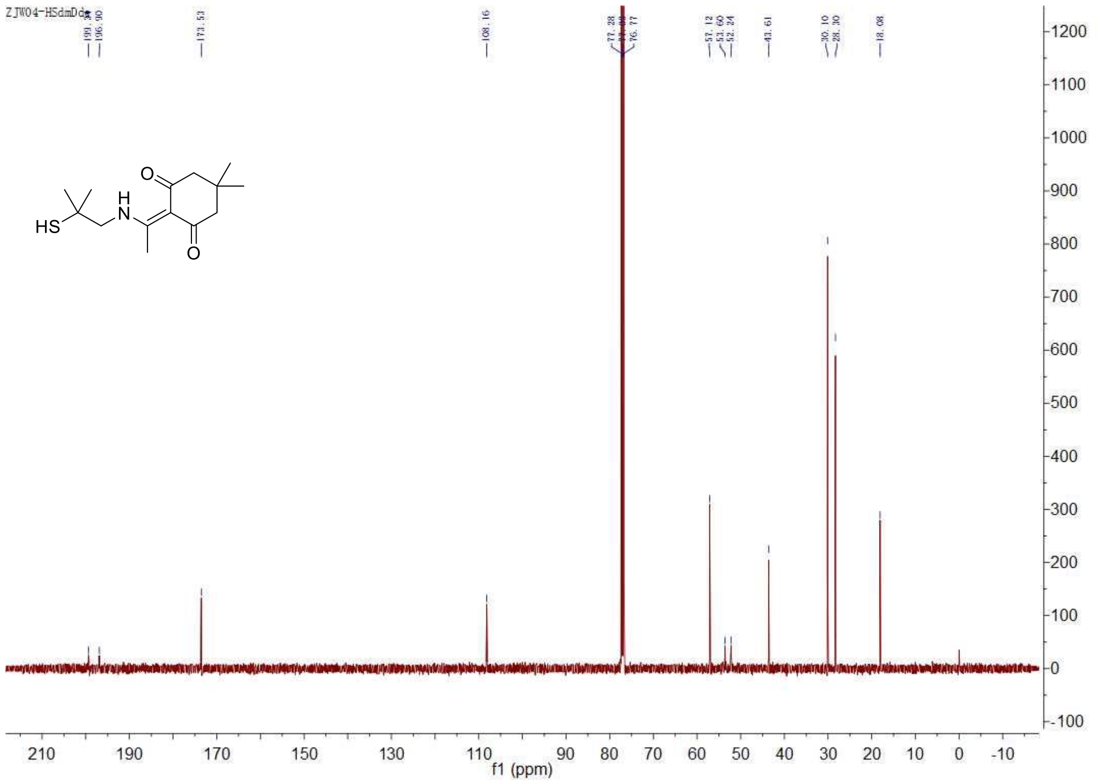

^{1}H NMR in $CDCl_3$

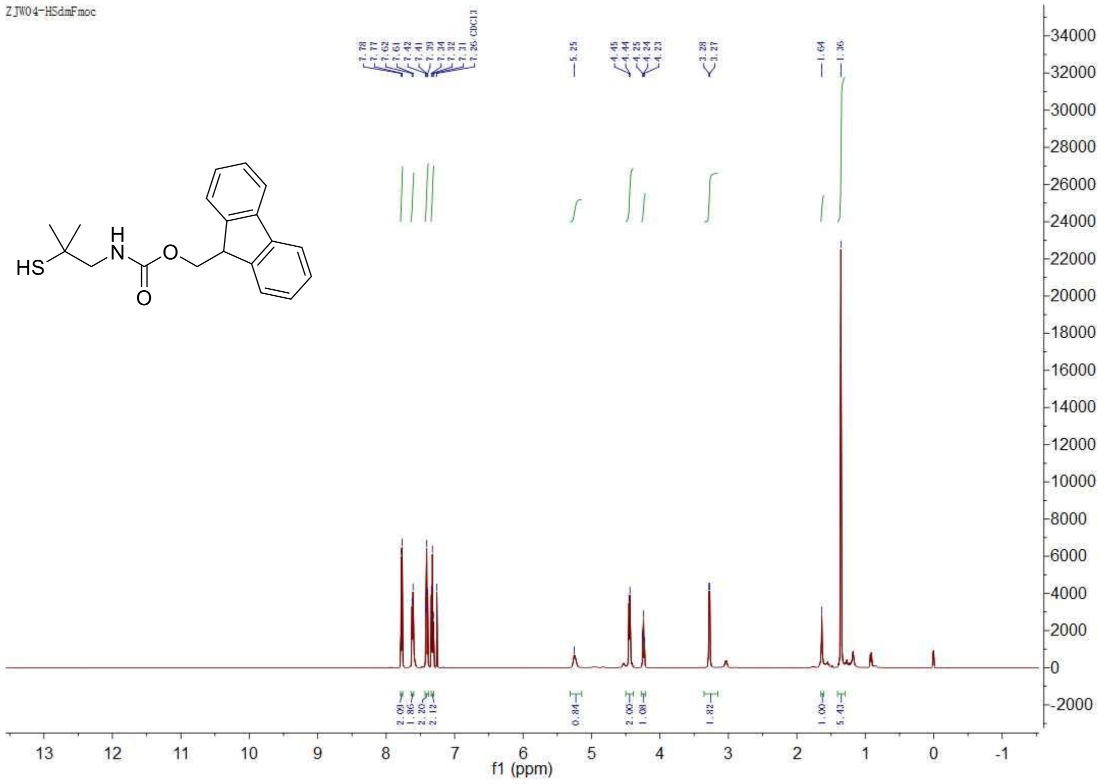

^{13}C NMR in $CDCl_3$

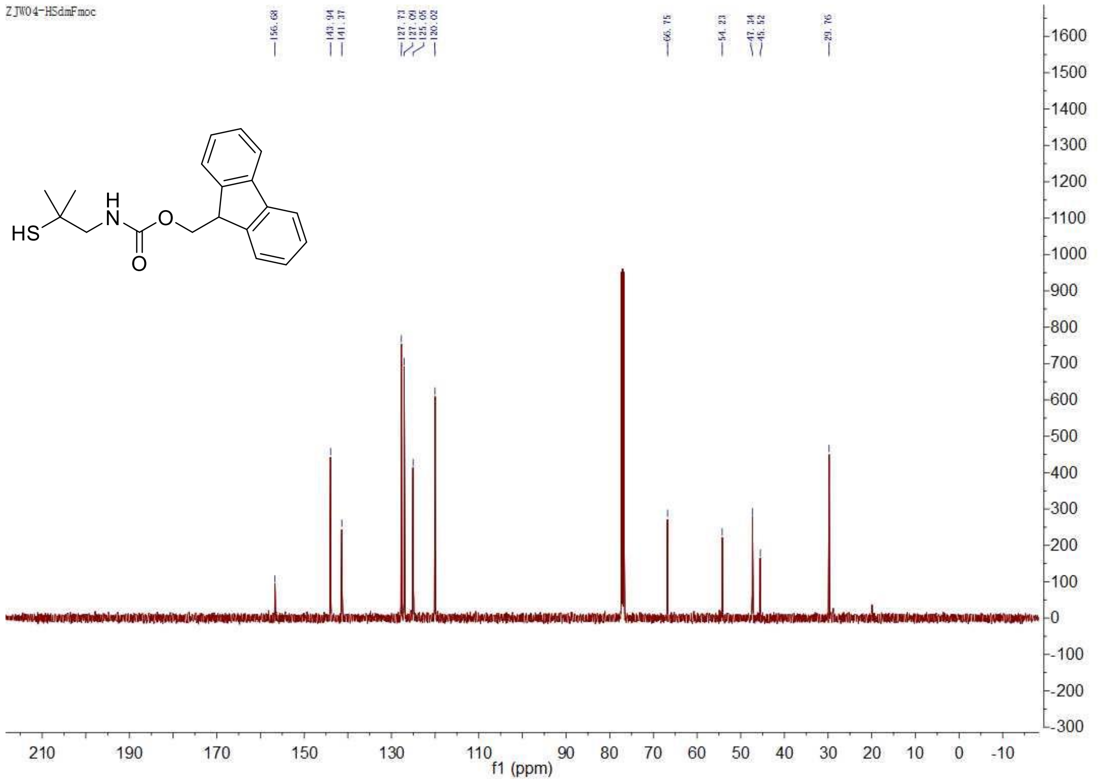

^{1}H NMR in *d6*-DMSO

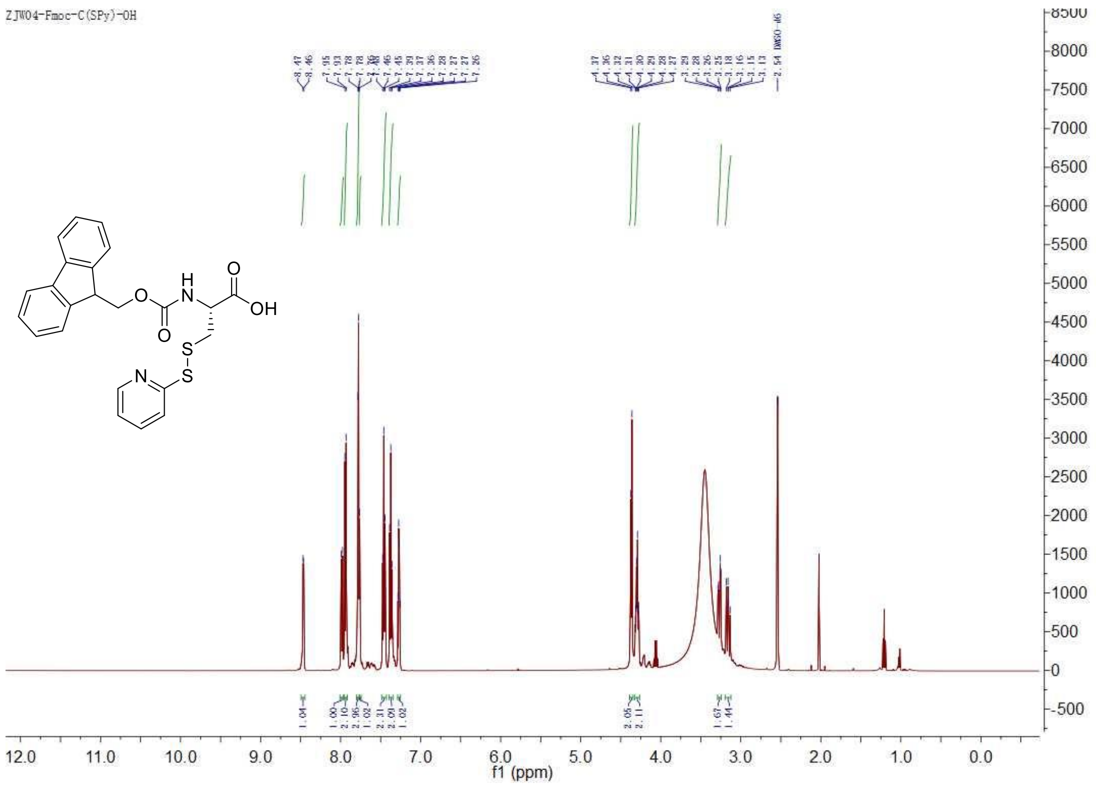

^{13}C NMR in *d6*-DMSO

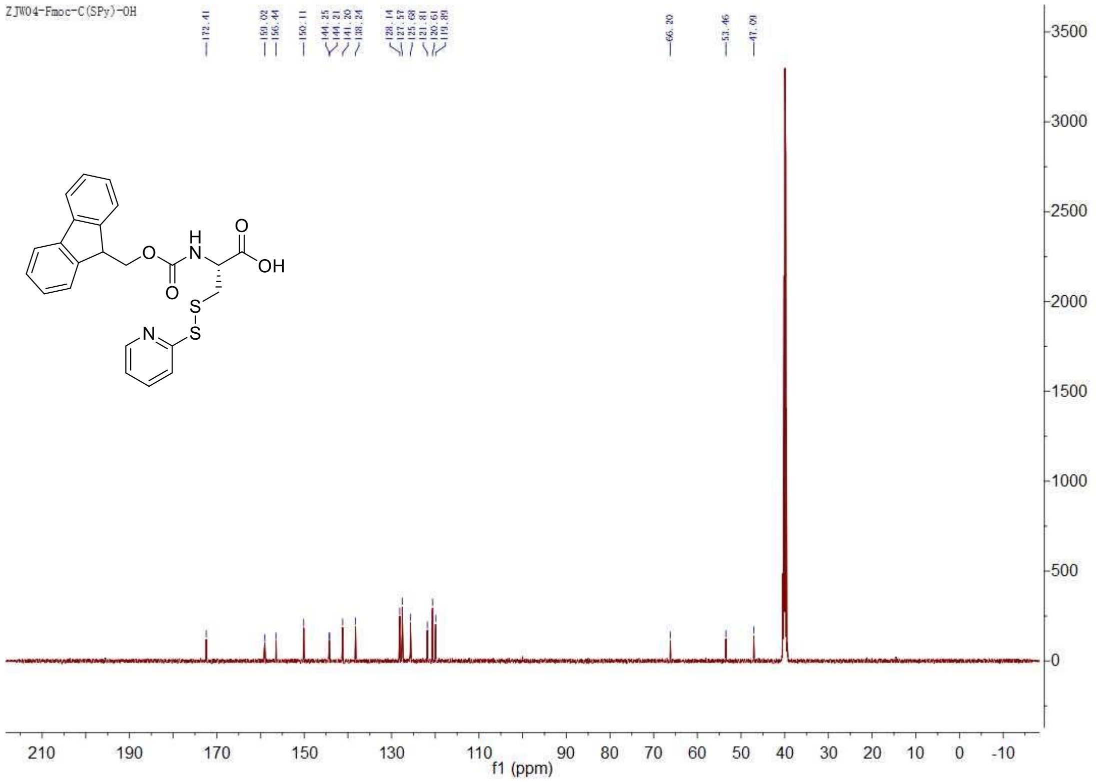

^{1}H NMR in *d6*-DMSO

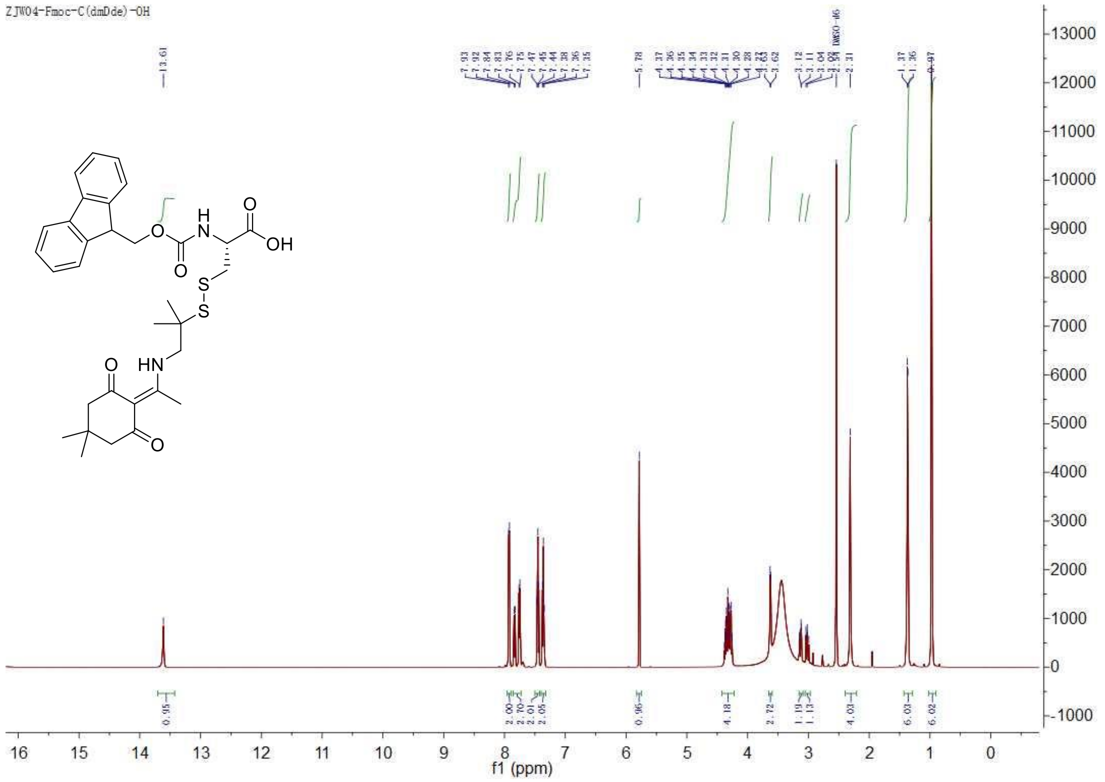

^{13}C NMR in *d6*-DMSO

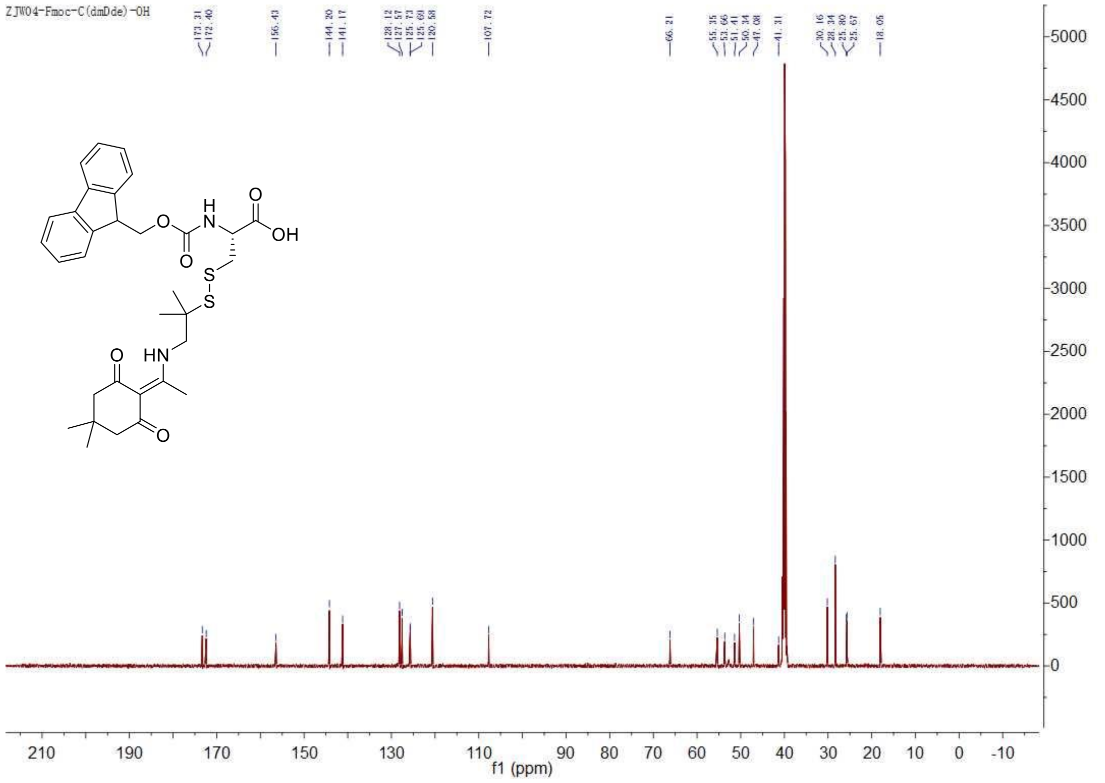

^{1}H NMR in *d6*-DMSO

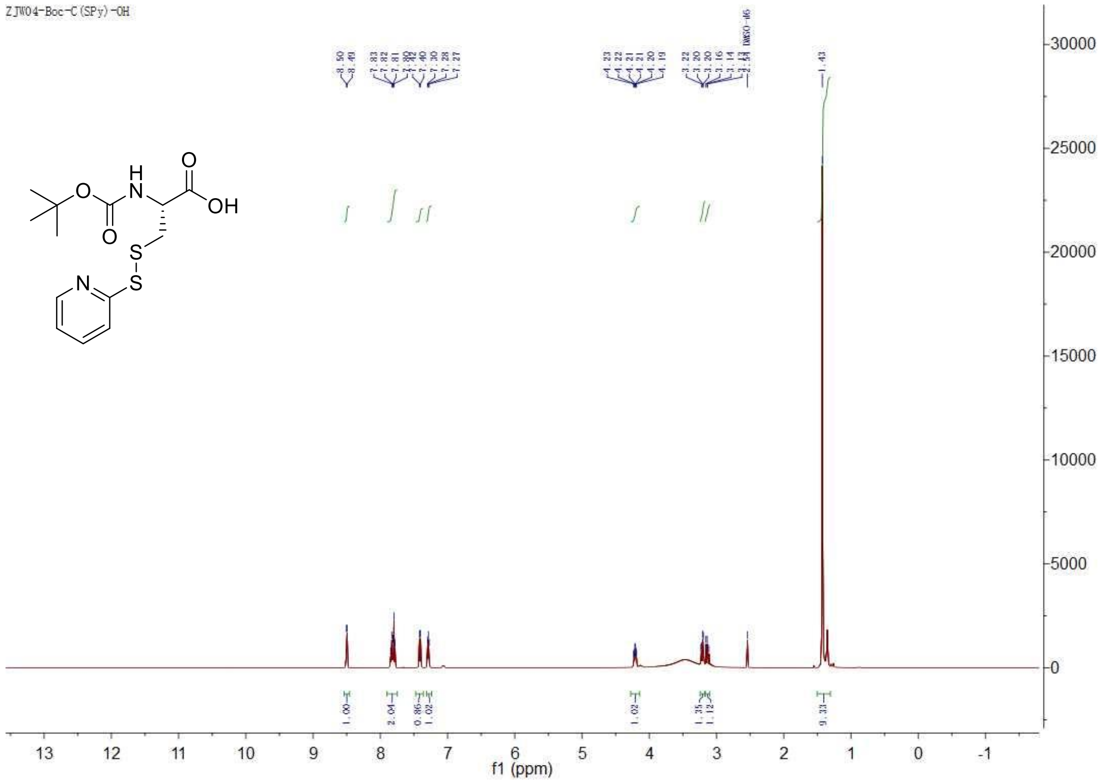

^{13}C NMR in *d6*-DMSO

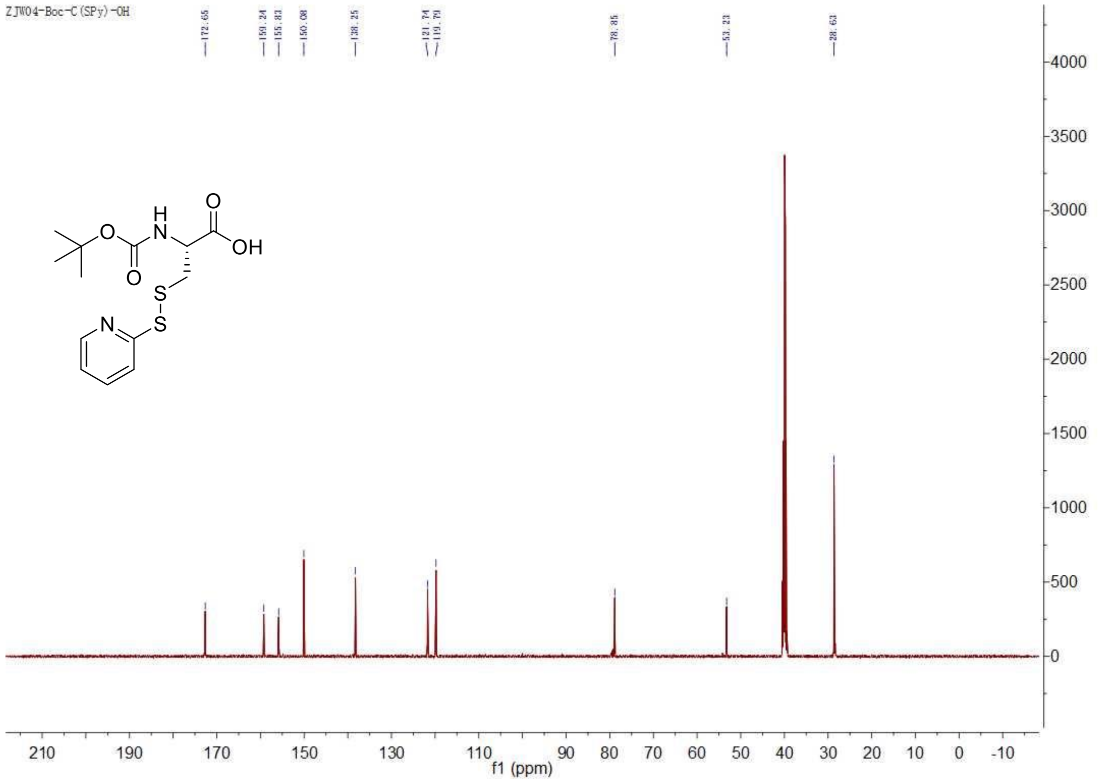

^{1}H NMR in $CDCl_3$

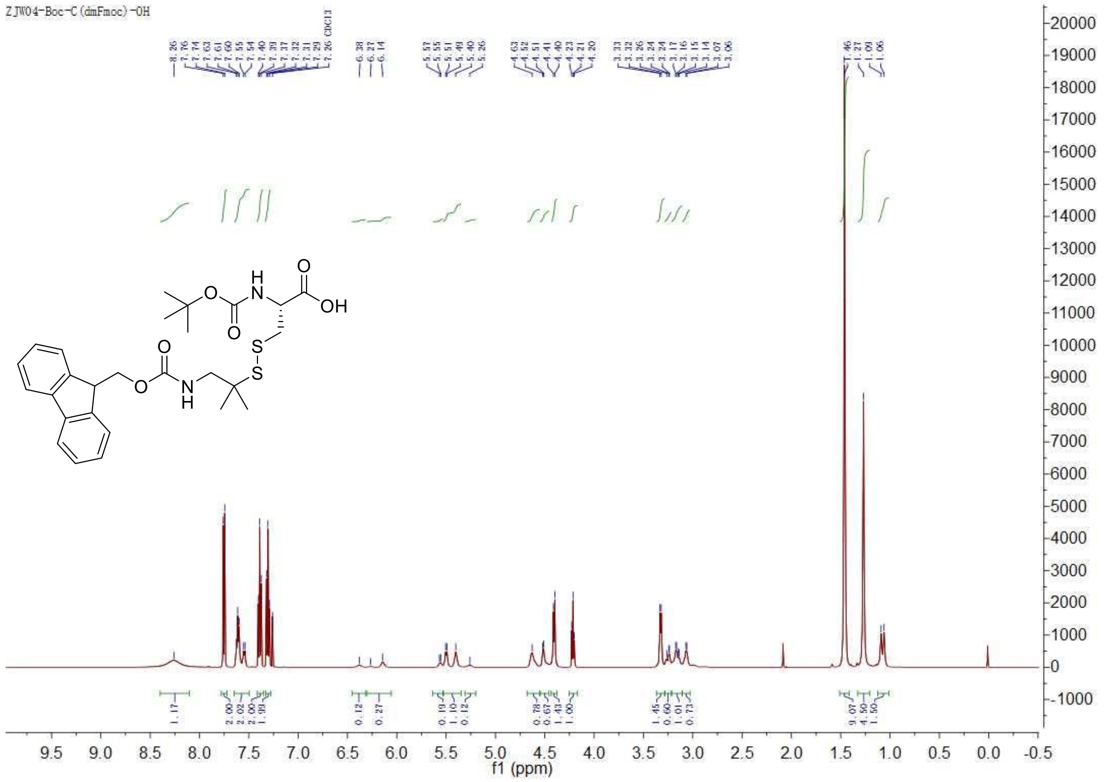

^{13}C NMR in $CDCl_3$

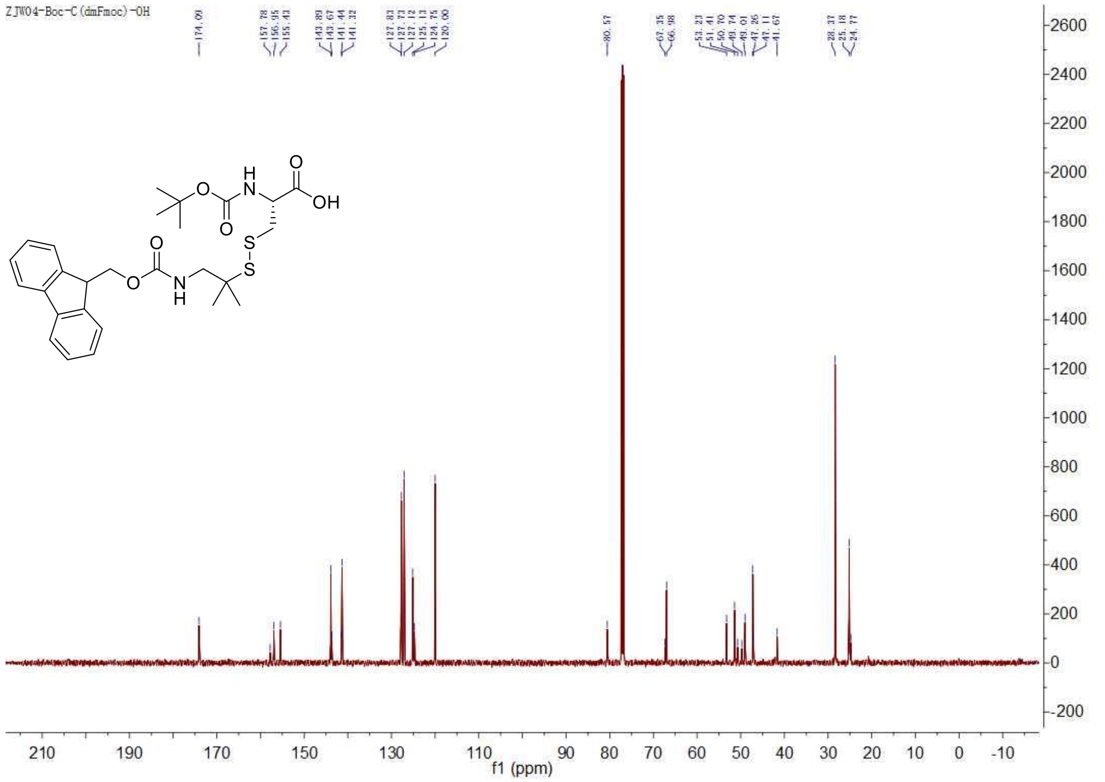

^{1}H NMR in $CDCl_3$

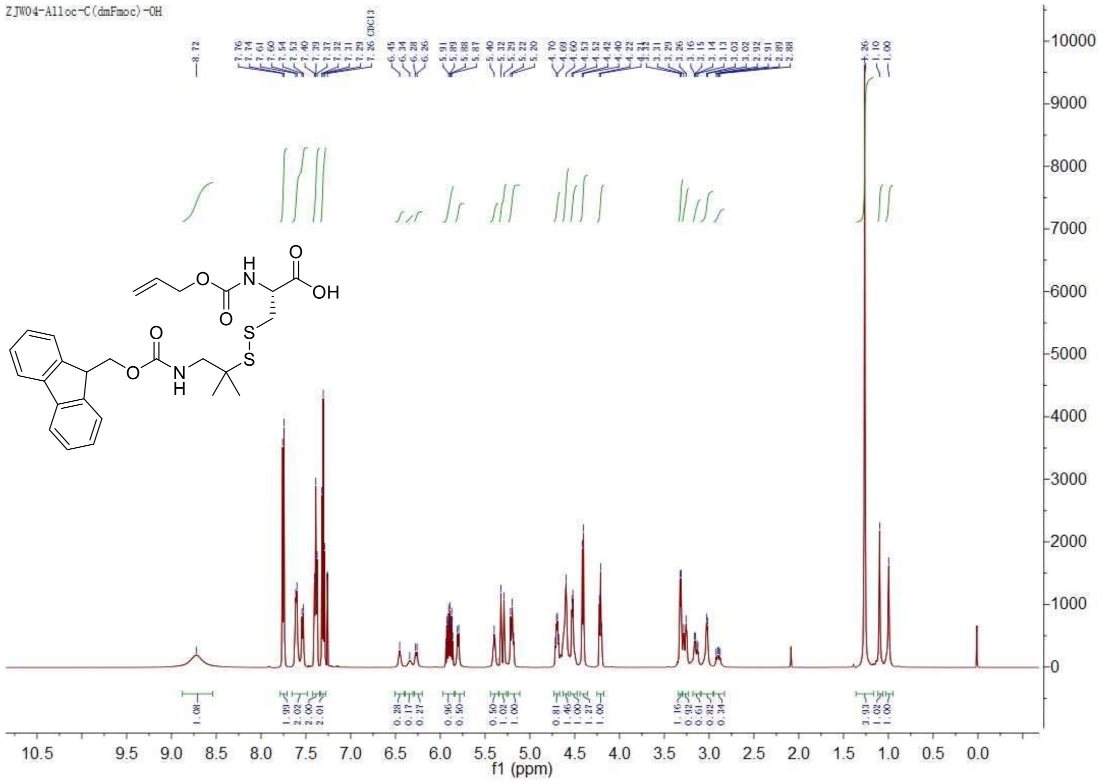

^{13}C NMR in $CDCl_3$

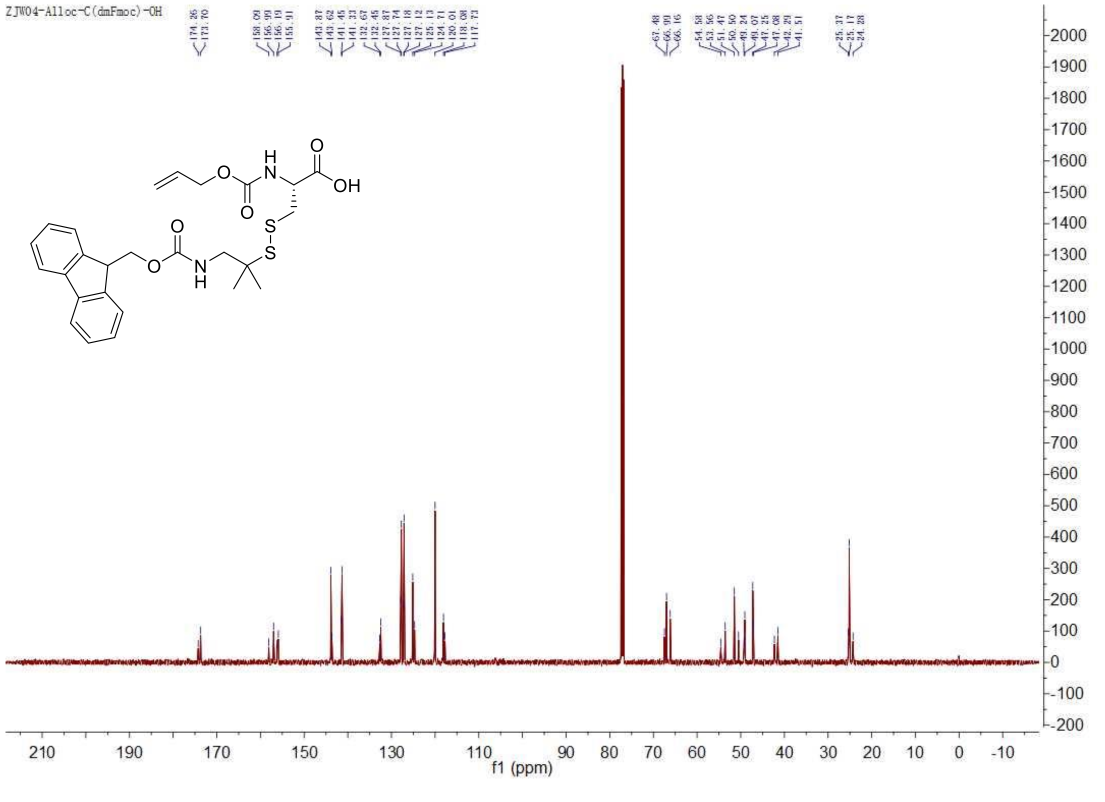

^{1}H NMR in *d6*-DMSO

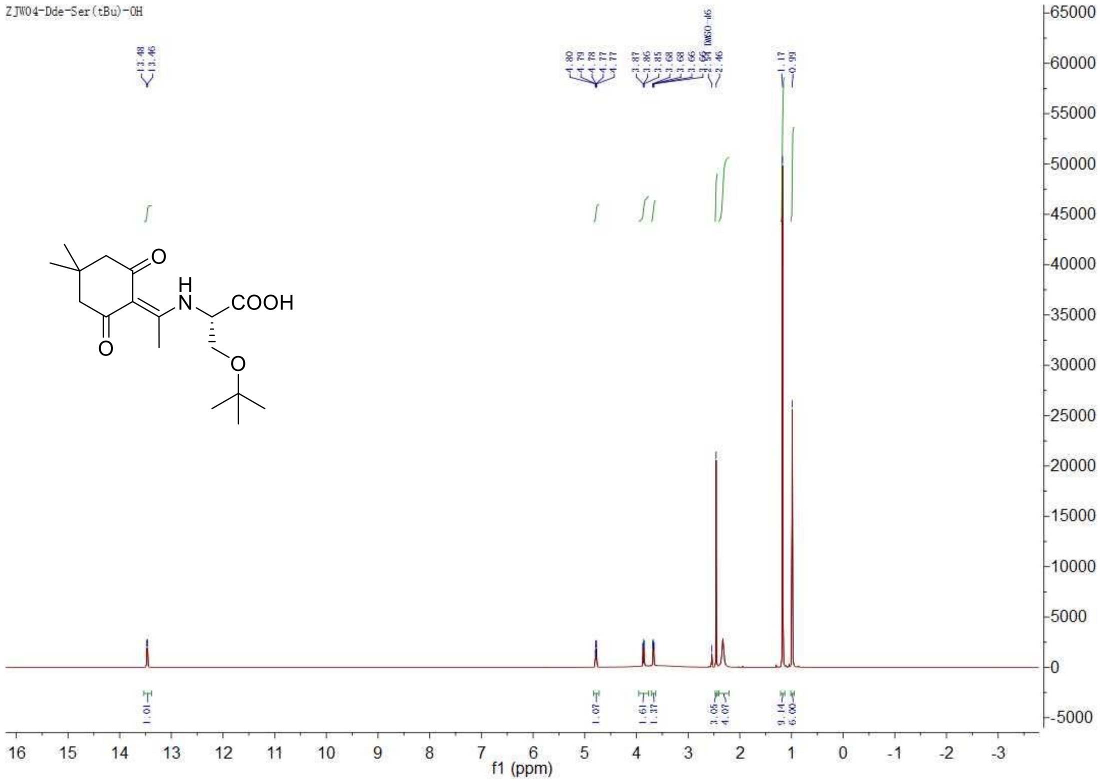

^{13}C NMR in *d6*-DMSO

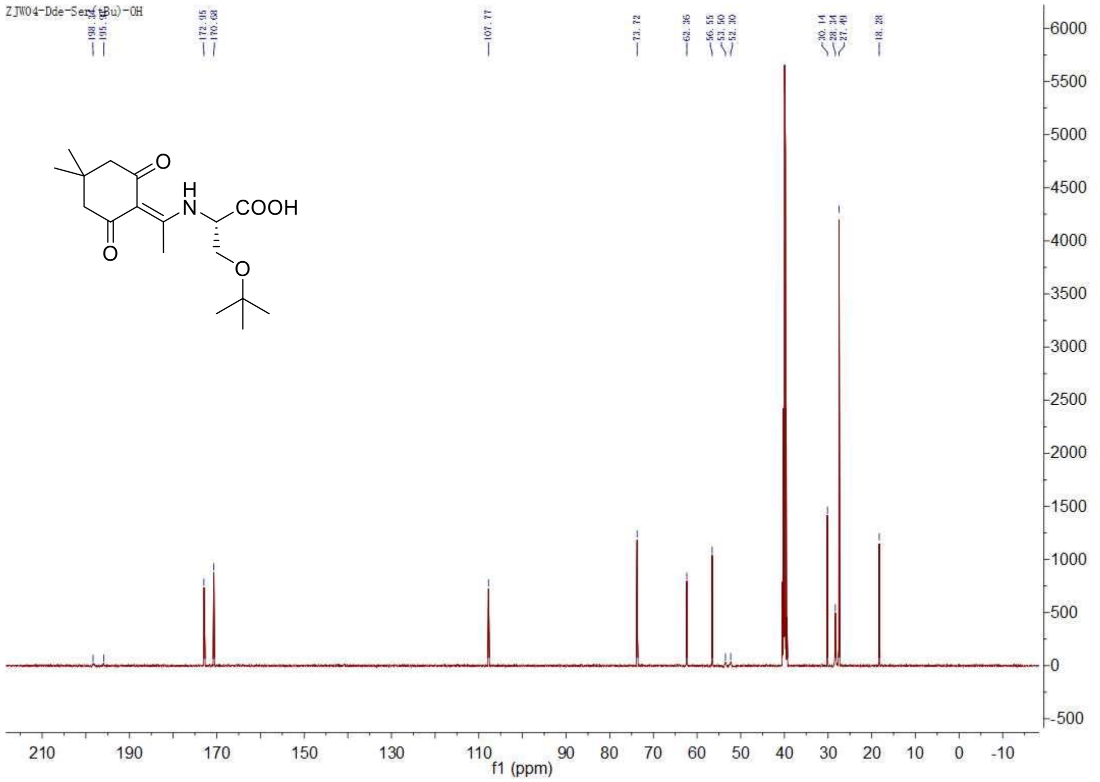

9. Reference

(1) Lee, C. L.; Liu, H.; Wong, C. T.; Chow, H. Y.; Li, X., Enabling N-to-C Ser/Thr Ligation for Convergent Protein Synthesis via Combining Chemical Ligation Approaches. *J. Am. Chem. Soc.* **2016,** *138* (33), 10477-84.

(2) Wu, H.; Sun, Z.; Li, X., Selective Peptide Cysteine Manipulation on Demand and Difficult Protein Chemical Synthesis Enabled by Controllable Acidolysis of N,S-Benzylidene Thioacetals. *Angew. Chem., Int. Ed.* **2024,** *63* (19), e202403396.

(3) Huang, W.; Li, J.; Wang, L. X., Unusual transglycosylation activity of Flavobacterium meningosepticum endoglycosidases enables convergent chemoenzymatic synthesis of core fucosylated complex N-glycopeptides. *Chembiochem* **2011,** *12* (6), 932-41.

(4) Wu, H.; Tan, Y.; Ngai, W. L.; Li, X., Total synthesis of interleukin-2 via a tunable backbone modification strategy. *Chem. Sci.* **2023,** *14* (6), 1582-1589.

(5) Ptacin, J. L.; Caffaro, C. E.; Ma, L. N.; Gall, K. M. S.; Aerni, H. R.; Acuff, N.; Herman, R. W.; Pavlova, Y.; Pena, M. J.; Chen, D. B.; Koriazova, L. K.; Shawver, L. K.; Joseph, I. B.; Milla, M. E., An engineered IL-2 reprogrammed for anti-tumor therapy using a semi-synthetic organism. *Nat. Commun.* **2021,** 12 (1).
